# Design, Synthesis and Antifungal Activity of Psoralen Derivatives

**DOI:** 10.3390/molecules22101672

**Published:** 2017-10-09

**Authors:** Xiang Yu, Ya Wen, Chao-Gen Liang, Jia Liu, Yu-Bin Ding, Wei-Hua Zhang

**Affiliations:** Jiangsu Key Laboratory of Pesticide Science, Department of Chemistry, College of Sciences, Nanjing Agricultural University, Nanjing 210095, China; 2015811024@njau.edu.cn (X.Y.); 2016111016@njau.edu.cn (Y.W.); 2016111015@njau.edu.cn (C.-G.L.); 2013111007@njau.edu.cn (J.L.); ybding@njau.edu.cn (Y.-B.D.)

**Keywords:** linear furanocoumarin, synthesis, antifungal activity, structure-activity relationship

## Abstract

A series of linear furanocoumarins with different substituents have been designed and synthesized. Their structures were confirmed by ^1^H-NMR spectroscopy, high resolution mass spectra (EI-MS), IR, and X-ray single-crystal diffraction. All of the target compounds were evaluated in vitro for their antifungal activity against *Rhizoctorzia solani*, *Botrytis cinerea*, *Alternaria solani*, *Gibberella zeae*, *Cucumber anthrax*, and *Alternaria* leaf spot at 100 μg/mL, and some of the designed compounds exhibited potential antifungal activities. Compound **3a** (67.9%) exhibited higher activity than the control Osthole (66.1%) against *Botrytis cinerea*. Furthermore, compound **4b** (62.4%) represented equivalent antifungal activity as Osthole (69.5%) against *Rhizoctonia solani*. The structure-activity relationship (SAR) study demonstrates that linear furanocoumarin moiety has an important effect on the antifungal activity, promoting the idea of the coumarin ring as a framework that might be exploited in the future.

## 1. Introduction

Plant diseases cause severe crop yield reduction and result in significant economic losses every year. How to control them in modern agriculture is still a big challenge [1,2]. Although many chemical agents were developed and applied to control these diseases, most of them cannot fully protect the crops or completely cure the crops’ tissues from fungal infection under field conditions. The botanical fungicide is one of the plant protection alternatives, generally considered safe for the environment and health. The development botanical fungicide is important [3].

**Figure 1:**
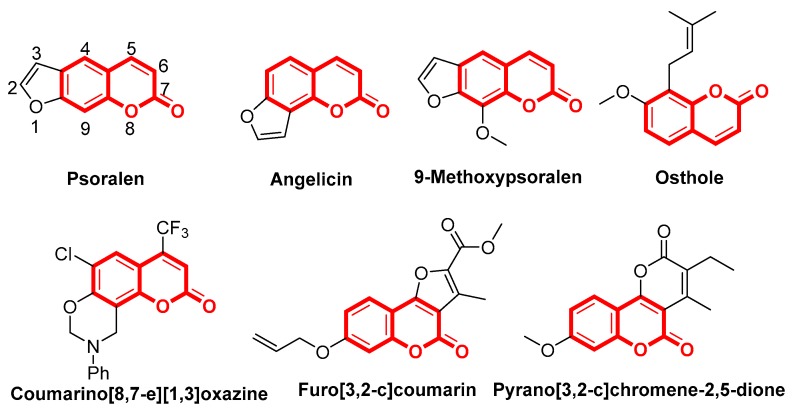
Structures of coumarin-containing compounds.

Coumarins widely exist in nature and can be found in all parts of plants, especially in grasses, orchids, citrus fruits, and legumes [4]. Furanocoumarins are one of the main groups in coumarins, based on their chemical structure, they can be generally classified as linear (e.g., Psoralen, Figure 1) and angular (e.g., Angelicin, Figure 1) type. Angular furanocoumarins are always present together with linear furanocoumarins, but in lower contents [5]. As a structural core, furanocoumarins is used regularly as a scaffold in medicinal and agricultural chemistry, this is highlighted by *Angelica dahurica* and Psoralen (Figure 1), the traditional Chinese herbs, usually possess a broad scope of pharmacological and biochemical activities, including anti-Alzheimer’s disease, anticancer [6], anti-HIV (human immunodeficiency virus), antitumor [7], antitumour, antidiabetic [8], anti-inflammatory, antidepressant [9], antiprotozoal, insecticidal [10], antibacterial [11], and antifungal [12,13] activities, and they are active photosensitizers for the treatment of several skin diseases [14,15,16].

**Scheme 1:**
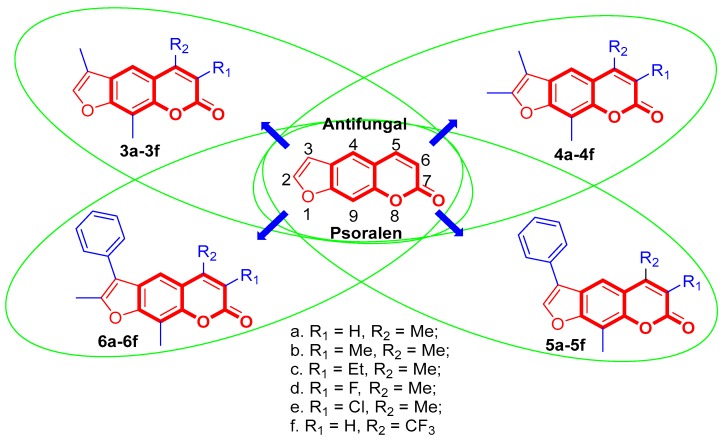
The structures of the designed and synthesized compounds.

To date, in order to decrease toxicity and explore the potential biological activity of linear furanocoumarins there has been accomplished in three different ways: first, using angular furanocoumarins, which on account of their geometry cannot crosslink with DNA; second, blocking of the photo reactive α-pyrone double bond by appropriate substituents or by annelation of an additional aromatic ring; third, incorporating an additional benzene ring between active double bonds of the a-pyrone and furan moiety [16]. Based on our previous work (Figure 1) [12,13,17,18,19,20], a series of different substituted linear furanocoumarins were designed and synthesized by construction of a furan ring on the benzene moiety of coumarin (Scheme 1). To the best of our knowledge, there was less research systematically investigated the antifungal activity of linear furanocoumarins against plant pathogenic fungi. Aiming to discover promising botanical candidates, we screened the antifungal activity of the synthesized linear furanocoumarins against six botanical fungi, including *Rhizoctorzia solani*, *Botrytis cinerea*, *Alternaria solani*, *Gibberella zeae*, *Cucumber anthrax* and *Alternaria* leaf spot, which are often encountered in plants.

## 2. Materials and Methods

### 2.1. Chemicals and Methods

**Figure 2:**
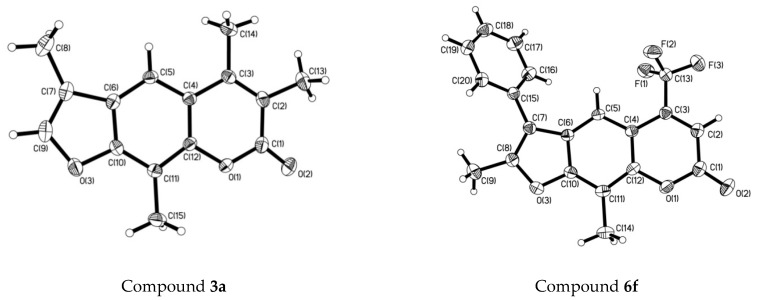
X-ray single crystal structures of compounds **3a** and **6f**.

All materials were obtained from commercial sources and used as received. The evidence for the formation of all the synthesized compounds can be achieved by the melting point, ^1^H-NMR, ^13^C-NMR, high resolution mass spectra (HR-MS) and IR spectra. Melting points were obtained on a melting-point apparatus (BUCHI, Flawil, Switzerland) and are uncorrected. NMR spectra were performed on a Bruker DRX-400 instrument (Bruker, Karlsruhe, Germany) in CDCl_3_ or DMSO-*d*_6_ with TMS as the internal reference (400 and 100 MHz for ^1^H-NMR and ^13^C-NMR respectively). Infrared spectra were recorded on a Bruker Tensor 27 spectrometer, and samples were prepared as KBr plates. HR-MS were acquired in positive mode on a JMS-AX505HA (JEOL, Akishima, Japan), and the detailed physical and analytical data are listed in the Supplementary Information. The course of reactions and the purity of products were monitored by thin-layer chromatography (TLC) using silica gel GF/UV 254 (YUHUA, Gongyi, China). Reaction yields were not optimized. The single-crystal structures of compound **1a** and **6f** were determined by X-ray crystallography as illustrated (Figure 2), respectively.

In this study, a series of psoralen derivatives (compounds **3a**–**3f**, **4a**–**4f**, **5a**–**5f**, and **6a**–**6f**) were designed and synthesized by forming a furan ring on the benzene moiety of coumarin. Aiming to improve the levels of antifungal activity, we substituted the hydrogen on C-2 and C-3 positions for methyl or phenyl to block the double bond on furan moiety. In addition, to enrich the compound group, the hydrogen on C-5 and C-6 positions were substituted by methyl, ethyl, trifluoromethyl, fluorine, chlorine, or formed with an additional hexatomic ring on the both position.

#### 2.1.1. General Procedure for the Preparation of Compounds **2a**–**2f**

**Scheme 2:**
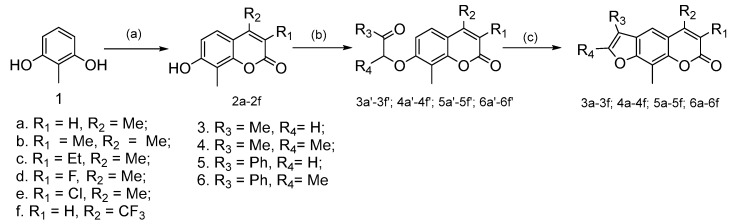
Synthetic routes for the target compounds. Reagents and conditions: (a) R_4_COCHR_3_COOCH_2_CH_3_, H_2_SO_4_, 0 °C; (b) α-chloroacetone, hydrous K_2_CO_3_, KI, tetra-*n*-butylammonium bromide (TBAB), hydrous Acetone, 80 °C, 6 h; (c) NaOH, H_2_O, N_2_, reflux, 4 h.

The initial coumarins **2a**–**2f** (Scheme 2) were synthesized from commercially available 2-methylresorcinol and β-ketoester through Pechmann reaction. The mixture of 2-methylresorcinol (100 mmol, 12.41 g) and β-ketoester (ethyl 3-oxobutanoate) (120 mmol, 13.26 g) were dropwise into the concentrated sulfuric acid at iced water with stirring for 12 h, and the crude product was recrystallized to generate compound **2a**. Yields for compounds **2a**–**2f** vary from 30% to 60%.

#### 2.1.2. Preparation of 2-Bromo-1-phenylpropan-1-one

**Scheme 3:**
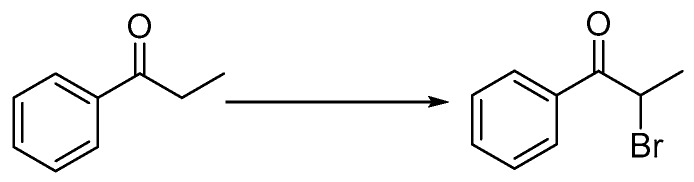
The synthetic method for the preparation of 2-bromo-1-phenylpropan-1-one. Reagents and conditions: 30% H_2_O_2_, 40% HBr, r.t., 24 h.

All the α-chloroacetone used in the Scheme 2 (b) except 2-bromo-1-phenylpropan-1-one was obtained from commercial sources without further purification. 2-bromo-1-phenylpropan-1-one was synthesized through the reported method [21,22] (Scheme 3).

#### 2.1.3. General Procedure for the Preparation of Compounds **3a′**–**3f′**, **4a′**–**4f′**, **5a′**–**5f′** and **6a′**–**6f′**

Ether derivatives (compounds **3a′**–**3f′**, **4a′**–**4f′**, **5a′**–**5f′**, and **6a′**–**6f′**, shown in Scheme 2) were synthesized by etherification from compounds **2a**–**2f** through Williamson conditions [23], compound **2a** (10 mmol, 1.90 g) was dissolved in acetone at reflux. K_2_CO_3_ (30 mmol, 4.15 g), tetra-*n*-butylammonium bromide (TBAB, 0.2 equiv., 0.64 g) and KI (10 mmol, 1.66 g) were added gradually with stirring for 15 min, and then 1-chloropropan-2-one (10 mmol, 0.92 g) was added to the system. The mixture was continuing stirred at reflux for 6 h. After the reaction solution cooling, the filtrate was concentrated under reduced pressure and purified by recrystallization to generate compound **3a′**. Yields for compounds **3a′**–**3f′**, **4a′**–**4f′**, **5a′**–**5f′**, and **6a′**–**6f′** vary 50% to 90%.

#### 2.1.4. General Procedure for the Preparation of Compounds **3a**–**3f**, **4a**–**4f**, **5a**–**5f**, and **6a**–**6f**

Thereafter, cyclization of oxo ether derivative compound **3a′** (5 mmol, 1.20 g) was accomplished by heating with strong alkaline solution in the dark for 3 h under N_2_ protection. The solution was diluted with iced water, and acidified with 10% HCl solution. The precipitate obtained was collected and crystallized from MeOH to generate compound **3a**. Yields for the furanocoumarin derivatives compounds **3a**–**3f**, **4a**–**4f**, **5a**–**5f** and **6a**–**6f** vary 60% to 90%.

*3,5,9-Trimethyl-7H-furo[3,2-g]chromen-7-one* (**3a**): White solid; m.p.: 183.2~183.7 °C; Yield: 83.1%; ^1^H-NMR (400 MHz, CDCl_3_) δ 7.54 (s, 1H), 7.51 (d, *J* = 23.4 Hz, 2H), 6.26 (s, 1H), 2.59 (s, 3H), 2.52 (s, 3H), 2.29 (d, *J* = 1.0 Hz, 3H); ^13^C-NMR (100 MHz, CDCl_3_) δ 161.43, 155.80, 153.19, 149.41, 142.81, 125.25, 115.99, 115.89, 112.80, 111.72, 109.56, 19.32, 8.48, 7.93; IR (KBr) ν/cm^−1^: 3100, 2917, 1707, 1594, 1383, 1102, 859, 817, 758; HR-MS (ESI): *m*/*z* calcd for C_14_H_12_O_3_ ([M + H]^+^) 229.0865, found 229.0857.

*3,5,6,9-Tetramethyl-7H-furo[3,2-g]chromen-7-one* (**3b**): White solid; m.p.: 219.4~219.6 °C; Yield: 94.3%; ^1^H-NMR (400 MHz, DMSO) δ 7.86 (s, 1H), 7.79 (s, 1H), 2.46 (s, 6H), 2.26 (s, 3H), 2.12 (s, 3H); ^13^C-NMR (100 MHz, CDCl_3_) δ 162.48, 154.94, 147.97, 146.69, 142.49, 125.02, 119.49, 116.57, 115.86, 111.24, 108.96, 15.58, 13.40, 8.46, 7.94; IR (KBr) ν/cm^−1^: 3103, 1699, 1593, 1378, 1112, 1074, 855, 758; HR-MS (ESI): *m*/*z* calcd for C_15_H_14_O_3_ ([M + H]^+^) 243.1021, found 243.1016.

*6-Ethyl-3,5,9-trimethyl-7H-furo[3,2-g]chromen-7-one* (**3c**): White solid; m.p.: 165.6~165.7 °C; Yield: 93.5%; ^1^H-NMR (400 MHz, CDCl_3_) δ 7.52 (s, 1H), 7.46 (d, *J* = 1.1 Hz, 1H), 2.73 (q, *J* = 7.5 Hz, 2H), 2.58 (s, 3H), 2.50 (s, 3H), 2.28 (d, *J* = 1.1 Hz, 3H), 1.17 (t, *J* = 7.5 Hz, 3H); ^13^C-NMR (100 MHz, CDCl_3_) δ 162.06, 155.02, 148.11, 146.28, 142.51, 125.51, 125.05, 116.79, 115.85, 111.42, 109.04, 21.02, 15.08, 13.25, 8.48, 7.97; IR (KBr) ν/cm^−1^: 2963, 1686, 1591, 1394, 1122, 898, 812, 778; HR-MS (ESI): *m*/*z* calcd for C_16_H_16_O_3_ ([M + H]^+^) 257.1178, found 257.1172.

*6-Fluoro-3,5,9-trimethyl-7*H*-furo[3,2-*g*]chromen-7-one* (**3d**): White solid; m.p.: 220.1~220.5 °C; Yield: 82.4%; ^1^H-NMR (400 MHz, CDCl_3_) δ 7.50 (d, *J* = 1.3 Hz, 1H), 7.49 (s, 1H), 2.58 (s, 3H), 2.49 (d, *J* = 2.9 Hz, 3H), 2.29 (d, *J* = 1.2 Hz, 3H); ^13^C-NMR (100 MHz, CDCl_3_) δ155.07 (d, *J* = 2.3 Hz), 146.22, 143.90, 143.09, 141.44, 131.56 (d, *J* = 12.4 Hz), 126.02, 115.77, 115.48 (d, *J* = 2.4 Hz), 111.65 (d, *J* = 6.8 Hz), 109.88, 10.59 (d, *J* = 4.0 Hz), 8.57, 7.93; HR-MS (ESI): *m*/*z* calcd for C_14_H_11_FO_3_ ([M + H]^+^) 247.0770, found 247.0765.

*6-Chloro-3,5,9-trimethyl-7H-furo[3,2-g]chromen-7-one* (**3e**): Yellow solid; m.p.: 271.9~272.1 °C; Yield: 93.4%; ^1^H-NMR (400 MHz, DMSO) δ 7.90 (s, 1H), 7.87 (s, 1H), 2.63 (s, 3H), 2.45 (s, 3H), 2.26 (s, 3H); ^13^C-NMR (100 MHz, CDCl_3_) δ 157.46, 155.59, 148.43, 147.38, 143.17, 125.85, 118.65, 115.94, 115.83, 112.07, 109.74, 16.77, 8.54, 7.93; IR (KBr) ν/cm^−1^: 3096, 2926, 1706, 1613, 1574, 1391, 1115, 882, 801, 757; HR-MS (ESI): *m*/*z* calcd for C_14_H_11_ClO_3_ ([M + H]^+^) 263.0475, found 263.0470.

*3,9-Dimethyl-5-(trifluoromethyl)-7H-furo[3,2-g]chromen-7-one* (**3f**): Yellow solid.; m.p.: 188.4~189.0 °C; Yield: 94.5%; ^1^H-NMR (400 MHz, DMSO) δ 7.97 (s, 1H), 7.65 (s, 1H), 7.01 (s, 1H), 2.50 (s, 7H), 2.26 (s, 3H); ^13^C-NMR (100 MHz, CDCl_3_) δ 159.55, 156.22, 150.10, 143.51, 142.33 (d, *J* = 32.2 Hz), 126.08, 123.24, 120.50, 113.44 (q, *J* = 5.8 Hz), 112.87 (q, *J* = 2.3 Hz), 110.59, 109.36, 8.57, 7.78; IR (KBr) ν/cm^−1^: 3130, 2931, 1730, 1595, 1389, 1272, 1137, 1074, 870, 795; HR-MS (ESI): *m*/*z* calcd for C_14_H_9_F_3_O_3_ ([M + H]^+^) 283.0582, found 283.0577.

*2,3,5,9-Tetramethyl-7*H*-furo[3,2-*g*]chromen-7-one* (**4a**): White solid; mp.: 199.9~200.3 °C; Yield: 92.0%; ^1^H-NMR (400 MHz, CDCl_3_) δ 7.37 (s, 1H), 6.22 (s, 1H), 2.55 (s, 3H), 2.49 (d, *J* = 0.6 Hz, 3H), 2.41 (s, 3H), 2.18 (s, 3H); ^13^C-NMR (100 MHz, CDCl_3_) δ 161.52, 154.32, 153.27, 152.24, 148.78, 126.63, 115.41, 112.25, 110.39, 109.76, 108.48, 19.25, 11.93, 8.36, 7.88; IR (KBr) ν/cm^−1^: 3055, 2985, 2931, 1704, 1595, 1368, 1274, 1103, 839, 758; HR-MS (ESI): *m*/*z* calcd for C_15_H_14_O_3_ ([M + H]^+^) 243.1021, found 243.1010.

*2,3,5,6,9-Pentamethyl-7H-furo[3,2-g]chromen-7-one* (**4b**): White solid; m.p.: 201.5~202.2 °C; Yield: 82.3%; ^1^H-NMR (400 MHz, CDCl_3_) δ 7.37 (s, 1H), 2.55 (s, 3H), 2.47 (s, 3H), 2.41 (s, 3H), 2.23 (s, 3H), 2.18 (s, 3H); ^13^C-NMR (100 MHz, CDCl_3_) δ 162.49, 153.46, 151.81, 147.34, 146.78, 126.39, 118.88, 116.01, 109.97, 109.74, 107.93, 15.46, 13.29, 11.91, 8.37, 7.88; IR (KBr) ν/cm^−1^: 2920, 1698, 1593, 1435, 1122, 758; HR-MS (ESI): *m*/*z* calcd for C_16_H_16_O_3_ ([M + H]^+^) 257.1178, found 257.1166.

*6-Ethyl-2,3,5,9-tetramethyl-7H-furo[3,2-g]chromen-7-one* (**4c**): White solid; m.p.: 201.7~201.8 °C; Yield: 55.2%; ^1^H-NMR (400 MHz, CDCl_3_) δ 7.38 (s, 1H), 2.72 (q, *J* = 7.5 Hz, 2H), 2.55 (s, 3H), 2.49 (s, 3H), 2.41 (s, 3H), 2.18 (s, 3H), 1.17 (t, *J* = 7.5 Hz, 3H); ^13^C-NMR (100 MHz, CDCl_3_) δ 162.24, 153.64, 151.91, 147.58, 146.47, 126.53, 125.08, 116.34, 110.26, 109.77, 108.16, 77.40, 77.08, 76.76, 20.97, 15.06, 13.26, 11.97, 8.44, 7.97; IR (KBr) ν/cm^−1^: 2924, 1701, 1686, 1574, 1402, 1117, 895, 777; HR-MS (ESI): *m*/*z* calcd for C_17_H_18_O_3_ ([M + H]^+^) 271.1334, found 271.1324.

*6-Fluoro-2,3,5,9-tetramethyl-7H-furo[3,2-g]chromen-7-one* (**4d**): White solid; m.p.: 238.2~238.4 °C; Yield: 64.1%; ^1^H-NMR (400 MHz, CDCl_3_) δ 7.32 (s, 1H), 2.55 (s, 3H), 2.46 (d, *J* = 2.9 Hz, 3H), 2.42 (s, 3H), 2.19 (d, *J* = 0.6 Hz, 3H); ^13^C-NMR (100 MHz, CDCl_3_) δ 155.73, 153.61, 152.72, 145.62, 141.23, 131.65 (d, *J* = 12.6 Hz), 127.44, 114.91, 110.32 (d, *J* = 6.7 Hz), 109.72, 108.84, 11.96, 10.50 (d, *J* = 4.0 Hz), 8.48, 7.90; IR (KBr) ν/cm^−1^: 2920, 1706, 1573, 1402, 1115, 818, 756; HR-MS (ESI): *m*/*z* calcd for C_15_H_13_FO_3_ ([M + H]^+^) 261.0927, found 261.0916.

*6-Chloro-2,3,5,9-tetramethyl-7H-furo[3,2-g]chromen-7-one* (**4e**): White solid; m.p.: 253.8~253.9 °C; Yield: 47.3%; ^1^H-NMR (400 MHz, CDCl_3_) δ 7.40 (s, 1H), 2.66 (s, 3H), 2.56 (s, 3H), 2.43 (s, 3H), 2.19 (s, 3H); ^13^C-NMR (100 MHz, CDCl_3_) δ 157.44, 154.09, 152.84, 148.43, 146.70, 127.21, 118.04, 115.16, 110.64, 109.85, 108.56, 77.40, 77.08, 76.76, 16.62, 12.00, 8.41, 7.88; IR (KBr) ν/cm^−1^: 3091, 2920, 1704, 1573, 1115, 886, 750; HR-MS (ESI): *m*/*z* calcd for C_15_H_13_ClO_3_ ([M + H]^+^) 277.0631, found 277.0622.

*2,3,9-Trimethyl-5-(trifluoromethyl)-7H-furo[3,2-g]chromen-7-one* (**4f**): Yellow solid; m.p.: 212.1~212.2 °C; Yield: 80.1%; ^1^H-NMR (400 MHz, CDCl_3_) δ 7.53 (s, 1H), 6.74 (s, 1H), 2.58 (s, 3H), 2.43 (s, 3H), 2.19 (s, 3H); ^13^C-NMR (100 MHz, CDCl_3_) δ 159.81, 154.96, 153.26, 149.75, 142.45 (d, *J* = 32.2 Hz), 127.60, 121.93 (d, *J* = 275.7 Hz), 112.99 (q, *J* = 5.9 Hz), 111.91–110.95 (m), 110.04, 109.71, 109.03, 12.02, 8.55, 7.82; IR (KBr) ν/cm^−1^: 3073, 2920, 1730, 1703, 1410, 1288, 1116, 718; HR-MS (ESI): *m*/*z* calcd for C_15_H_11_F_3_O_3_ ([M + H]^+^) 297.0739, found 297.0729.

*5,9-Dimethyl-3-phenyl-7H-furo[3,2-g]chromen-7-one* (**5a**): White solid; m.p.: 197.5~197.9 °C; Yield: 41.7%; ^1^H-NMR (400 MHz, CDCl_3_) δ 8.01 (d, *J* = 8.0 Hz, 2H), 7.65 (t, *J* = 7.4 Hz, 1H), 7.52 (t, *J* = 7.7 Hz, 2H), 7.37 (d, *J* = 8.8 Hz, 1H), 6.71 (d, *J* = 8.8 Hz, 1H), 6.15 (s, 1H), 5.41 (s, 2H), 2.39 (s, 3H), 2.38 (s, 3H); ^13^C-NMR (100 MHz, CDCl_3_) δ 161.25, 156.26, 153.10, 149.55, 142.60, 131.29, 129.22, 127.95, 127.52, 122.78, 122.50, 116.66, 113.22, 112.77, 110.12, 77.40, 77.08, 76.76, 19.38, 8.58; IR (KBr) ν/cm^−1^: 3073, 1707, 1590, 1386, 1113, 1085, 911, 754, 698; HR-MS (ESI): *m*/*z* calcd for C_19_H_14_O_3_ ([M + H]^+^) 291.1021, found 291.1013.

*5,6,9-Trimethyl-3-phenyl-7H-furo[3,2-g]chromen-7-one* (**5b**): White solid; m.p.: 191.0~191.6 °C; Yield: 70.8%; ^1^H-NMR (400 MHz, CDCl_3_) δ 7.83 (s, 2H), 7.65 (d, *J* = 7.2 Hz, 2H), 7.53 (t, *J* = 7.6 Hz, 2H), 7.43 (t, *J* = 7.4 Hz, 1H), 2.64 (s, 3H), 2.48 (s, 3H), 2.26 (s, 3H); ^13^C-NMR (100 MHz, CDCl_3_) δ 162.38, 155.46, 148.16, 146.66, 142.37, 131.52, 129.20, 127.86, 127.55, 122.59, 122.52, 120.01, 117.31, 112.32, 109.57, 15.68, 13.51, 8.57; IR (KBr) ν/cm^−1^: 2921, 1695, 1592, 1118, 757, 693; HR-MS (ESI): *m*/*z* calcd for C_20_H_16_O_3_ ([M + H]^+^) 305.1178, found 305.1169.

*6-Ethyl-5,9-dimethyl-3-phenyl-7H-furo[3,2-g]chromen-7-one* (**5c**): White solid; m.p.: 180.0~180.1 °C; Yield: 63.2%; ^1^H-NMR (400 MHz, CDCl_3_) δ 7.81 (s, 2H), 7.64 (d, *J* = 7.2 Hz, 2H), 7.52 (t, *J* = 7.6 Hz, 2H), 7.42 (t, *J* = 7.4 Hz, 1H), 2.73 (q, *J* = 7.5 Hz, 2H), 2.62 (s, 3H), 2.48 (s, 3H), 1.16 (d, *J* = 7.5 Hz, 3H); ^13^C-NMR (100 MHz, CDCl_3_) δ 161.80, 155.40, 148.14, 146.15, 142.30, 131.45, 129.17, 127.82, 127.43, 125.86, 122.43, 122.40, 117.33, 112.39, 109.40, 21.07, 15.07, 13.23, 8.50; IR (KBr) ν/cm^−1^: 3050, 1695, 1591, 1356, 1126, 1101, 766, 705; HR-MS (ESI): *m*/*z* calcd for C_21_H_18_O_3_ ([M + H]^+^) 319.1334, found 319.1329.

*6-Fluoro-5,9-dimethyl-3-phenyl-7H-furo[3,2-g]chromen-7-one* (**5d**): Yellow solid; m.p.: 200.3~200.9 °C; Yield: 37.2%; ^1^H-NMR (400 MHz, CDCl_3_) δ 7.86 (s, 1H), 7.78 (s, 1H), 7.66–7.61 (m, 2H), 7.53 (t, *J* = 7.6 Hz, 2H), 7.47–7.40 (m, 1H), 2.65 (s, 3H), 2.48 (d, *J* = 2.8 Hz, 3H); ^13^C-NMR (100 MHz, CDCl_3_) δ 155.51, 146.36, 142.84, 141.60, 131.58, 131.14, 129.26, 128.03, 127.53, 123.56, 122.39, 116.19, 112.65 (d, *J* = 7.1 Hz), 110.39, 10.64 (d, *J* = 3.9 Hz), 8.66; IR (KBr) ν/cm^−1^: 2926, 2849, 1729, 1599, 1134, 754; HR-MS (ESI): *m*/*z* calcd for C_19_H_13_FO_3_ ([M + H]^+^) 309.0927, found 309.0922.

*6-Chloro-5,9-dimethyl-3-phenyl-7H-furo[3,2-g]chromen-7-one* (**5e**): White solid; m.p.: 187.2~188.3 °C; Yield: 70.8%; ^1^H-NMR (400 MHz, CDCl_3_) δ 7.86 (d, *J* = 2.3 Hz, 2H), 7.67–7.61 (m, 2H), 7.54 (t, *J* = 7.6 Hz, 2H), 7.45 (t, *J* = 7.4 Hz, 1H), 2.67 (s, 3H), 2.65 (s, 3H); ^13^C-NMR (100 MHz, CDCl_3_) δ 157.42, 156.03, 148.47, 147.51, 142.95, 131.08, 129.30, 128.09, 127.58, 123.45, 122.58, 119.05, 116.49, 113.14, 110.30, 16.87, 8.66; IR (KBr) ν/cm^−1^: 3051, 1720, 1608, 1120, 998, 153, 692; HR-MS (ESI): *m*/*z* calcd for C_19_H_13_ClO_3_ ([M + H]^+^) 325.0631, found 325.0629.

*9-Methyl-3-phenyl-5-(trifluoromethyl)-7H-furo[3,2-g]chromen-7-one* (**5f**): Red solid; m.p.: 201.9~202.0 °C; Yield: 78.6%; ^1^H-NMR (400 MHz, CDCl_3_) δ 7.99 (s, 1H), 7.89 (s, 1H), 7.61 (d, *J* = 7.2 Hz, 2H), 7.54 (t, *J* = 7.6 Hz, 2H), 7.44 (t, *J* = 7.3 Hz, 1H), 6.79 (s, 1H), 2.66 (s, 3H); ^13^C-NMR (100 MHz, CDCl_3_) δ 159.42 (d, *J* = 0.5 Hz), 156.64, 150.16, 143.24, 130.67, 129.34, 128.19, 127.43, 123.57, 123.16, 122.64, 120.42, 114.02 (q, *J* = 2.7 Hz), 113.93–113.77 (m), 111.09, 109.99, 8.70; IR (KBr) ν/cm^−1^: 3078, 2911, 1713, 1613, 1394, 1243, 1116, 953, 854, 752; HR-MS (ESI): *m*/*z* calcd for C_19_H_11_F_3_O_3_ ([M + H]^+^) 345.0739, found 345.0735.

*2,5,9-Trimethyl-3-phenyl-7H-furo[3,2-g]chromen-7-one* (**6a**): White solid; m.p.: 261.9~261.9 °C; Yield: 89.2%; ^1^H-NMR (400 MHz, CDCl_3_) δ 7.58–7.48 (m, 5H), 7.42 (t, *J* = 6.9 Hz, 1H), 6.24 (s, 1H), 2.63 (s, 3H), 2.57 (s, 3H), 2.46 (s, 3H); ^13^C-NMR (100 MHz, CDCl_3_) δ 161.57, 154.64, 153.37, 153.06, 149.16, 132.10, 129.04, 128.88, 127.43, 125.12, 116.97, 116.22, 112.77, 111.47, 109.22, 19.37, 12.96, 8.56; IR (KBr) ν/cm^−1^: 3062, 2917, 1707, 1592, 1396, 1104, 938, 868, 754; HR-MS (ESI): *m*/*z* calcd for C_20_H_16_O_3_ ([M + H]^+^) 305.1178, found 305.1172.

*2,5,6,9-Tetramethyl-3-phenyl-7H-furo[3,2-g]chromen-7-one* (**6b**): White solid; m.p.: 216.7~218.2 °C; Yield: 78.8%; ^1^H-NMR (400 MHz, CDCl_3_) δ 7.57 (s, 1H), 7.52 (t, *J* = 7.1 Hz, 4H), 7.42 (dd, *J* = 10.9, 4.3 Hz, 1H), 2.62 (s, 3H), 2.56 (s, 3H), 2.42 (s, 3H), 2.24 (s, 3H); ^13^C-NMR (100 MHz, CDCl_3_) δ 162.38, 155.46, 148.16, 146.66, 142.37, 131.52, 129.20, 127.86, 127.55, 122.59, 122.52, 120.01, 117.31, 112.32, 109.57, 15.68, 14.6, 13.51, 8.57; IR (KBr) ν/cm^−1^: 2956, 2920, 2850, 1697, 1592, 1396, 1324, 1120, 861, 759; HR-MS (ESI): *m*/*z* calcd for C_21_H_18_O_3_ ([M + H]^+^) 319.1334, found 319.1329.

*6-Ethyl-2,5,9-trimethyl-3-phenyl-7H-furo[3,2-g]chromen-7-one* (**6c**): Yellow solid; m.p.: 167.1~167.8 °C; Yield: 75.8%; ^1^H-NMR (400 MHz, CDCl_3_) δ 7.57 (s, 1H), 7.52 (t, *J* = 7.2 Hz, 4H), 7.42 (dd, *J* = 10.9, 4.3 Hz, 1H), 2.72 (q, *J* = 7.5 Hz, 2H), 2.62 (s, 3H), 2.56 (s, 3H), 2.44 (s, 3H), 1.16 (t, *J* = 7.5 Hz, 3H); ^13^C-NMR (100 MHz, CDCl_3_) δ 162.13, 153.84, 152.72, 147.84, 146.43, 132.34, 129.00, 128.92, 127.32, 125.50, 124.88, 117.01, 111.21, 108.67, 21.03, 15.12, 13.25, 12.96, 8.55; IR (KBr) ν/cm^−1^: 2949, 1697, 1591, 1369, 1331, 1120, 940, 753, 701; HR-MS (ESI): *m*/*z* calcd for C_22_H_20_O_3_ ([M + H]^+^) 333.1491, found 333.1482.

*6-Fluoro-2,5,9-trimethyl-3-phenyl-7H-furo[3,2-g]chromen-7-one* (**6d**): White solid; m.p.: 221.1~221.4 °C; Yield: 60.3%; ^1^H-NMR (400 MHz, CDCl_3_) δ 7.53 (dd, *J* = 15.5, 4.2 Hz, 6H), 2.63 (s, 3H), 2.58 (s, 3H), 2.42 (s, 3H); ^13^C-NMR (100 MHz, CDCl_3_) δ 153.85 (d, *J* = 2.0 Hz), 153.39, 145.92, 141.44, 131.94, 131.72 (d, *J* = 13.1 Hz), 129.08, 128.87, 127.51, 125.85, 116.88, 115.66, 111.34 (d, *J* = 6.8 Hz), 109.43, 99.99, 12.97, 10.60 (d, *J* = 3.9 Hz), 8.61; IR (KBr) ν/cm^−1^: 2932, 1723, 1398, 1181, 1136, 868, 754; HR-MS (ESI): *m*/*z* calcd for C_20_H_15_FO_3_ ([M + H]^+^) 323.1083, found 323.1075.

*6-Chloro-2,5,9-trimethyl-3-phenyl-7H-furo[3,2-g]chromen-7-one* (**6e**): Yellow solid.; m.p.: 237.6~238.3 °C; Yield: 71.4%; ^1^H-NMR (400 MHz, CDCl_3_) δ 7.58 (s, 1H), 7.53 (dt, *J* = 14.5, 7.3 Hz, 4H), 7.43 (t, *J* = 7.1 Hz, 1H), 2.63 (s, 3H), 2.61 (s, 3H), 2.58 (s, 3H); ^13^C-NMR (100 MHz, CDCl_3_) δ 157.60, 154.42, 153.50, 148.65, 147.12, 131.89, 129.11, 128.90, 127.55, 125.72, 118.61, 117.03, 116.04, 111.80, 109.35, 16.85, 13.01, 8.63; IR (KBr) ν/cm^−1^: 2922, 1715, 1607, 1570, 1320, 1113, 943, 856, 756; HR-MS (ESI): *m*/*z* calcd for C_20_H_15_ClO_3_ ([M + H]^+^) 339.0788, found 339.0783.

*2,9-Dimethyl-3-phenyl-5-(trifluoromethyl)-7H-furo[3,2-g]chromen-7-one* (**6f**): Yellow solid; m.p.: 208.7~209.3 °C; Yield: 40.4%; ^1^H-NMR (400 MHz, CDCl_3_) δ 7.72 (s, 1H), 7.54 (t, *J* = 7.6 Hz, 2H), 7.48 (d, *J* = 7.6 Hz, 2H), 7.43 (t, *J* = 7.2 Hz, 1H), 6.75 (s, 1H), 2.64 (s, 3H), 2.59 (s, 3H); ^13^C-NMR (100 MHz, CDCl_3_) δ 159.60, 155.04, 153.92, 149.83, 142.37 (q, *J* = 32.5 Hz), 131.43, 128.92 (d, *J* = 37.6 Hz), 127.63, 125.86, 123.18, 120.43, 117.05, 113.32 (q, *J* = 5.8 Hz), 112.56 (d, *J* = 2.6 Hz), 110.09, 109.55, 12.99, 8.60; IR (KBr) ν/cm^−1^: 3089, 1726, 1600, 1276, 1126, 879, 758; HR-MS (ESI): *m*/*z* calcd for C_20_H_13_F_3_O_3_ ([M + H]^+^) 359.0895, found 359.0890.

### 2.2. The Crystal Structure of Compounds ***3a*** and ***6f***

The crystallographic data have been deposited with the Cambridge Crystallographic Data Centre (CCDC) as supplementary publication; number CCDC 1448022 (compound **3a**), CCDC 1448050 (compound **6f**). Copies of the data can be obtained, free of charge, on application to CCDC, 12 Union Road, Cambridge CB2 1EZ, UK (Fax: +44 1223 336033 or E-mail: deposit@ccdc.cam.ac.uk).

### 2.3. Biological Assays

All the synthesized compounds were evaluated in vitro against six plants pathogenic fungi (*Rhizoctorzia solani*, *Botrytis cinerea*, *Alternaria solani*, *Gibberella zeae*, *Cucumber anthrax*, and *Alternaria* leaf spot), which were widely found in plants, using the mycelium growth inhibitory rate methods on PDA (a kind of culture medium within potato, agar and water), with Osthole used as the positive control. The tested compounds were dissolved in dimethylformamide (DMF) to prepare the stock solution before mixing with molten agar below 55 °C. The medium containing compounds at a concentration of 100 μg/mL for the preliminary screening was poured into sterilized petri dishes. Mycelial disks (5 mm in diameter) were then inoculated in the center of the Petri dishes and incubated at 25 °C for 3 to 5 days. Each experiment was carried out in triplicates. DMF served as the negative control. The colony diameter of each strain was measured by cross bracketing method, and the inhibitory rates of the compounds were summarized in Table 1.

## 3. Results and Discussion

### 3.1. Synthetic Chemistry

In the synthesis of linear furancoumarins, furan rings were usually formed through 7-hydroxycoumarins and alkyne or α-halogenated ketone, with heavy metals often used as a catalyst. The approach leaded to environmental pollution and a higher cost. This study adopted the cheaper materials and the more simple method to generate the product. Ether derivatives were synthesized by etherification from different substituted 7-hydroxycoumarins through Williamson conditions Thereafter, cyclization of oxo ether derivatives were accomplished by heating with NaOH aqueous solution in the dark under N_2_ protection. The carbonyl substituents of α-halogenated ketone were different, so the time required for the subsequent etherified product was also different. When R_3_ position was methyl, the cyclization took a short time, usually in 2 h. When R_3_ position was phenyl, it took a longer time. This could be due to the large steric hindrance.

### 3.2. Antifungal Activity and the Structure-Activity Relationships

Data of Table 1 summarized the antifungal activities of all synthesized furancoumarin derivatives against six phytopathogenic fungi at the concentration of 100 μg/mL, respectively. Although the antifungal activity of most of the fused furancoumarin derivatives was not satisfactory, some structure-activity relationships still can be discovered. First, the synthesized compounds noticeably were more efficient to *Rhizoctonia solani* and *Botrytis cinerea* than other tested fungi, which indicated that the introduction of a fused-furan moiety to the coumarin core is important for designing coumarin based fungicides for *Rhizoctonia solani* and *Botrytis cinerea*. Compound **3a** showed a broad antifungal spectrum against all tested six phytopathogenic fungi and exhibited higher inhibitory activity (67.9%) than the control Osthole (66.1%) against *Botrytis cinerea*. Besides, compound **4b** (62.4%) showed equivalent antifungal activity with Osthole (69.5%) against *Rhizoctonia solani*. Compound **3a** bears the smallest substituents among our synthesized compounds, with only two methyl groups at the periphery of the furancoumarin core, we envisioned that the size of our other designed molecules is an important issue in developing fungicides with high activity. Second, regardless of differences at the R_3_ and R_4_ position, the high inhibitory rate of compounds **4e**, **5e** and **6e** suggest that the chlorine atom on R_1_ position was essential for the antifungal activity. Third, by comparing compounds **3** and **5**, the phenyl on R_3_ position weakened the antifungal activity of the synthesized compounds. We found that compound **3f** with a trifluoromethyl group substituted at the R_2_ position also showed very high activities against *Rhizoctonia solani* and *Botrytis cinerea*, suggesting that the introduction of a trifluoromethyl is meaningful to improve the activity of these kinds of fungicides.

## 4. Conclusions

In order to find potential activity from furanocoumarin derivatives for further structural optimization, we designed and synthesized a series of psoralen derivatives in a simple and efficient way. Most of the synthesized compounds displayed potential antifungal activity against certain phytopathogenic fungi in vitro. Some of the fused funancoumarin analogues exhibited good antifungal activity against *Botrytis cinerea* and *Rhizoctonia solani*, such as compounds **3a** (67.9%), **3b** (52.4%), **3f** (61.5%), **4e** (58.2%), **5c** (58.2%), **5e** (50.0%) and **5g** (52.9%). Furthermore, compound **4b** (62.4%) represented equivalent antifungal activity with Osthole (66.1%) against *Rhizoctonia solani*. Compound **3a** was identified as the most active and therefore the most promising candidate for further study. In addition, it is reported that 9-methoxypsoralen can be used as photo-antimicrobial against *Colletotrichum acutatum* conidia under UV light exposure without any damage on the leaves [5]. Therefore, the antifungal activities of our synthesized psoralen derivatives may be further enhanced under UV light, the detailed investigation is now under way in our lab. However, in spite of the absence of UV radiation, the inhibitory rates of some synthesized compounds are appreciable. Further structural optimization of fused furancoumarin analogues is well under way, aiming to prepare analogues with improved antifungal activity.

## Figures and Tables

**Table 1:**
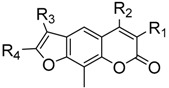
Preliminary antifungal activities of all target compounds.

Compound	R_1_, R_2_, R_3_, R_4_	Inhibitory Rate (%) ^a^					
		*Rhizoctonia solani*	*Botrytis cinerea*	*Alternaria solani*	*Gibberella zeae*	*Cucumber anthrax*	*Alternaria* leaf spot
**3a**	H, Me, Me, H	42.4	67.9	10.0 ^b^	30.7	34.9	16.2
**3b**	Me, Me, Me, H	26.3	52.4	10.0	10.0	10.0	18.2
**3c**	Et, Me, Me, H	37.5	10.0	10.0	10.0	15.5	12.5
**3d**	F, Me, Me, H	23.9	10.0	10.0	10.0	10.0	10.0
**3e**	Cl, Me, Me, H	30.2	10.0	10.0	10.0	10.0	10.0
**3f**	H, CF_3_, Me, H	56.1	61.5	10.0	10.0	10.0	10.0
**4a**	H, Me, Me, Me	37.9	40.0	10.0	10.0	11.2	13.0
**4b**	Me, Me, Me, Me	62.4	10.0	20.8	11.5	16.8	10.0
**4c**	Et, Me, Me, Me	30.2	10.0	10.0	11.2	10.0	10.0
**4d**	F, Me, Me, Me	14.5	10.0	10.0	10.0	10.0	10.0
**4e**	Cl, Me, Me, Me	41.5	58.2	10.0	17.7	12.7	16.2
**4f**	H, CF_3_, Me, Me	40.8	10.0	10.0	10.0	10.0	10.0
**5a**	H, Me, Ph, H	47.8	10.0	10.0	10.0	10.0	15.3
**5b**	Me, Me, Ph, H	37.6	38.5	10.0	10.0	15.0	20.8
**5c**	Et, Me, Ph, H	50.3	58.2	10.0	10.0	10.0	10.0
**5d**	F, Me, Ph, H	23.9	10.0	10.0	10.0	10.0	10.0
**5e**	Cl, Me, Ph, H	19.4	50.0	10.0	10.0	10.0	13.8
**5f**	H, CF_3_, Ph, H	24.3	10.0	10.0	10.0	10.0	10.0
**6a**	H, Me, Ph, Me	45.9	10.0	10.0	11.9	27.4	23.1
**6b**	Me, Me, Ph, Me	10.0	10.0	10.0	10.0	22.2	10.0
**6c**	Et, Me, Ph, Me	40.0	10.0	10.0	10.0	10.0	10.0
**6d**	F, Me, Ph, Me	10.0	10.0	10.0	10.0	10.0	10.0
**6e**	Cl, Me, Ph, Me	49.1	44.5	26.0	22.5	10.0	36.9
**6f**	H, CF_3_, Ph, Me	10.0	10.0	10.0	10.0	10.0	10.0
**Osthole**	-	69.5	66.1	29.8	66.7	92.4	50.9

^a^ Inhibitory rate values were the average values of triplicate experiments, compound concentration was 100 μg/mL; ^b^ 10.0 indicate the data below 10% inhibitory.

## Supplementary Materials

The following are available online: ^1^H-NMR and ^13^C-NMR Spectra of Products, HRMS Spectra of Target Compounds.

## References

[1] Li Z., Chen S., Zhu S., Luo J., Zhang Y., Weng Q. (2015). Synthesis and Fungicidal Activity of β-Carboline Alkaloids and Their Derivatives. Molecules.

[2] Wilson R.A., Talbot N.J. (2009). Fungal physiology—A future perspective. Microbiology.

[3] Pavela R., Vrchotová N. (2013). Insecticidal effect of furanocoumarins from fruits of *Angelica archangelica* L. against larvae *Spodoptera littoralis*. Boisd. Ind. Crops Prod..

[4] Marumoto S., Miyazawa M. (2012). Structure-activity relationships for naturally occurring coumarins as β-secretase inhibitor. Bioorg. Med. Chem..

[5] De Menezes H.D., Pereira A.C., Brancini G.T.P., de Leão H.C., Júnior N.S.M., Bachmann L. (2014). Furocoumarins and coumarins photoinactivate *Colletotrichum acutatum* and *Aspergillus nidulans* fungi under solar radiation. J. Photochem. Photobiol. B.

[6] Chauthe S.K., Mahajan S., Rachamalla M., Tikoo K., Singh I.P. (2015). Synthesis and evaluation of linear furanocoumarins as potential anti-breast and anti-prostate cancer agents. Med. Chem. Res..

[7] Hafez O.M.A., Amin K.M., Abdel-Latif N.A., Mohamed T.K., Ahmed E.Y., Maher T. (2009). Synthesis and antitumor activity of some new xanthotoxin derivatives. Eur. J. Med. Chem..

[8] Shalaby N.M.M., Abd-Alla H.I., Aly H.F., Albalawy M.A., Shaker K.H., Bouajila J. (2014). Preliminary In Vitro and In Vivo Evaluation of Antidiabetic Activity of *Ducrosia anethifolia* Boiss. and Its Linear Furanocoumarins. Biomed. Res. Int..

[9] Karamat F.I., Olry A., Doerper S., Vialart G., Ullmann P., Werck-Reichhart D. (2012). CYP98A22, a phenolic ester 30-hydroxylase specialized in the synthesis of chlorogenic acid, as a new tool for enhancing the furanocoumarin concentration in Rutagraveolens. BMC Plant Biol..

[10] Sardari S., Mori Y., Horita K., Micetich R.G., Nishibe S., Daneshtalab M. (1999). Synthesis and Antifungal Activity of Coumarins and Angular Furanocoumarins. Bioorg. Med. Chem..

[11] Verotta L., Lovagli E., Vidari G., Finzi P.V., Neri M.G., Raimondi A., Parapini S., Taramelli D., Riva A., Bombardelli E. (2004). Raimondi, 4-Alkyl- and 4-phenylcoumarins from *Mesua ferrea* as promising multidrug resistant antibacterials. Phytochemistry.

[12] Zhang M.Z., Zhang R.R., Wang J.Q. (2016). Microwave-assisted synthesis and antifungal activity of novel fused Osthole derivatives. Eur. J. Med. Chem..

[13] Zhang R.R., Xu Z.C. (2015). Microwave-Assisted Synthesis and Antifungal Activities of Polysubstituted Furo[3,2-c]chromen-4-ones and 7,8,9,10-Tetrahydro-6*H*-benzofuro[3,2-*c*]chromen-6-ones. Synth. Commun..

[14] Shen Q., Peng Q., Shao J., Liu X., Huang Z., Pu X. (2005). Synthesis and biological evaluation of functionalized coumarins as acetylcholinesterase inhibitors. Eur. J. Med. Chem..

[15] Finn G.J., Creaven B., Egan D.A. (2001). Study of the in vitro cytotoxic potential of natural and synthetic coumarin derivatives using human normal and neoplastic skin cell lines. Melanoma Res..

[16] El-Gogary S., Hashem N., Khodeir M.N. (2015). Synthesis and photooxygenation of angular furocoumarins: Isopsedopsoralen and allopsoralen. Res. Chem. Intermed..

[17] Medina F.G., Marrero J.G., Macías-Alonso M. (2015). Coumarin heterocyclic derivatives: Chemical synthesis and biological activity. Nat. Prod. Rep..

[18] Zhang M.Z., Zhang R.R., Yin W.Z., Yu X., Zhang Y.L., Liu P., Gu Y.C., Zhang W.H. (2016). Microwave-assisted Synthesis and antifungal activity of coumarin[8,7-*e*][1,3]oxazine derivatives. Mol. Divers..

[19] Zhang M.Z., Zhang Y., Wang J.Q., Zhang W.H. (2016). Design, Synthesis and Antifungal Activity of Coumarin Ring-Opening Derivatives. Molecules.

[20] Zhang R.R., Liu J., Zhang Y., Hou M.Q., Zhang M.Z., Zhou F., Zhang W.H. (2016). Microwave-assisted synthesis and antifungal activity of novel coumarin derivatives: Pyrano[3,2-*c*]chromene-2,5-diones. Eur. J. Med. Chem..

[21] Podgoršek A., Stavber S., Zupan M., Iskra J. (2007). Bromination of ketones with H_2_O_2_-HBr “on water”. Green Chem..

[22] Jiang Q., Sheng W., Guo C. (2013). Synthesis of phenacyl bromides via K_2_S_2_O_8_-mediated tandem hydroxybromination and oxidation of styrenes in water. Green Chem..

[23] Zhang W.H., Jiang M.G. (2010). Synthesis and antifungal activity of umbelliferone analogues. Chin. J. Org. Chem..

