# Supplementary material for: Design, Synthesis and Antifungal Activity of Psoralen Derivatives
Source: Molecules. 2017 Oct 9;22(10):1672. doi: 10.3390/molecules22101672 (PMC6151755; doi:10.3390/molecules22101672)
Supplement: Supplementary file 1 [file molecules-22-01672-s001.pdf]

Supporting Information For：

Design, synthesis and antifungal activity of psoralen derivatives

Xiang Yu, Ya Wen, Chao-Gen Liang, Jia Liu, Yu-Bin Ding, Wei-Hua Zhang*

Jiangsu Key Laboratory of Pesticide Science, Department of Chemistry, College of Sciences, Nanjing Agricultural University, Nanjing 210095, P.R. China

Table of Contents for Supporting Information

1. ^{1}HNMR and ^{13}CNMR Spectra of Products··································S2-S25

2. HRMS Spectra of Target Compounds·······································S26-S37

1. ^{1}HNMR and ^{13}CNMR Spectra of Products

^{1}H-NMR

3a

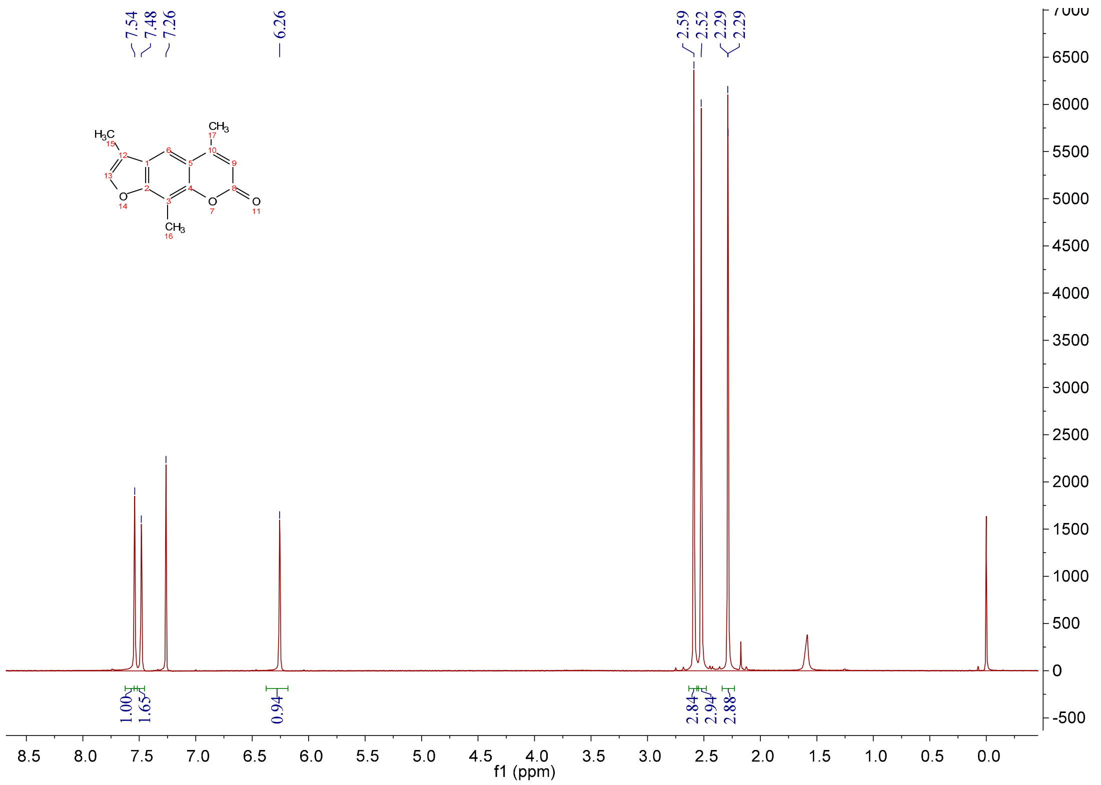

3b

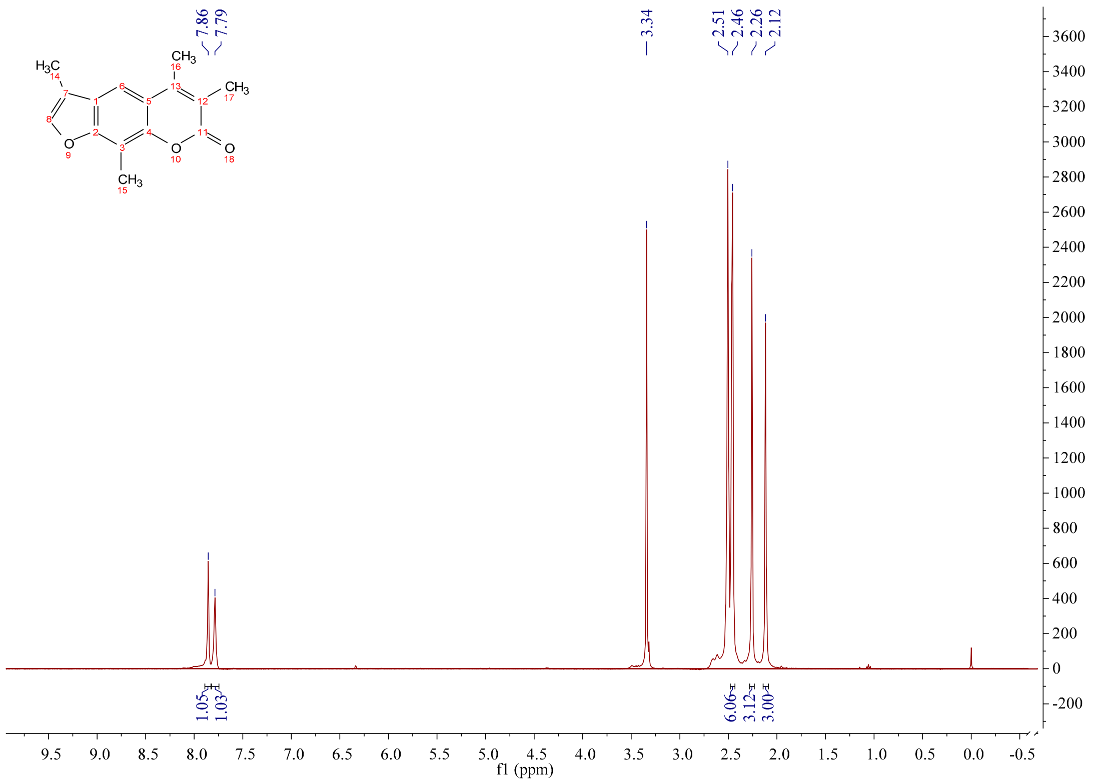

3c

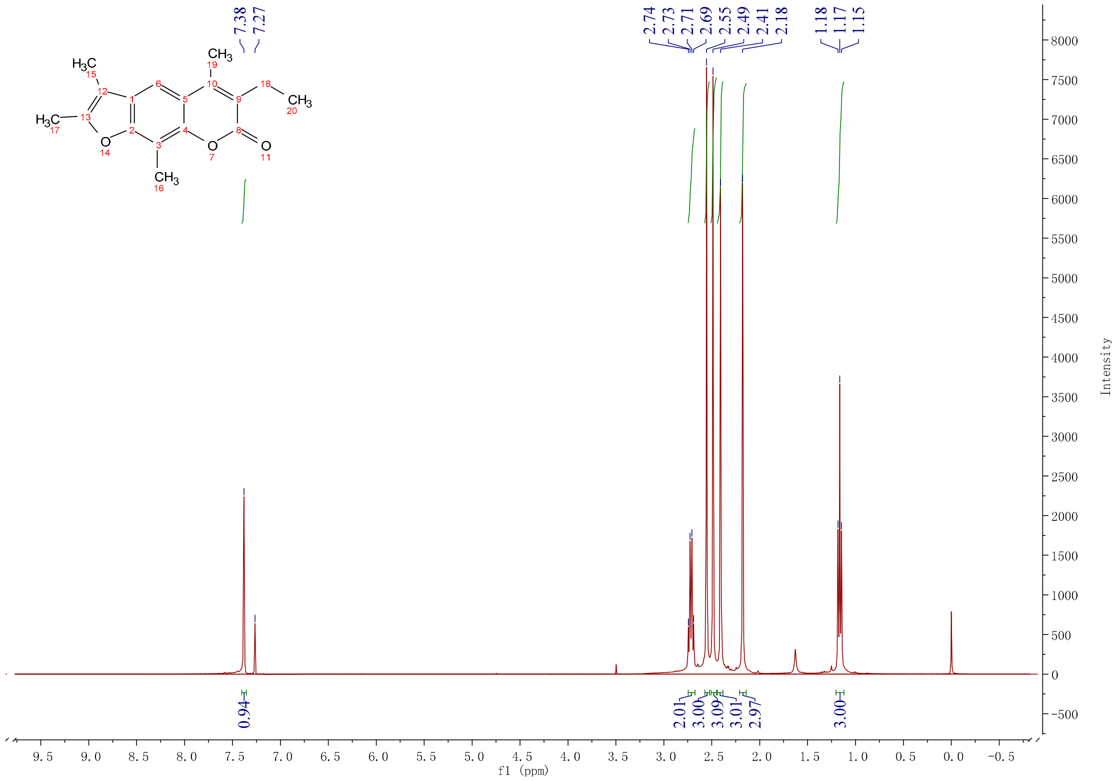

3d

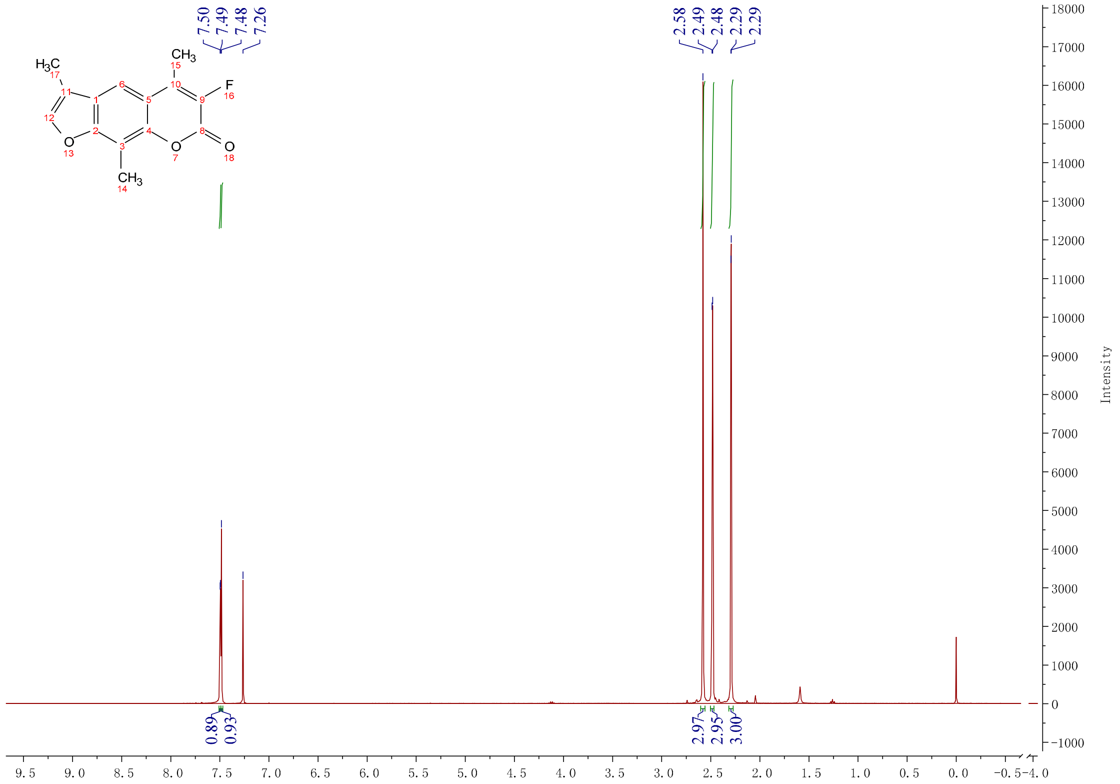

3e

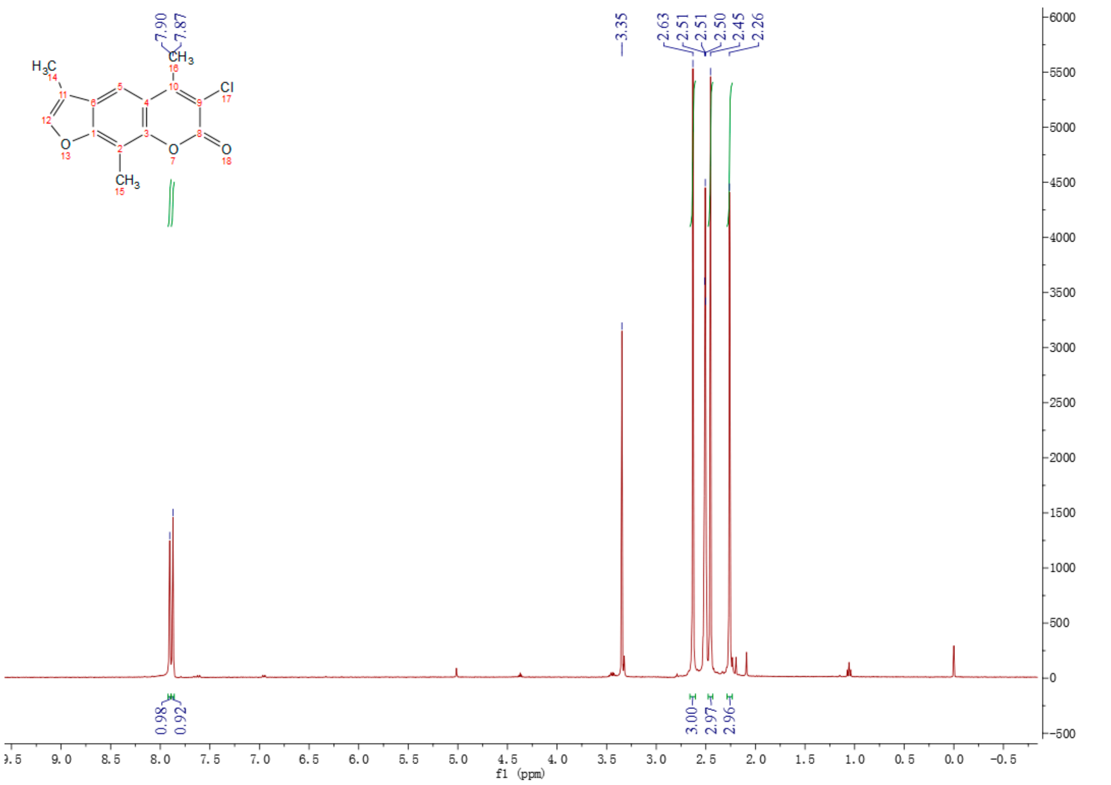

3f

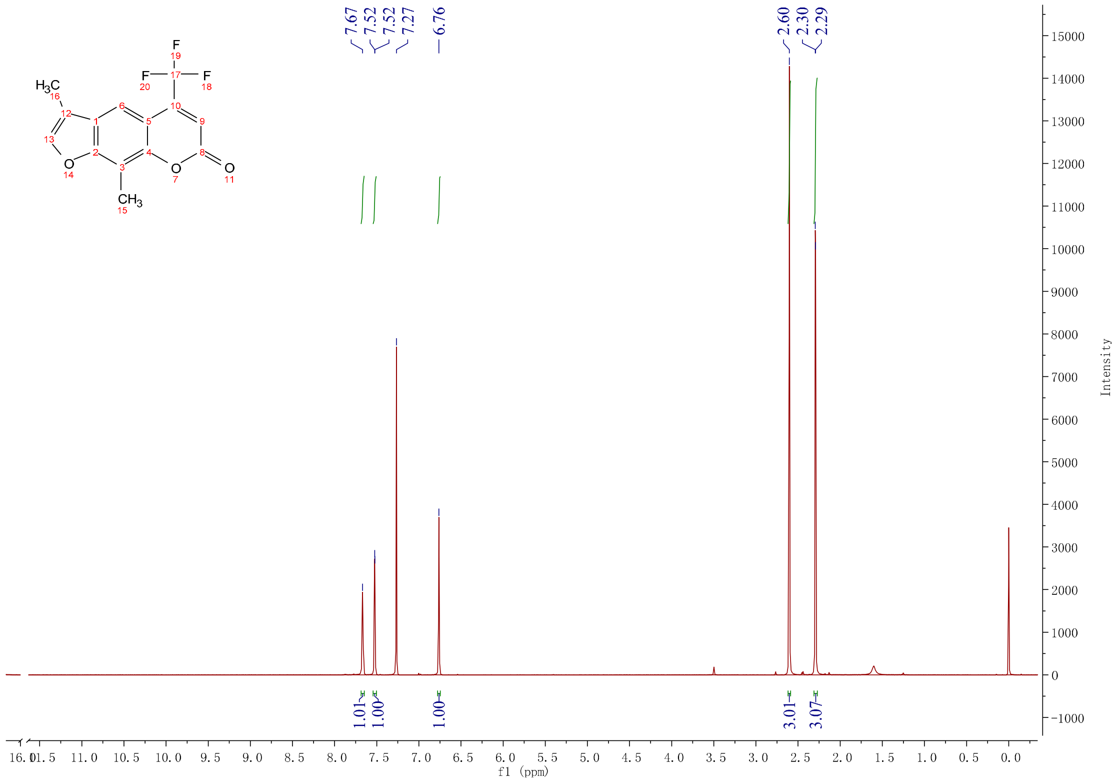

4a

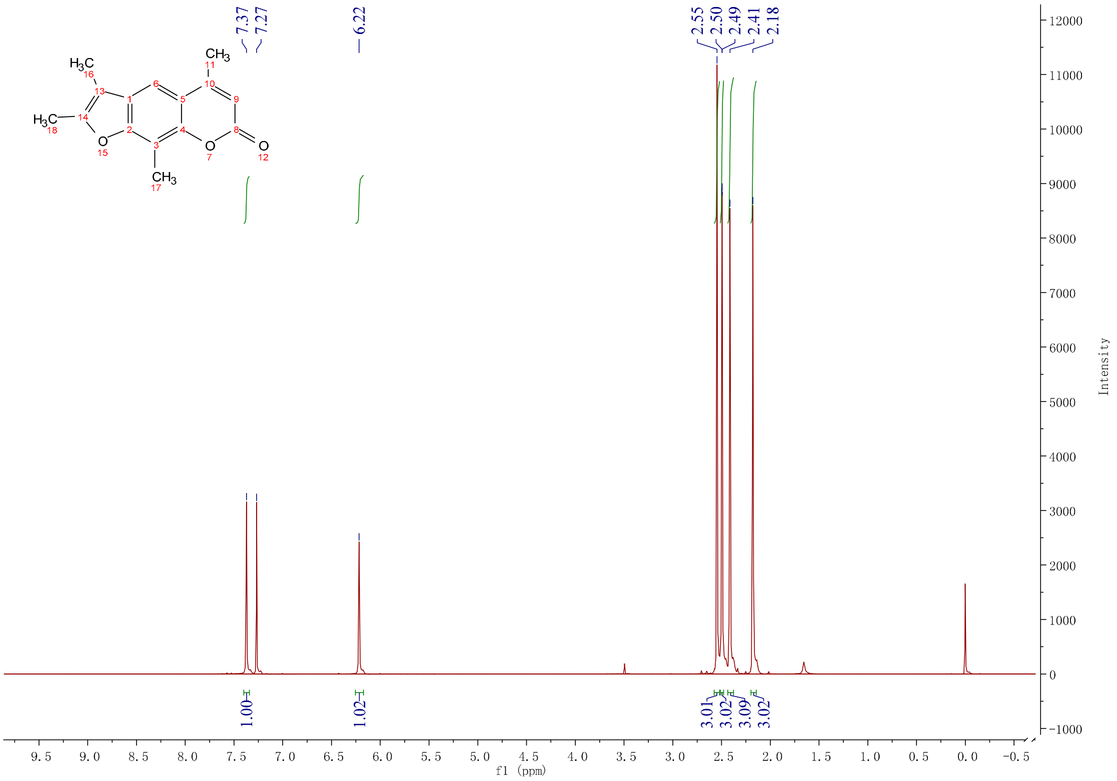
7.37
7.27
6.22
2.55
2.50
2.49
2.41
2.18
1.00
1.02
3.01
3.02
3.09
3.02
f1 (ppm)
Intensity

4b

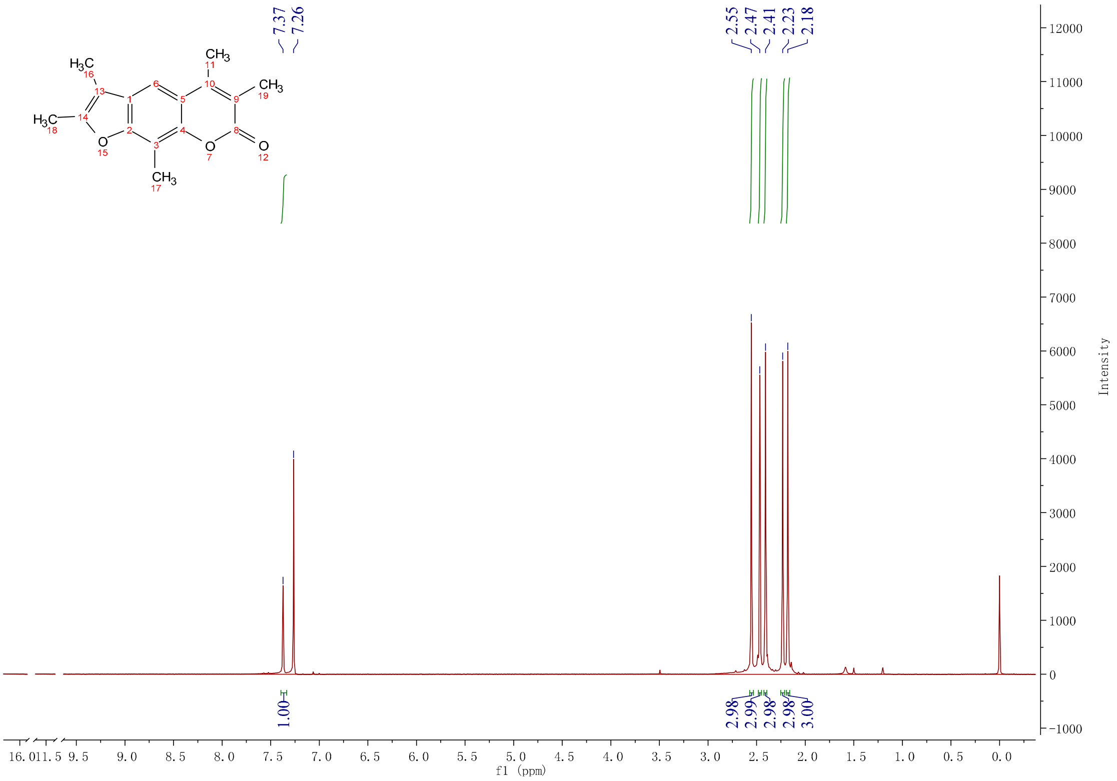
7.37
7.26
2.55
2.47
2.41
2.23
2.18
1.00
2.98
2.99
2.98
2.98
3.00
f1 (ppm)
Intensity

4c

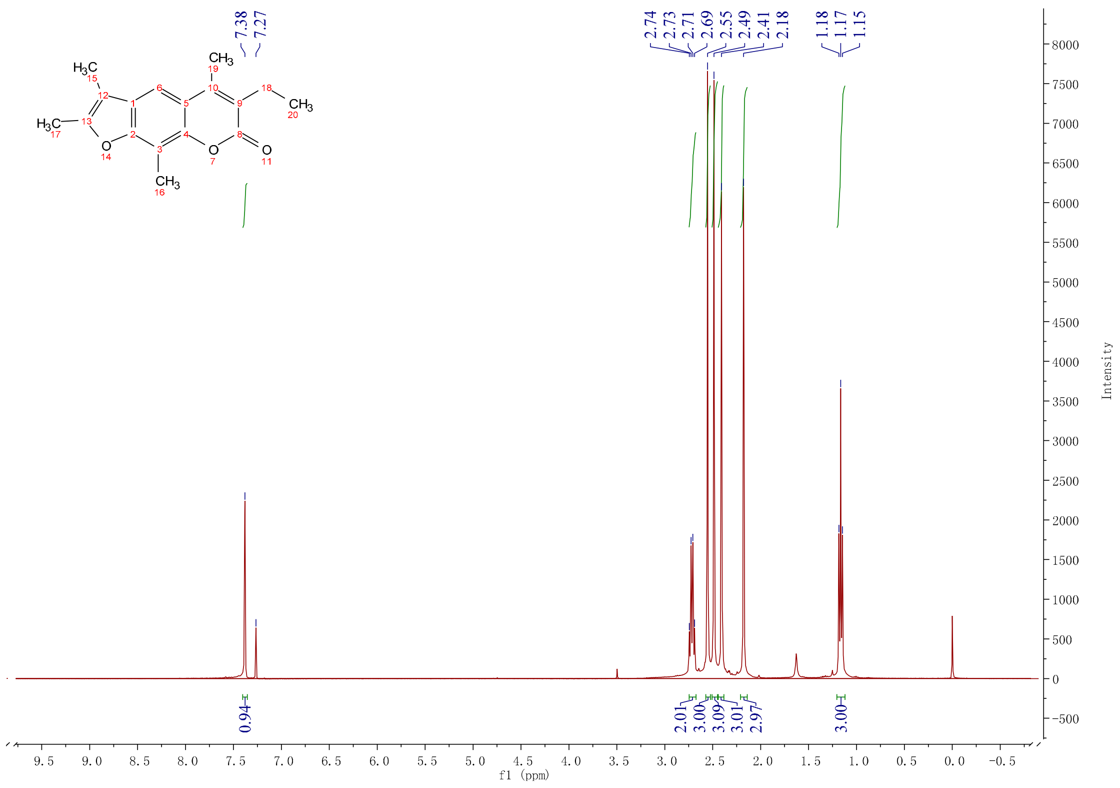

4d

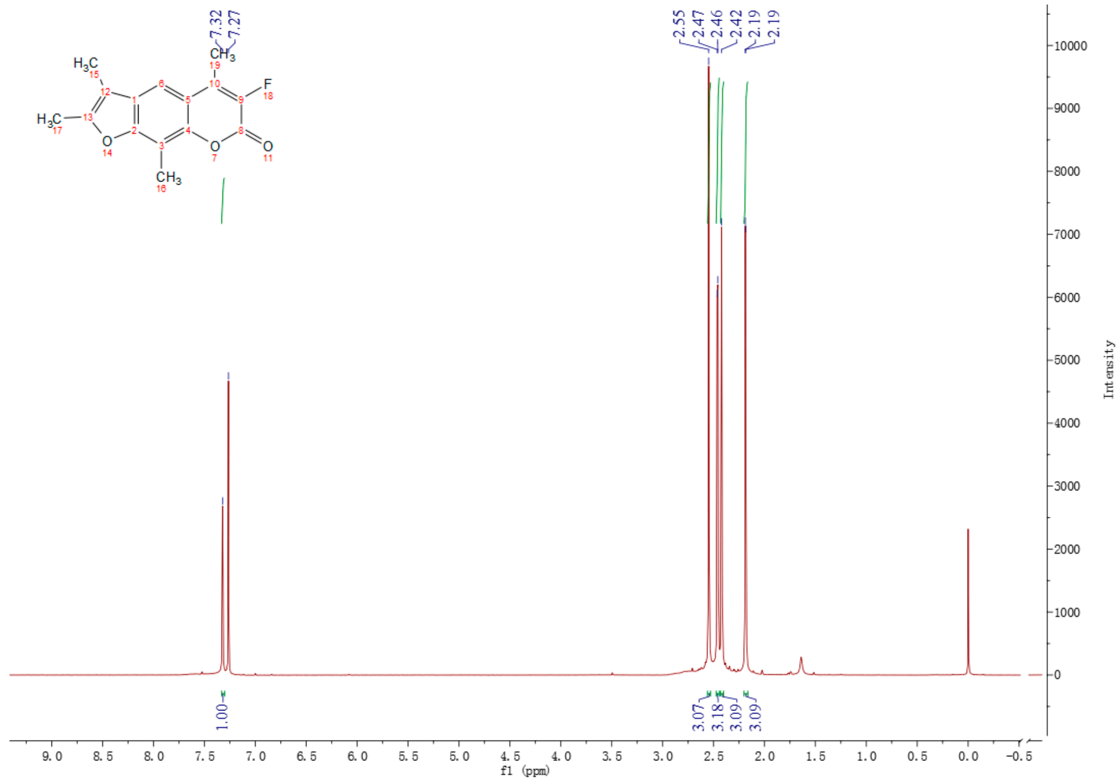

4e

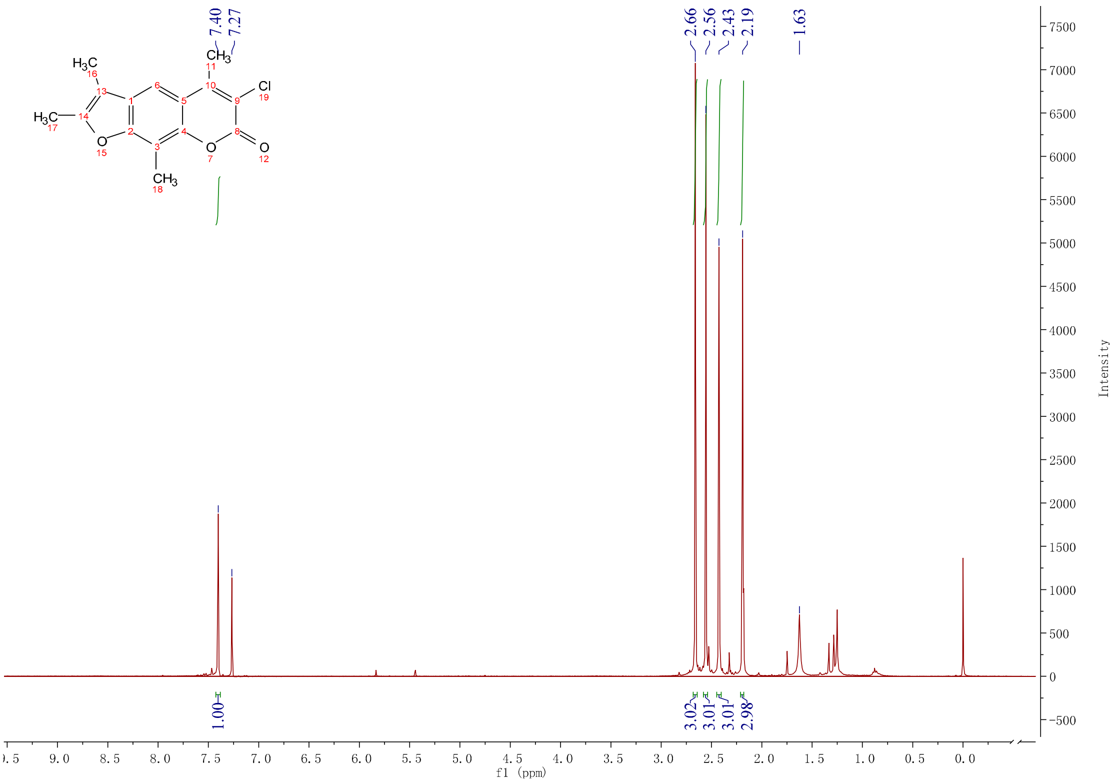
7.40
7.27
2.66
2.56
2.43
2.19
1.63
1.00
3.02
3.01
3.01
2.98
f1 (ppm)
Intensity

4f

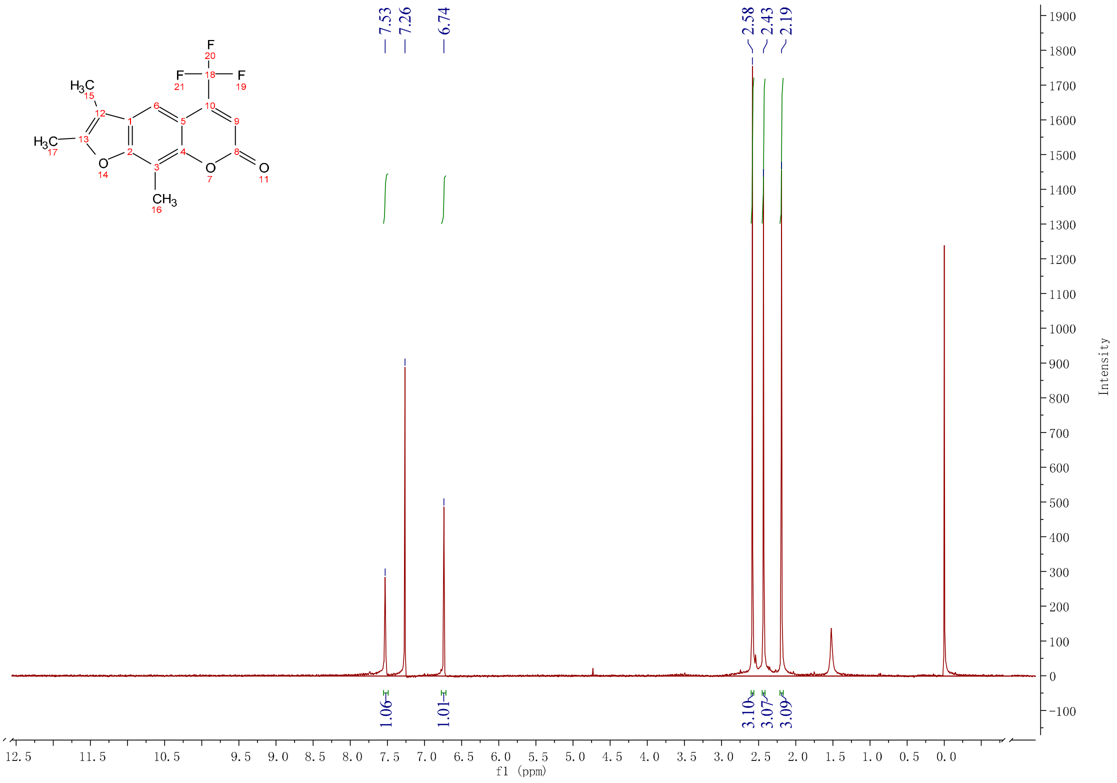
7.53
7.26
6.74
2.58
2.43
2.19
1.06
1.01
3.10
3.07
3.09
f1 (ppm)
Intensity

5a

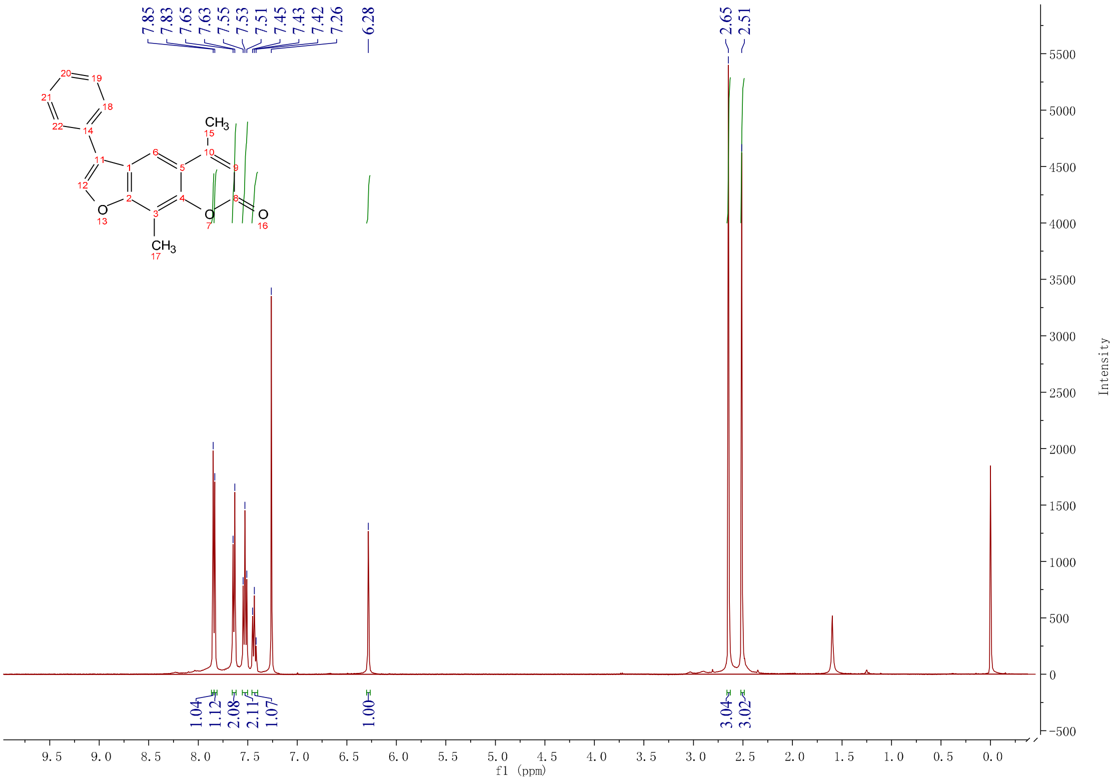

5b

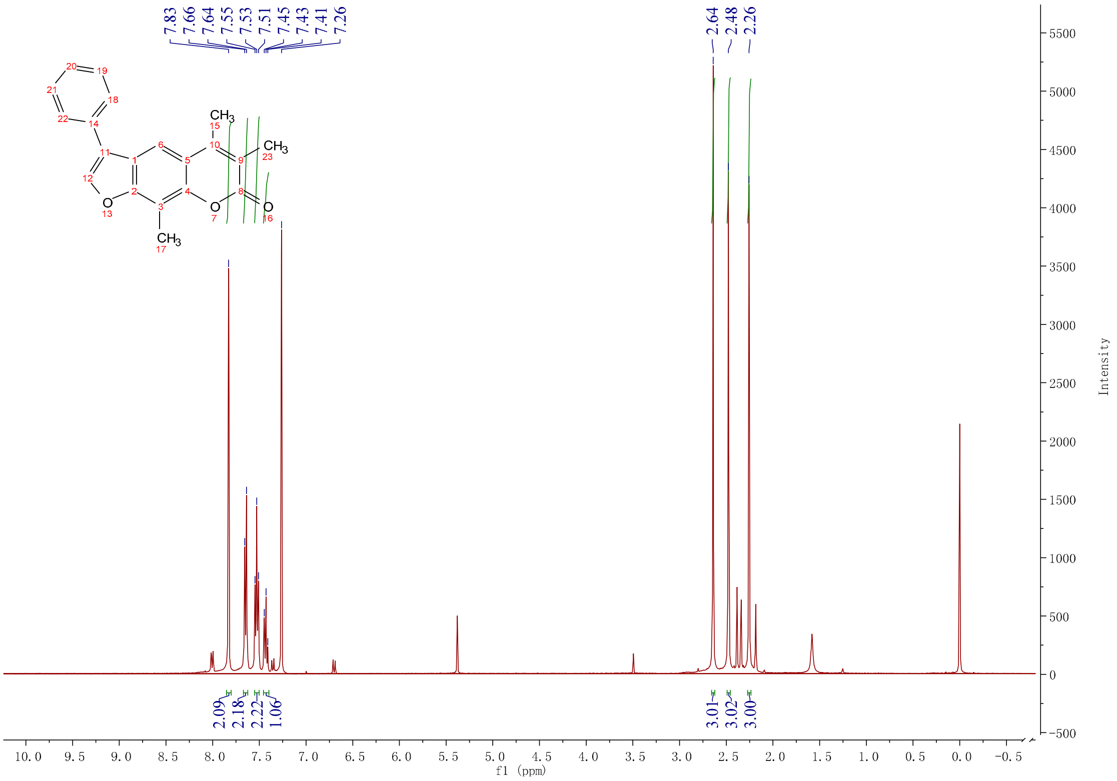

5c

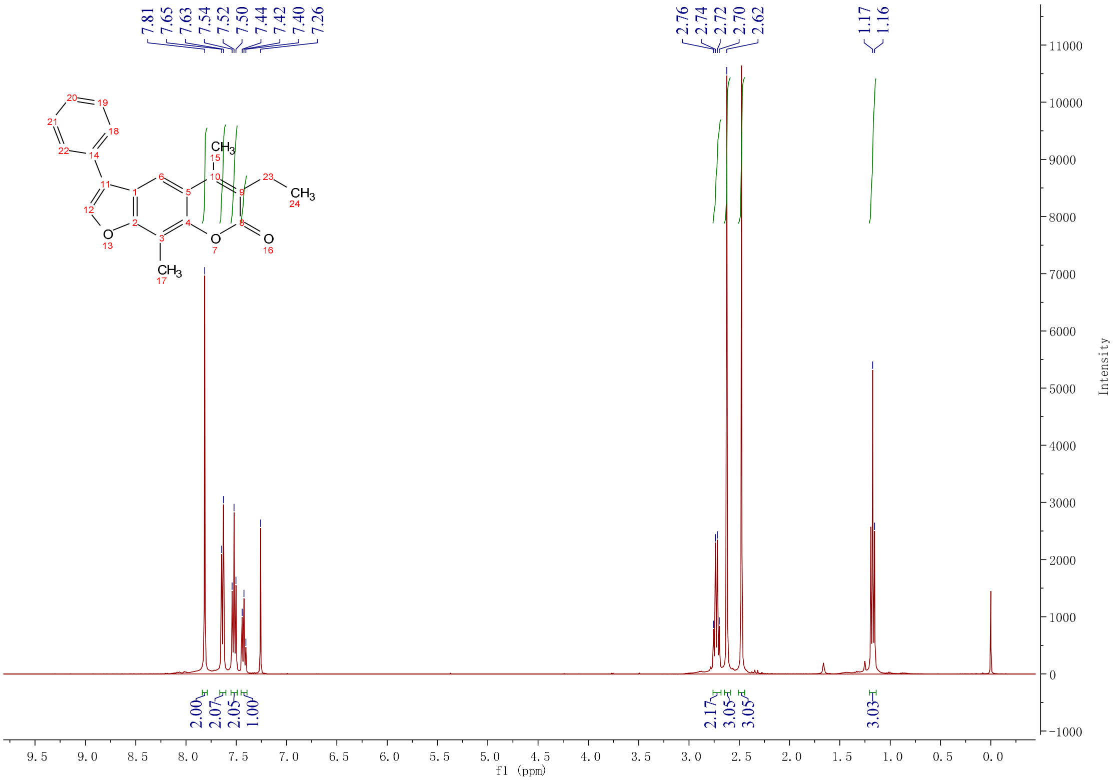

5d

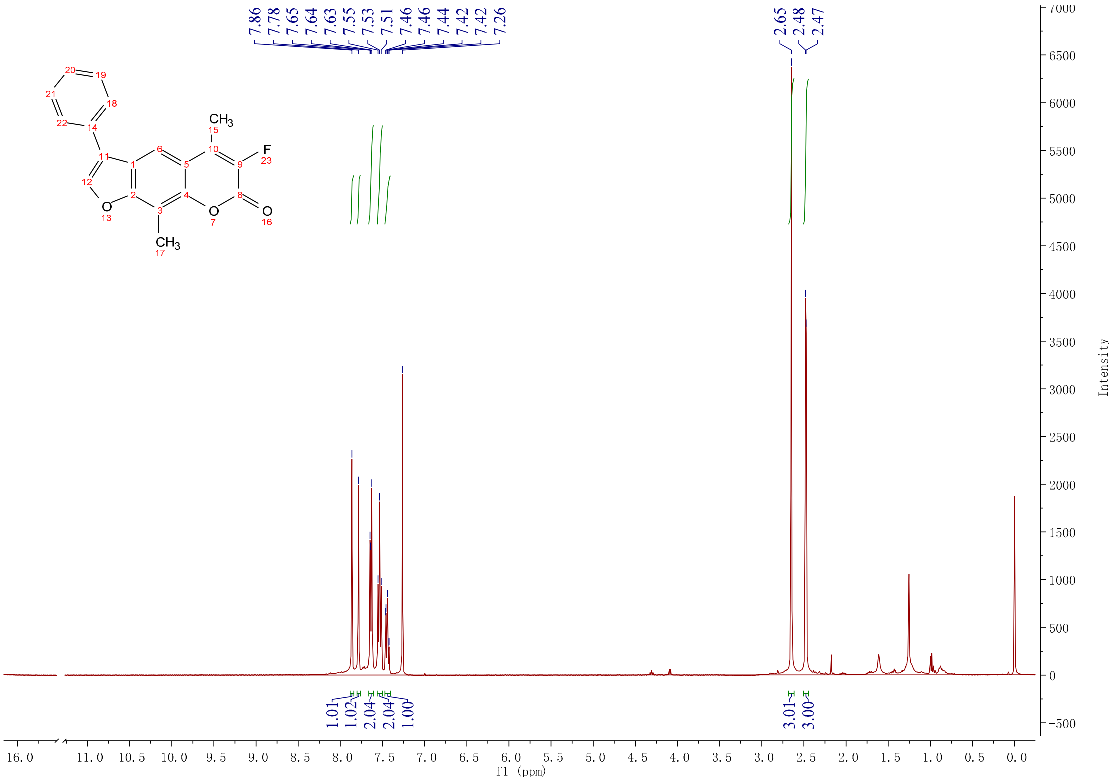

5e

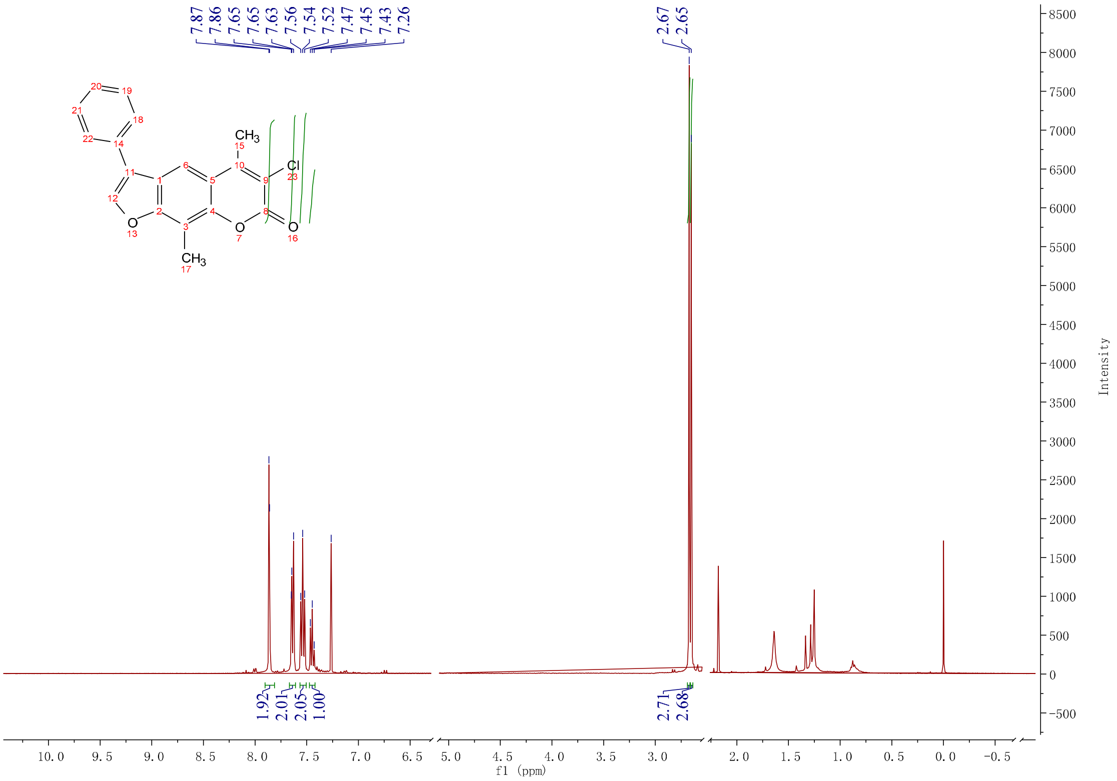

7.87
7.86
7.65
7.65
7.63
7.56
7.54
7.52
7.47
7.45
7.43
7.26
2.67
2.65
1.92
2.01
2.05
1.00
2.71
2.68
f1 (ppm)
Intensity

5f

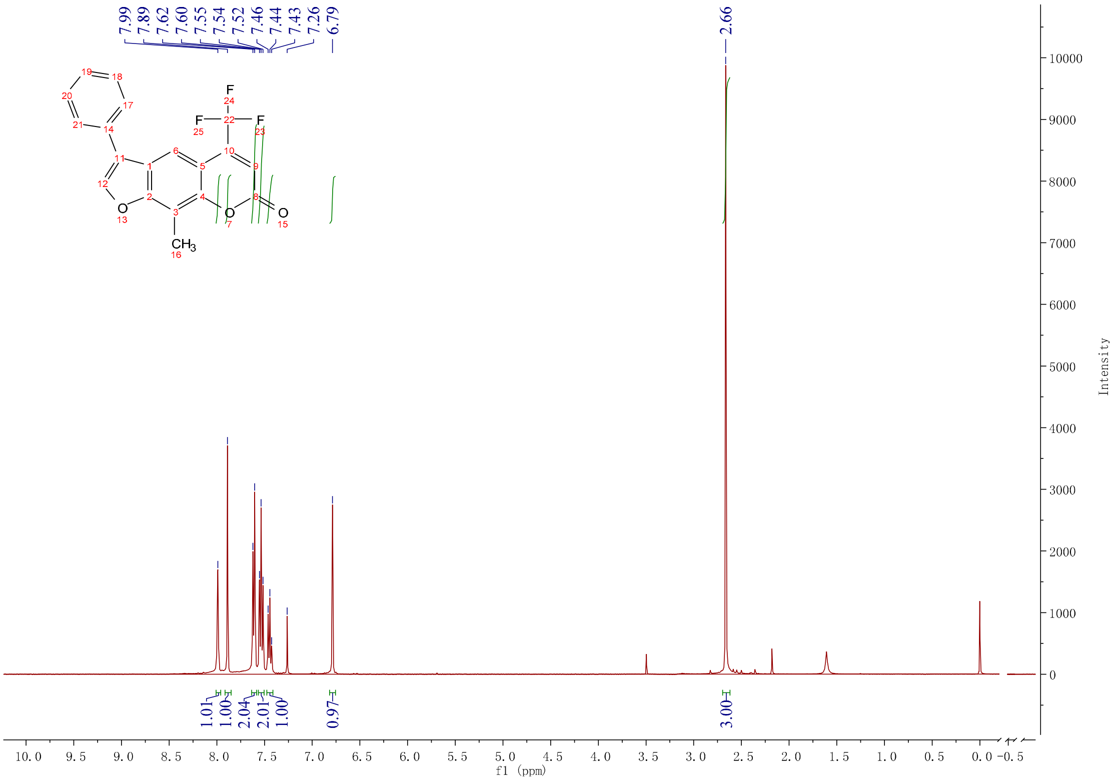

7.99
7.89
7.62
7.60
7.55
7.54
7.52
7.46
7.44
7.43
7.26
6.79
2.66
1.01
1.00
2.04
2.01
1.00
0.97
3.00
f1 (ppm)
Intensity

6a

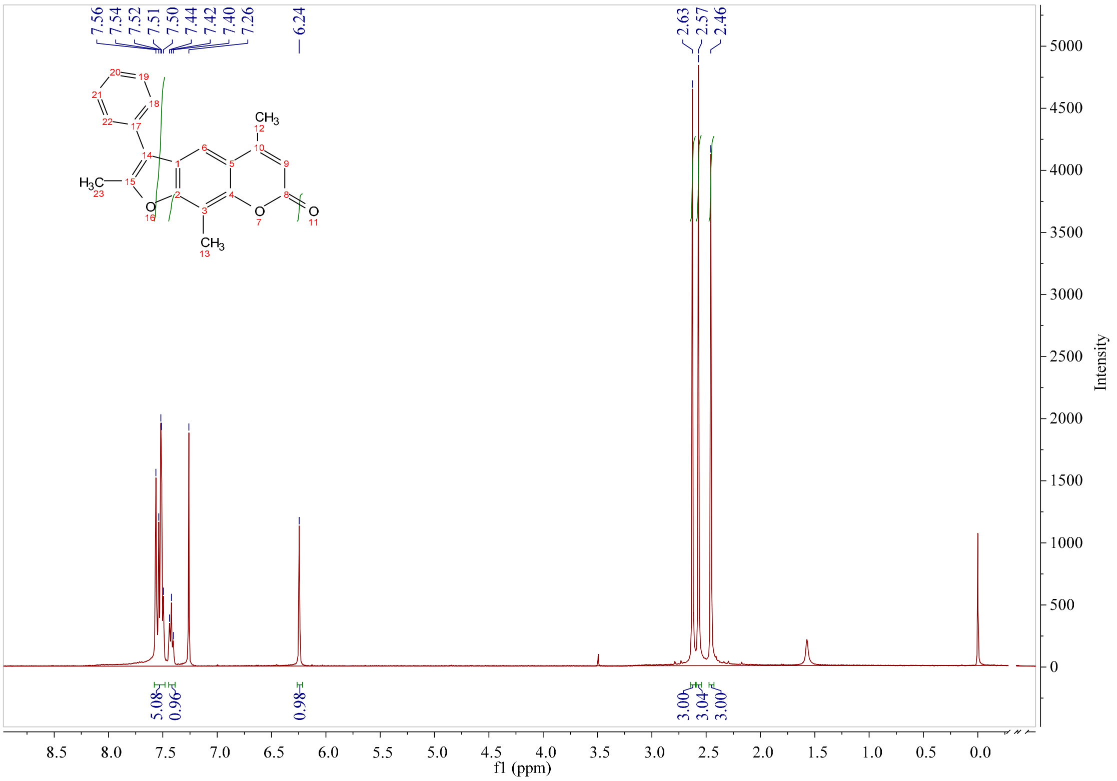
7.56
7.54
7.52
7.51
7.50
7.44
7.42
7.40
7.26
6.24
2.63
2.57
2.46
5.08
0.96
0.98
3.00
3.04
3.00
f1 (ppm)
Intensity

6b

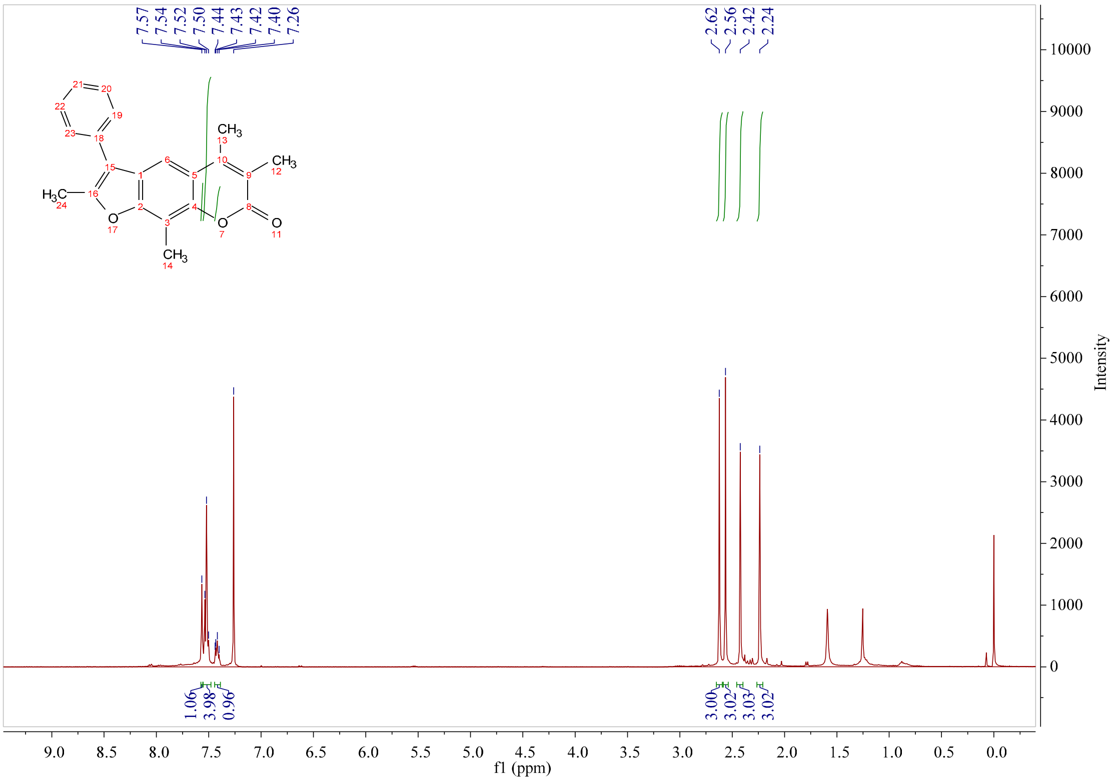
7.57
7.54
7.52
7.50
7.44
7.43
7.42
7.40
7.26
2.62
2.56
2.42
2.24
1.06
3.98
0.96
3.00
3.02
3.03
3.02
f1 (ppm)
Intensity

6c

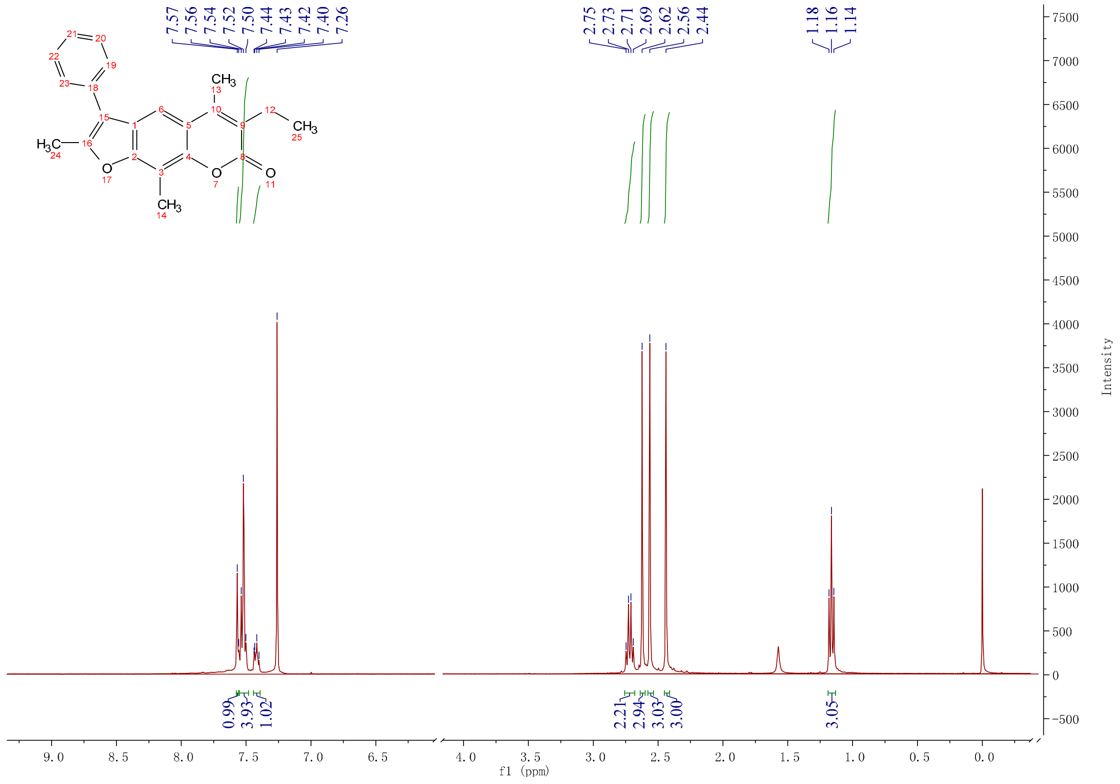

6d

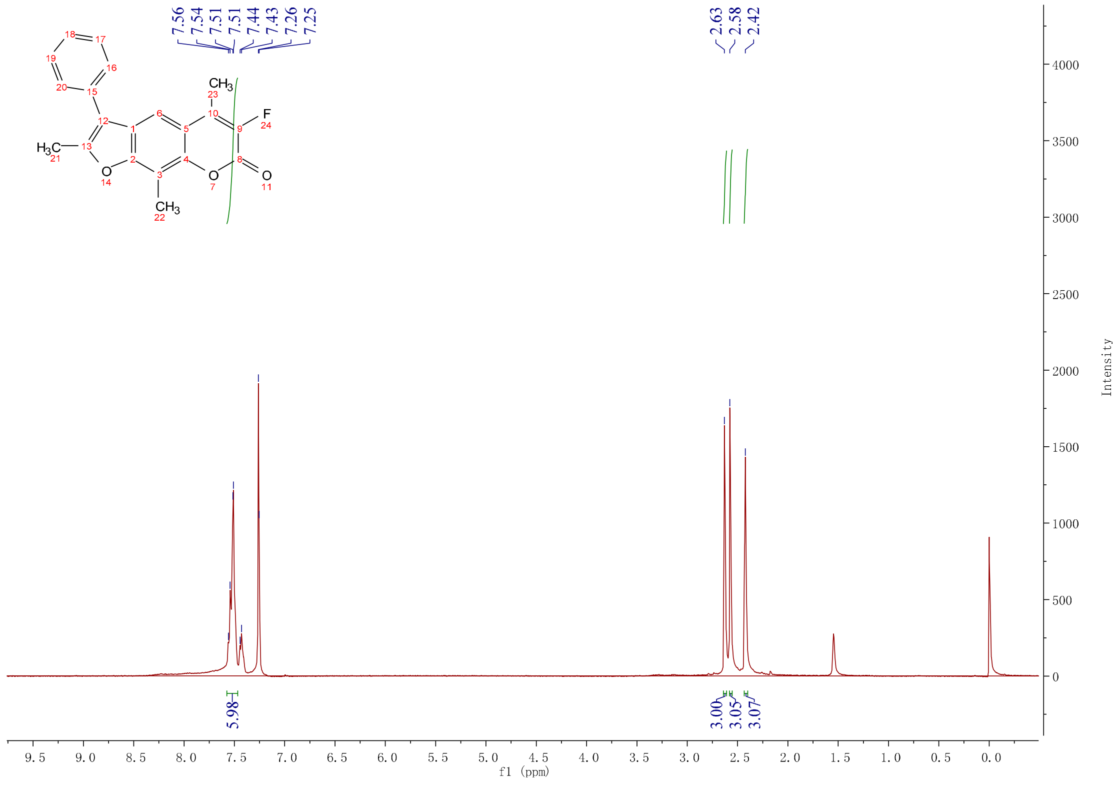

6e

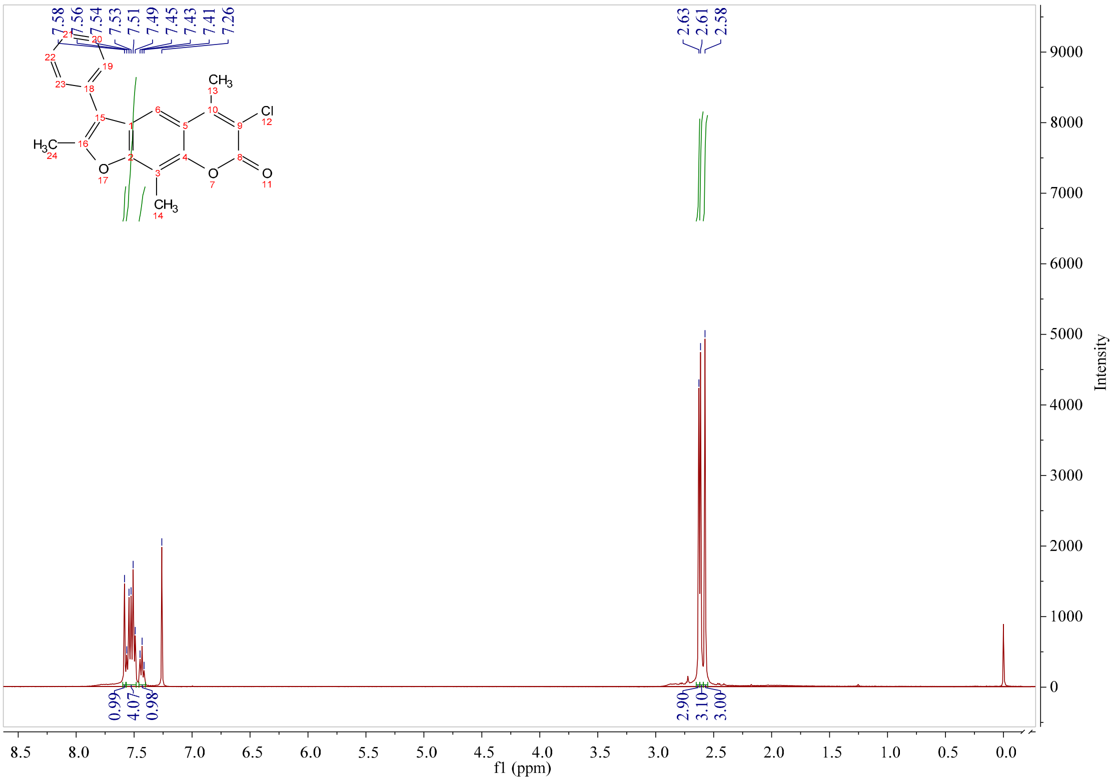
7.58
7.56
7.54
7.53
7.51
7.49
7.45
7.43
7.41
7.26
2.63
2.61
2.58
0.99
4.07
0.98
2.90
3.10
3.00
f1 (ppm)
Intensity

6f

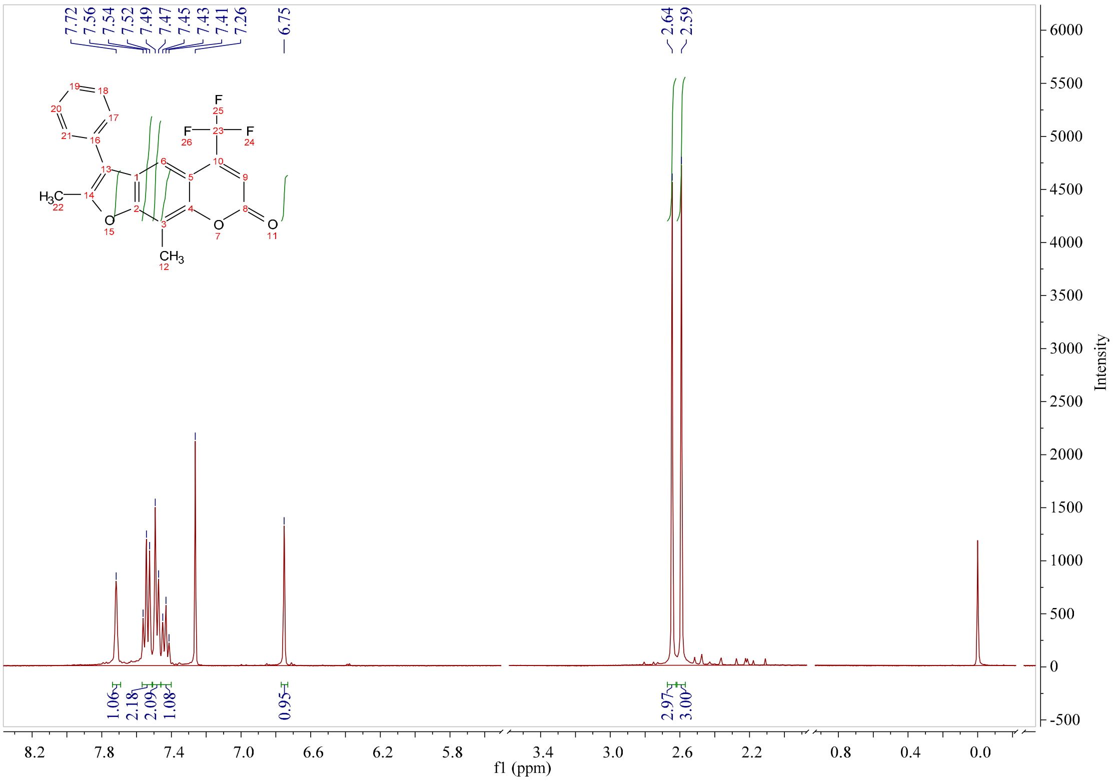
7.72
7.56
7.54
7.52
7.49
7.47
7.45
7.43
7.41
7.26
6.75
2.64
2.59
1.06
2.18
2.09
1.08
0.95
2.97
3.00
f1 (ppm)
Intensity

^{13}C-NMR

3a

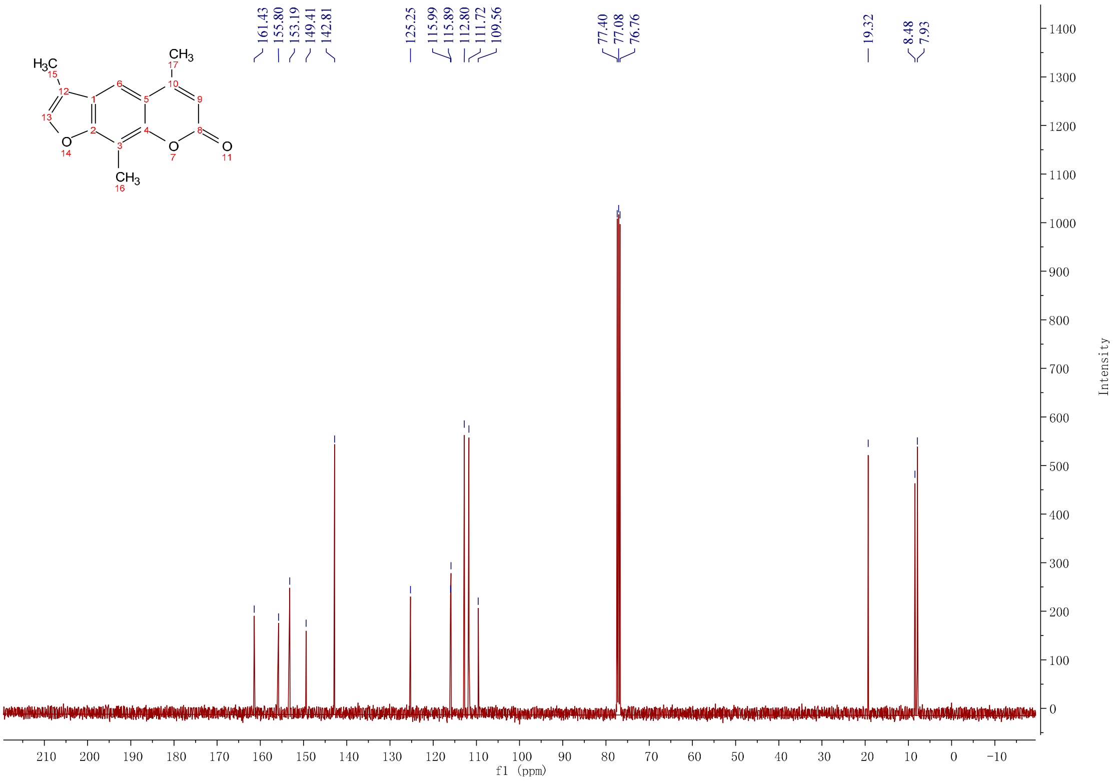
161.43
155.80
153.19
149.41
142.81
125.25
115.99
115.89
112.80
111.72
109.56
77.40
77.08
76.76
19.32
8.48
7.93
Intensity
f1 (ppm)

3b

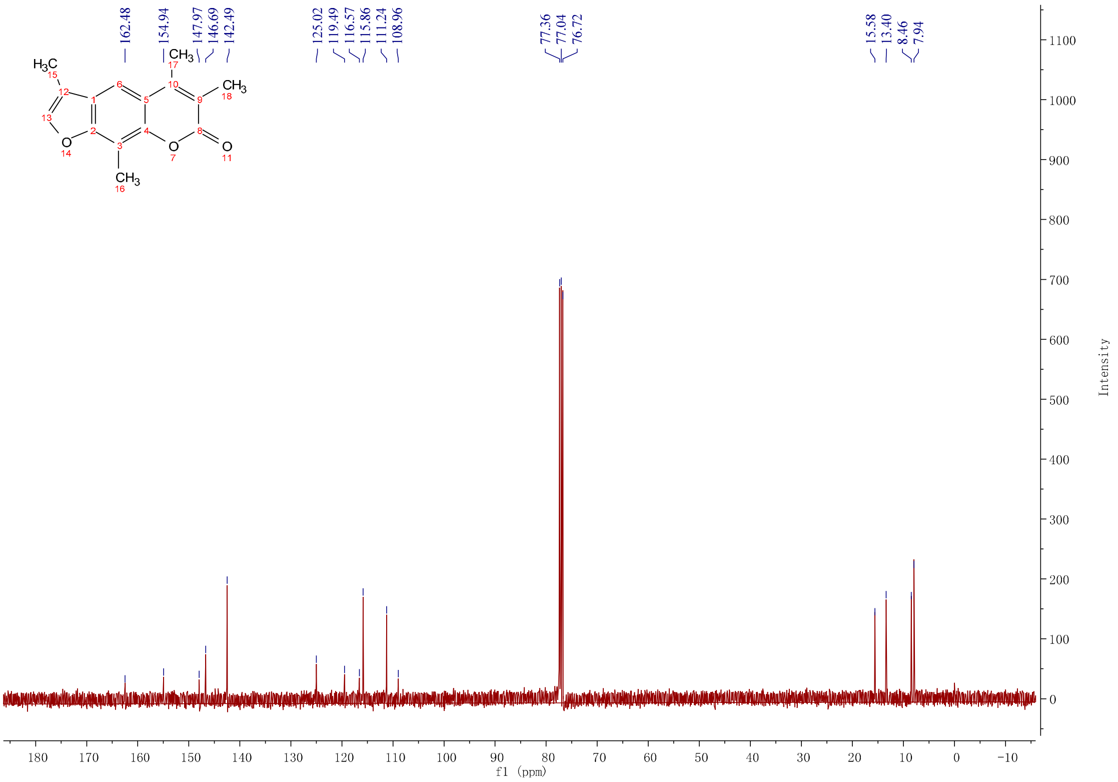
162.48
154.94
147.97
146.69
142.49
125.02
119.49
116.57
115.86
111.24
108.96
77.36
77.04
76.72
15.58
13.40
8.46
7.94
Intensity
f1 (ppm)

3c

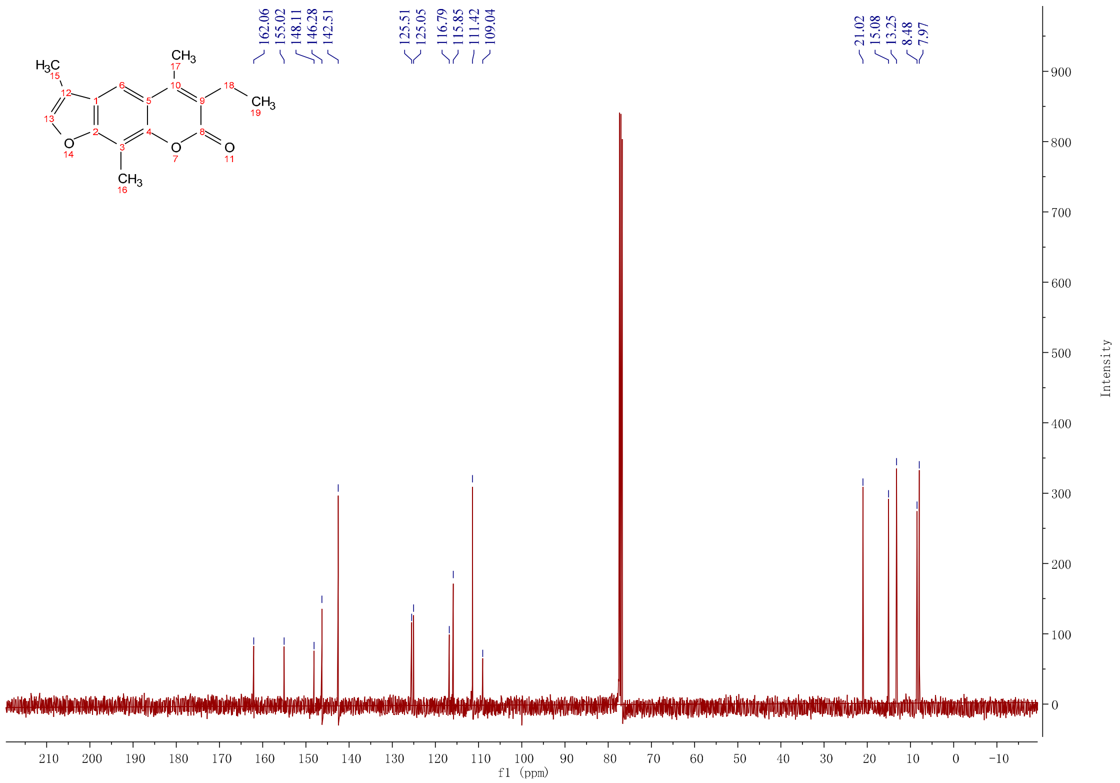

162.06
155.02
148.11
146.28
142.51
125.51
125.05
116.79
115.85
111.42
109.04
21.02
15.08
13.25
8.48
7.97
Intensity
f1 (ppm)

3d

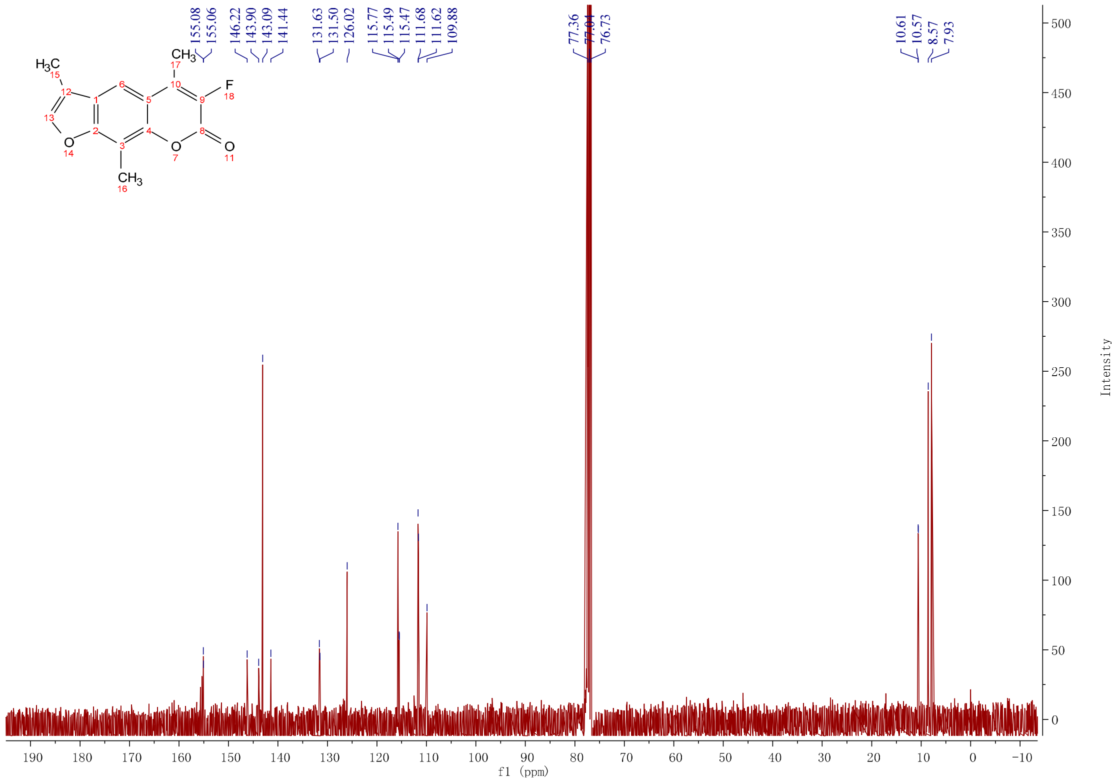

155.08
155.06
146.22
143.90
143.09
141.44
131.63
131.50
126.02
115.77
115.49
115.47
111.68
111.62
109.88
77.36
76.73
10.61
10.57
8.57
7.93
Intensity
f1 (ppm)

3e

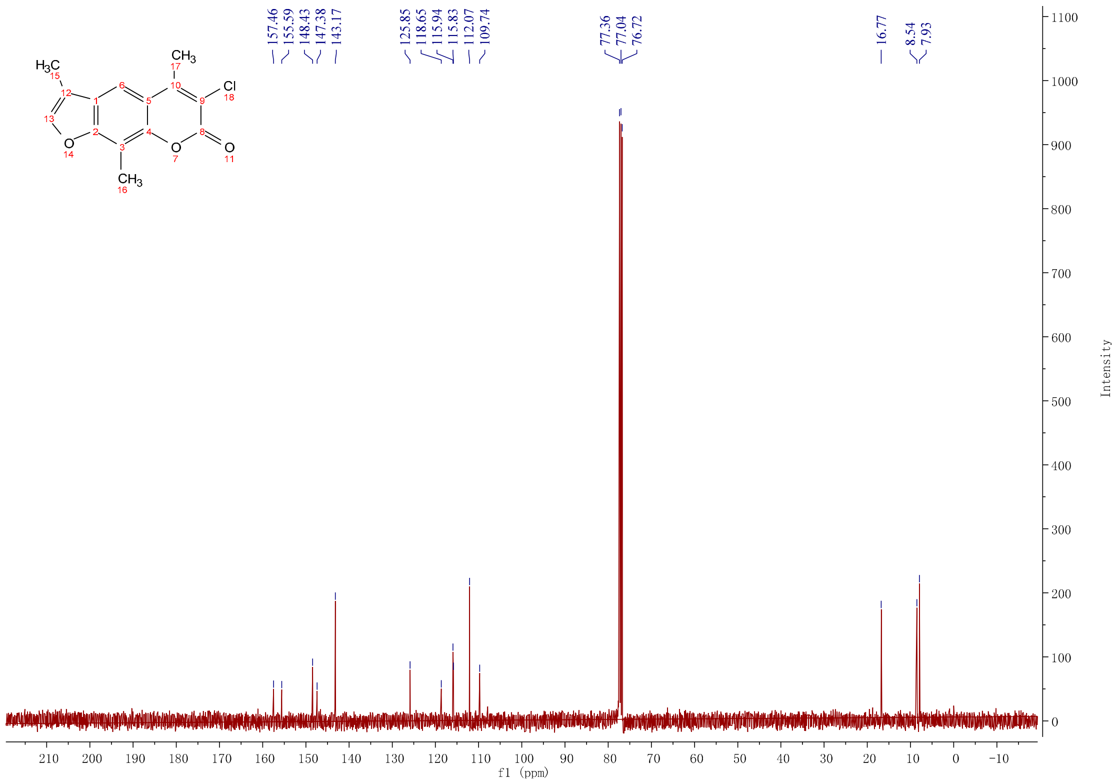

3f

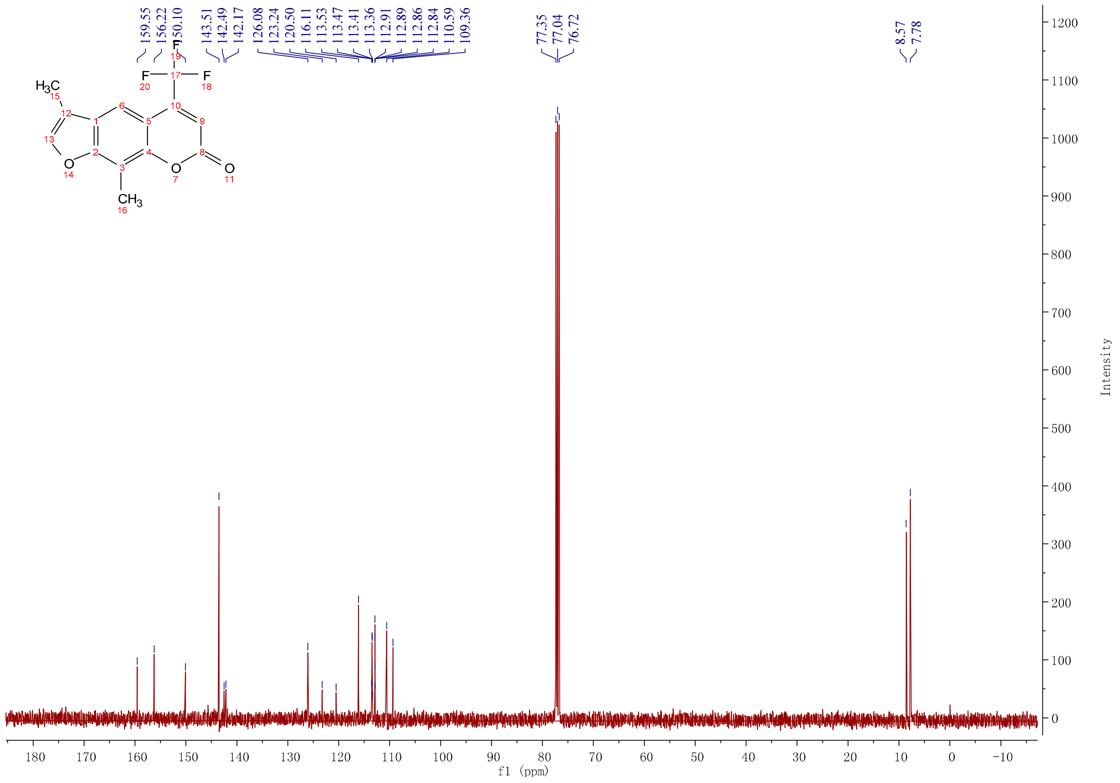

4a

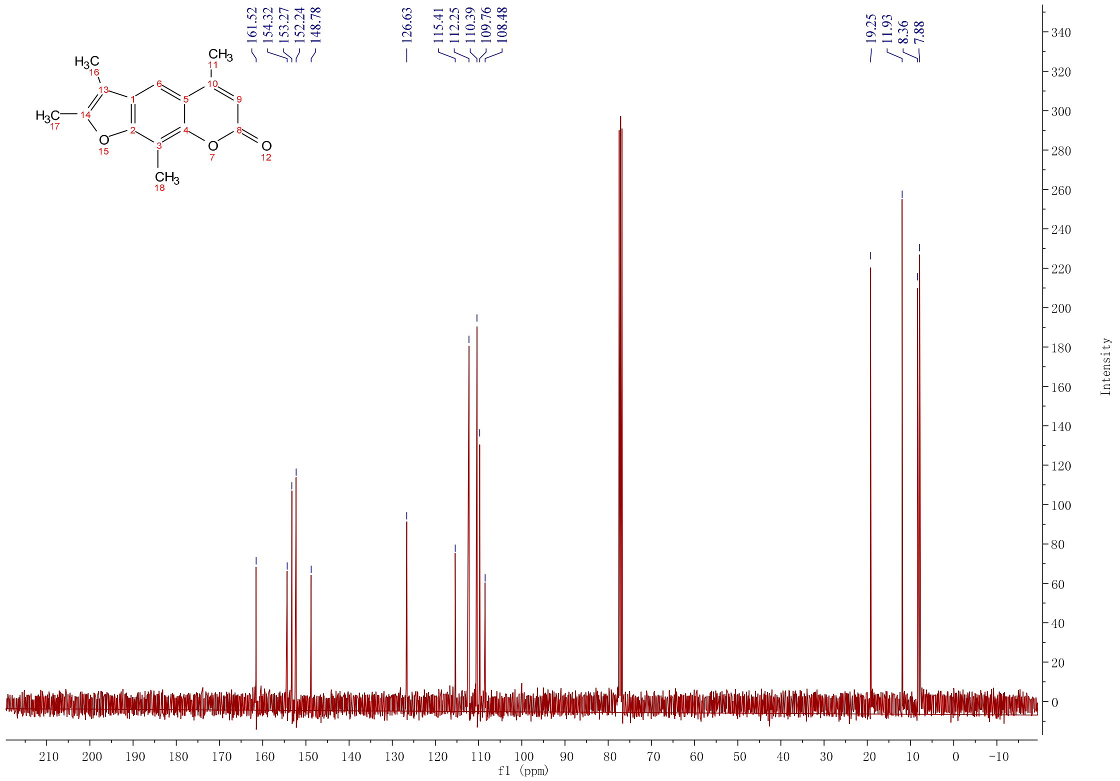
161.52
154.32
153.27
152.24
148.78
126.63
115.41
112.25
110.39
109.76
108.48
19.25
11.93
8.36
7.88
Intensity
f1 (ppm)

4b

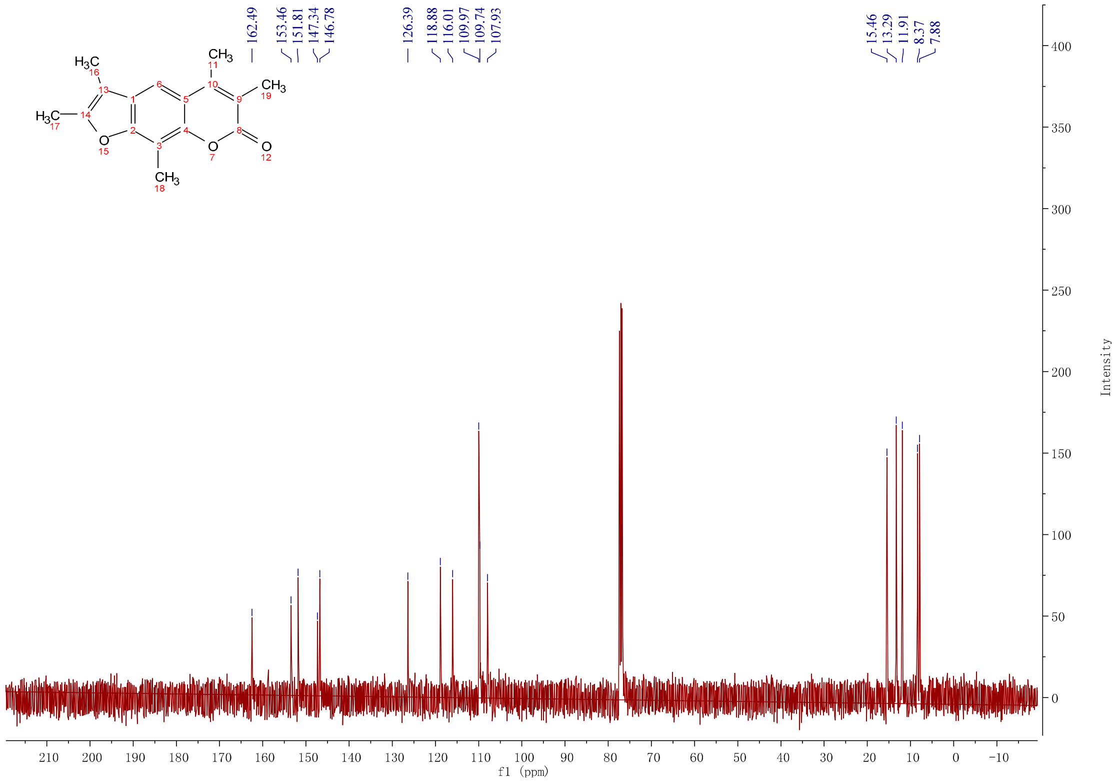
162.49
153.46
151.81
147.34
146.78
126.39
118.88
116.01
109.97
109.74
107.93
15.46
13.29
11.91
8.37
7.88
Intensity
f1 (ppm)

4c

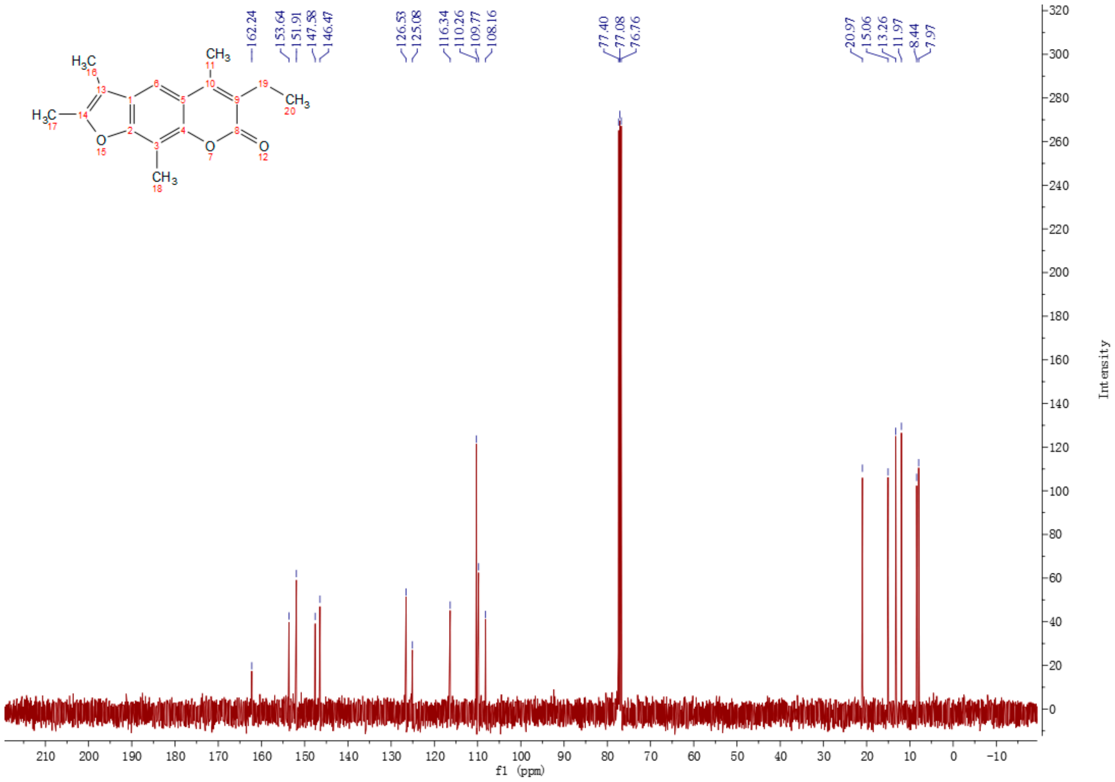
162.24
153.64
151.91
147.58
146.47
126.53
125.08
116.34
110.26
109.77
108.16
77.40
77.08
76.76
20.97
15.06
13.26
11.97
8.44
7.97
Intensity
f1 (ppm)

4d

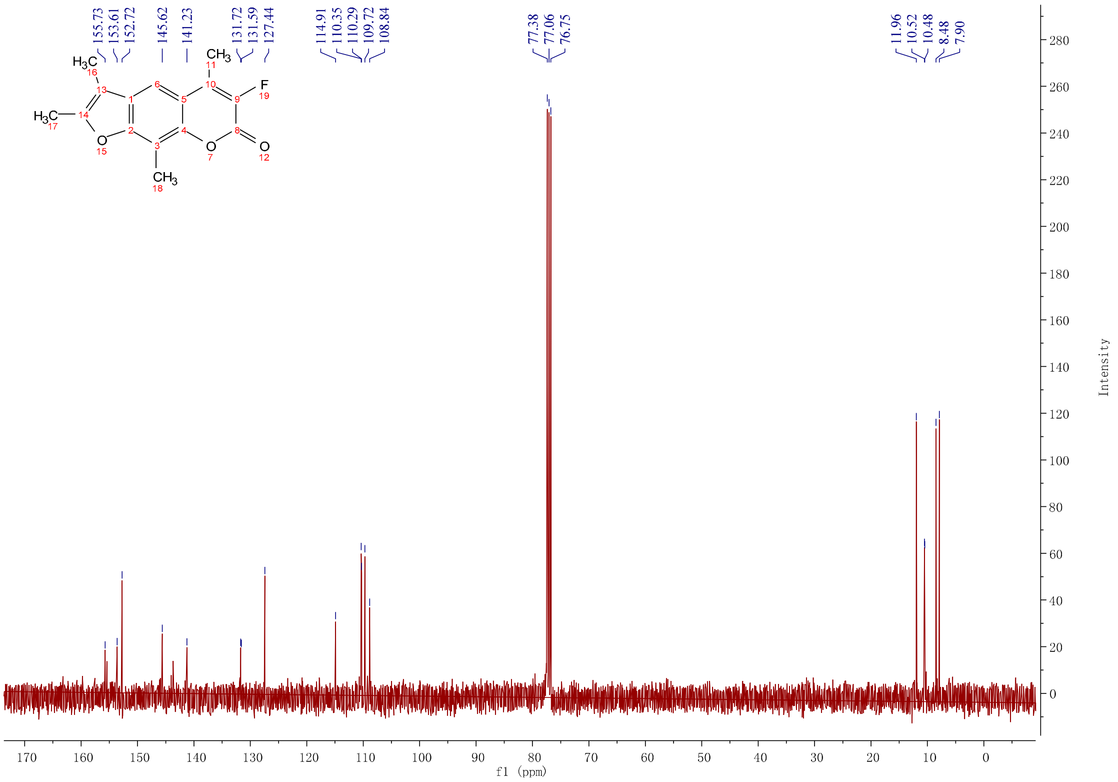
155.73
153.61
152.72
145.62
141.23
131.72
131.59
127.44
114.91
110.35
110.29
109.72
108.84
77.38
77.06
76.75
11.96
10.52
10.48
8.48
7.90
Intensity
f1 (ppm)

4e

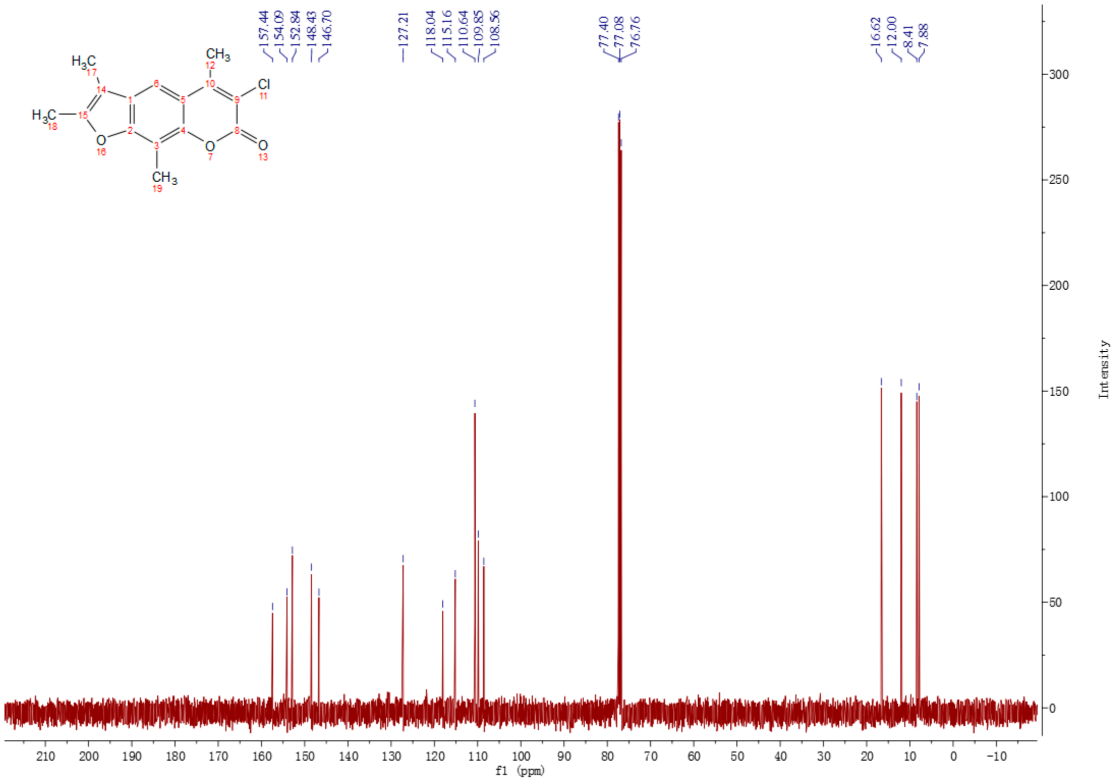
157.44
154.09
152.84
148.43
146.70
127.21
118.04
115.16
110.64
109.85
108.56
77.40
77.08
76.76
16.62
12.00
8.41
7.88
f1 (ppm)
Intensity

4f

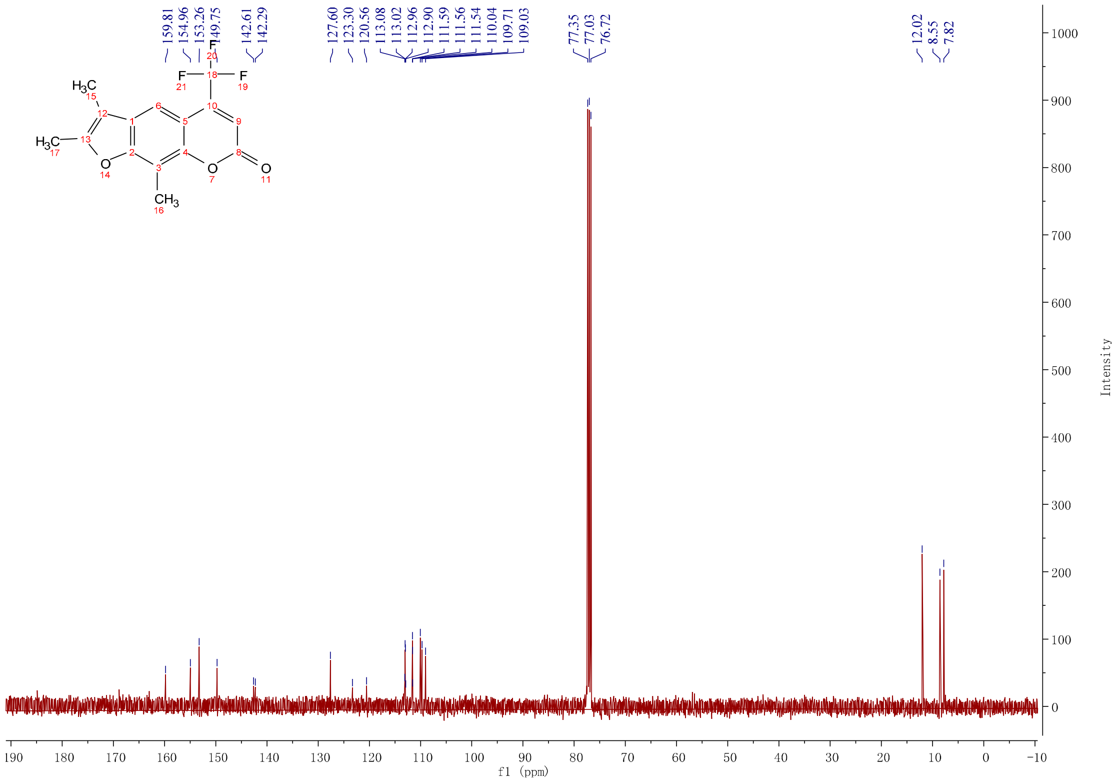
159.81
154.96
153.26
149.75
142.61
142.29
127.60
123.30
120.56
113.08
113.02
112.96
112.90
111.59
111.56
111.54
110.04
109.71
109.03
77.35
77.03
76.72
12.02
8.55
7.82
f1 (ppm)
Intensity

5a

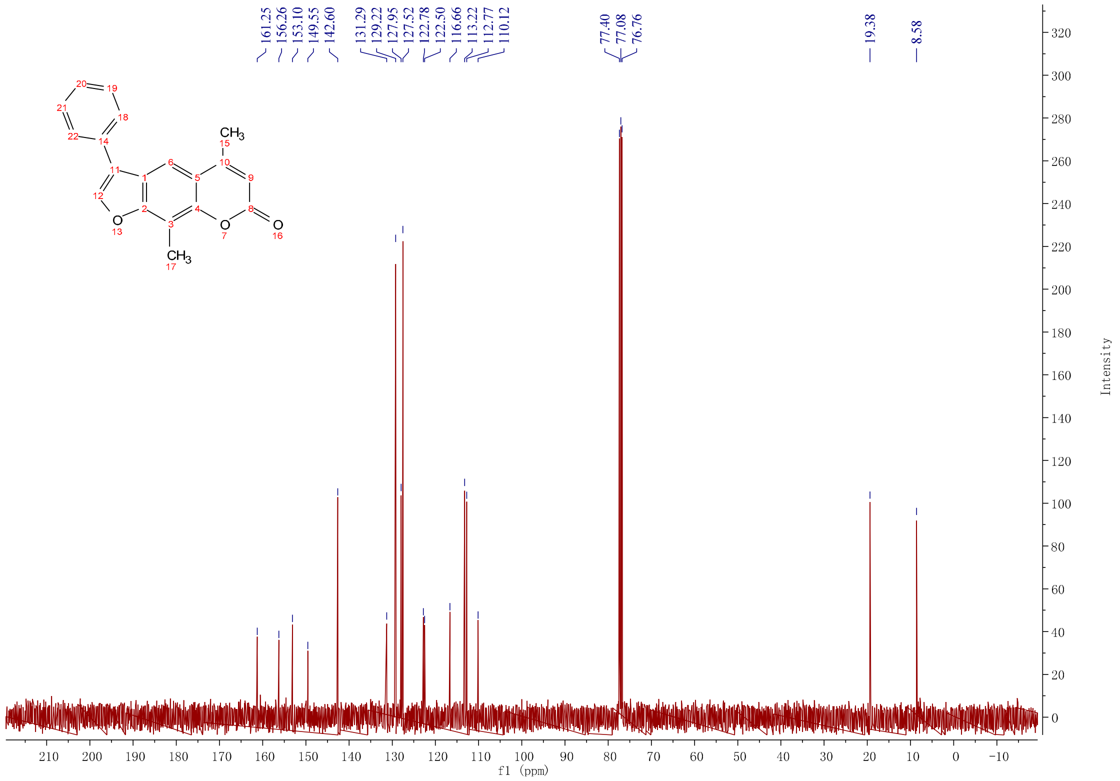
161.25
156.26
153.10
149.55
142.60
131.29
129.22
127.95
127.52
122.78
122.50
116.66
113.22
112.77
110.12
77.40
77.08
76.76
19.38
8.58
CH3
f1 (ppm)
Intensity

5b

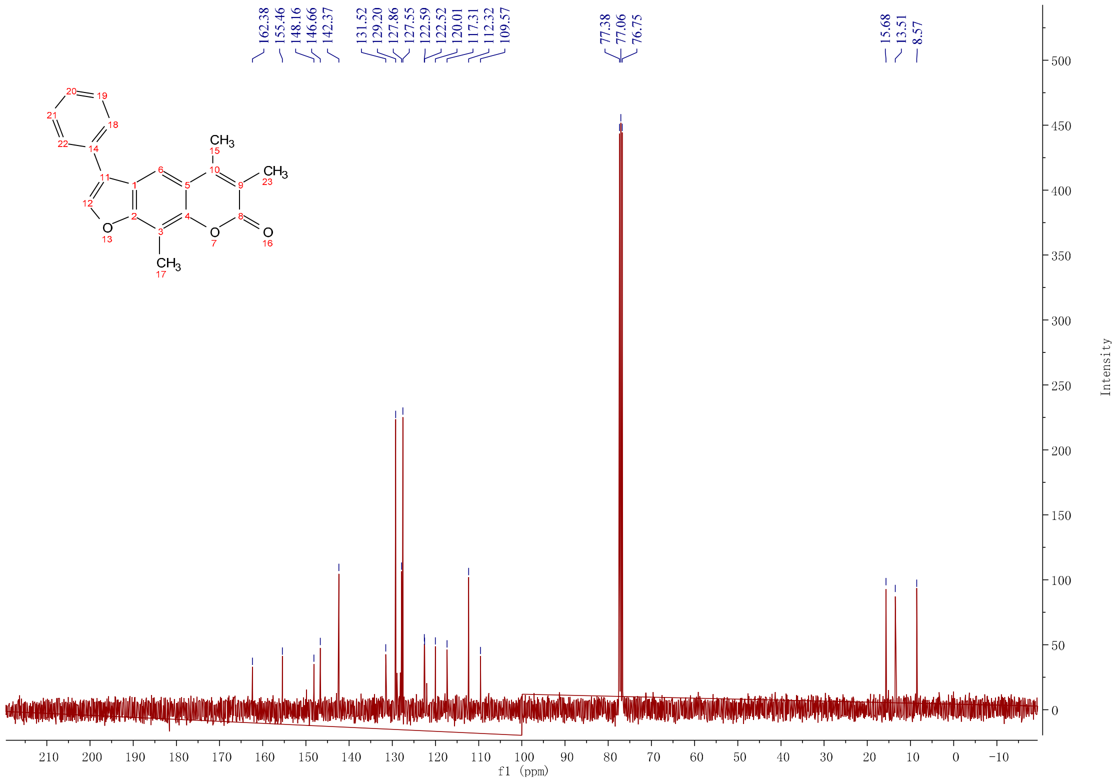
162.38
155.46
148.16
146.66
142.37
131.52
129.20
127.86
127.55
122.59
122.52
120.01
117.31
112.32
109.57
77.38
77.06
76.75
15.68
13.51
8.57
CH3
f1 (ppm)
Intensity

5c

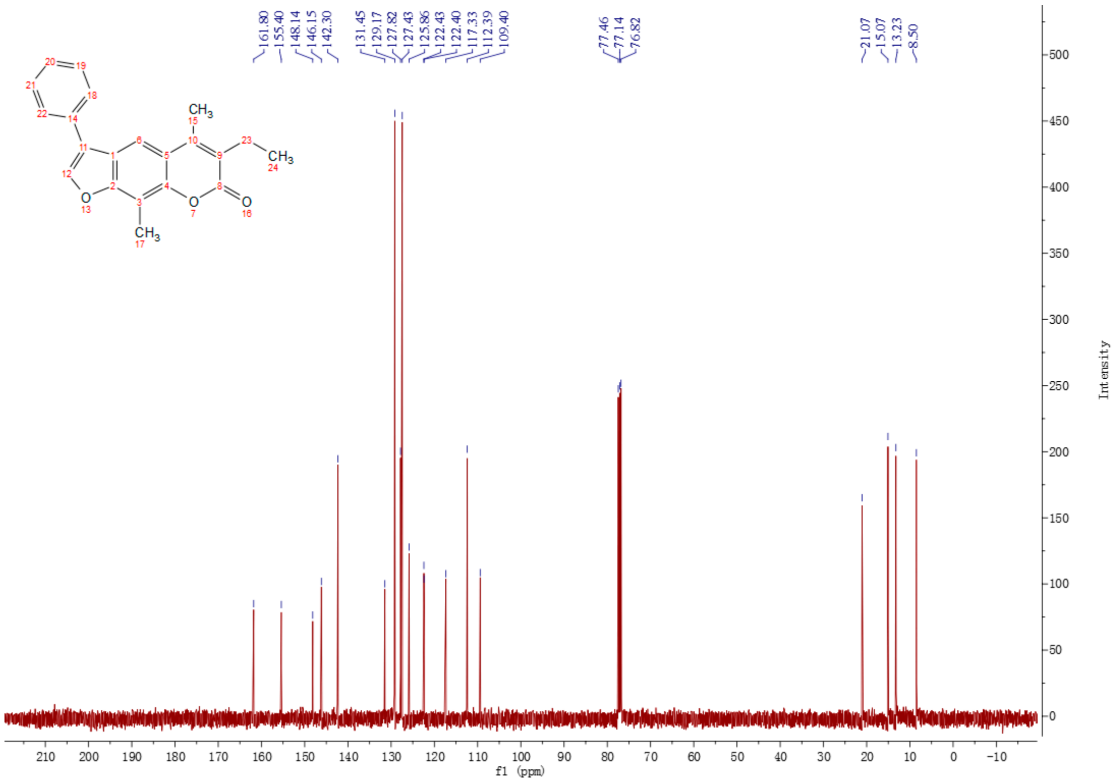
161.80
155.40
148.14
146.15
142.30
131.45
129.17
127.82
127.43
125.86
122.43
122.40
117.33
112.39
109.40
77.46
77.14
76.82
21.07
15.07
13.23
8.50
CH3
CH3
CH3
O
O
O
f1 (ppm)
Intensity

5d

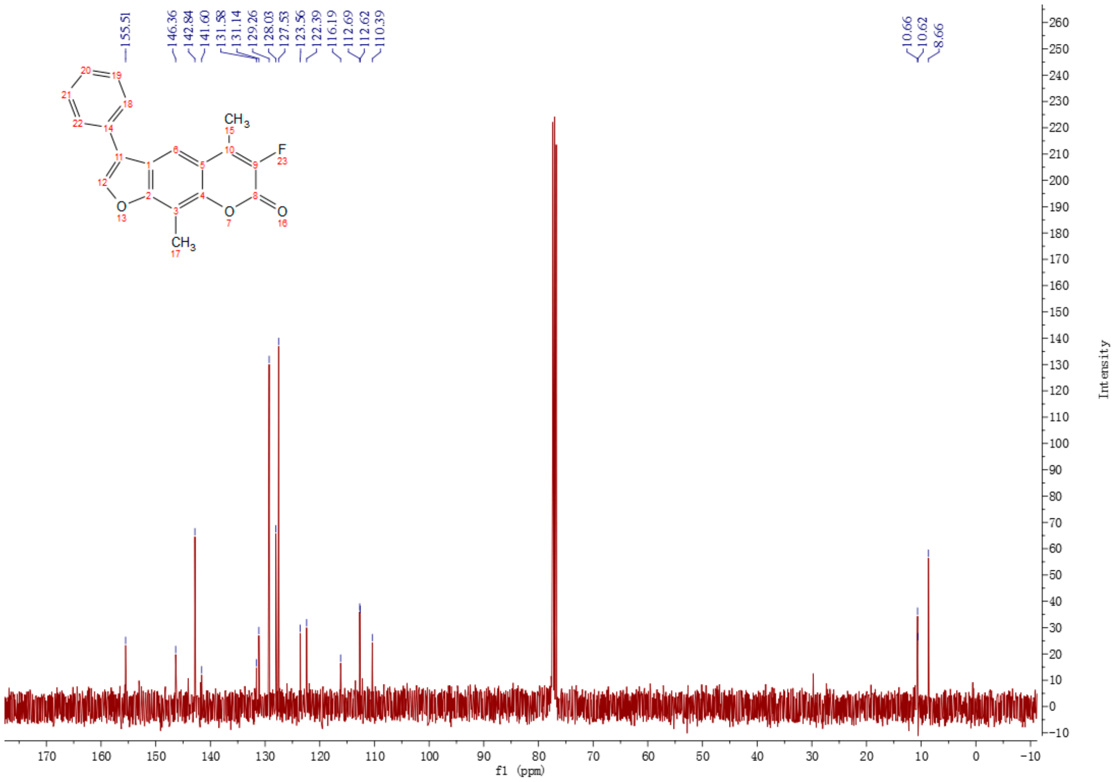
155.51
146.36
142.84
141.60
131.58
131.14
129.26
128.03
127.53
123.56
122.39
116.19
112.69
112.62
110.39
10.66
10.62
8.66
CH3
CH3
F
O
O
O
f1 (ppm)
Intensity

5e

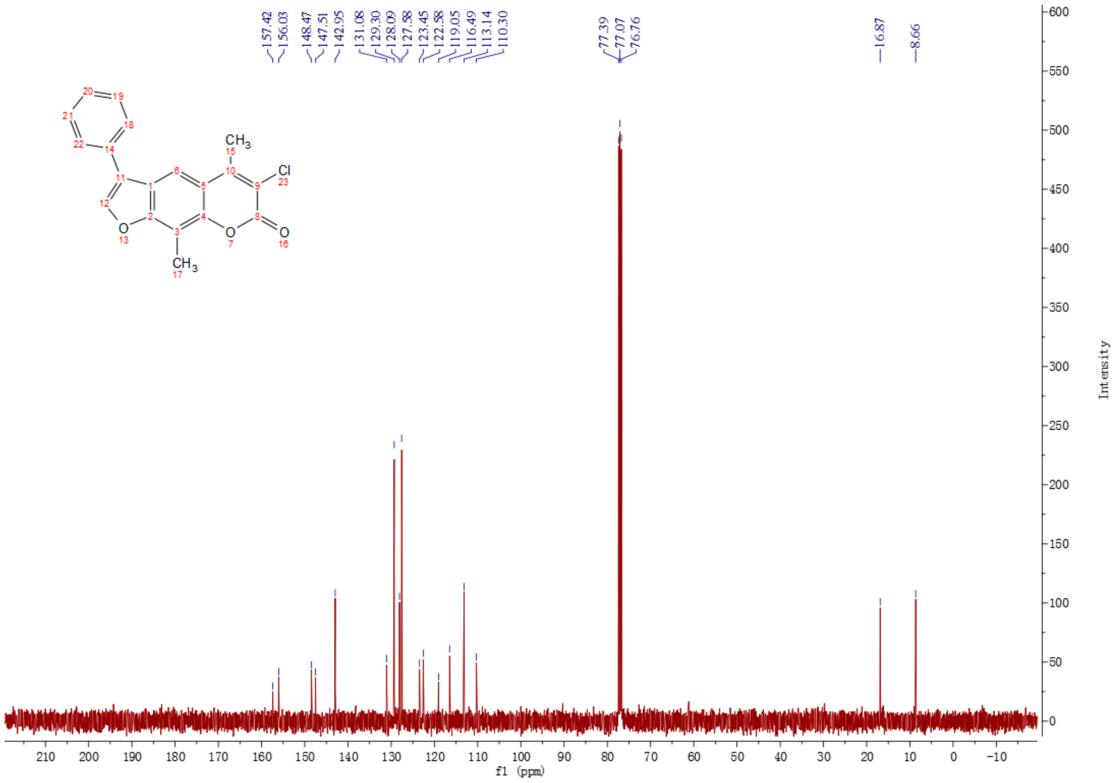

5f

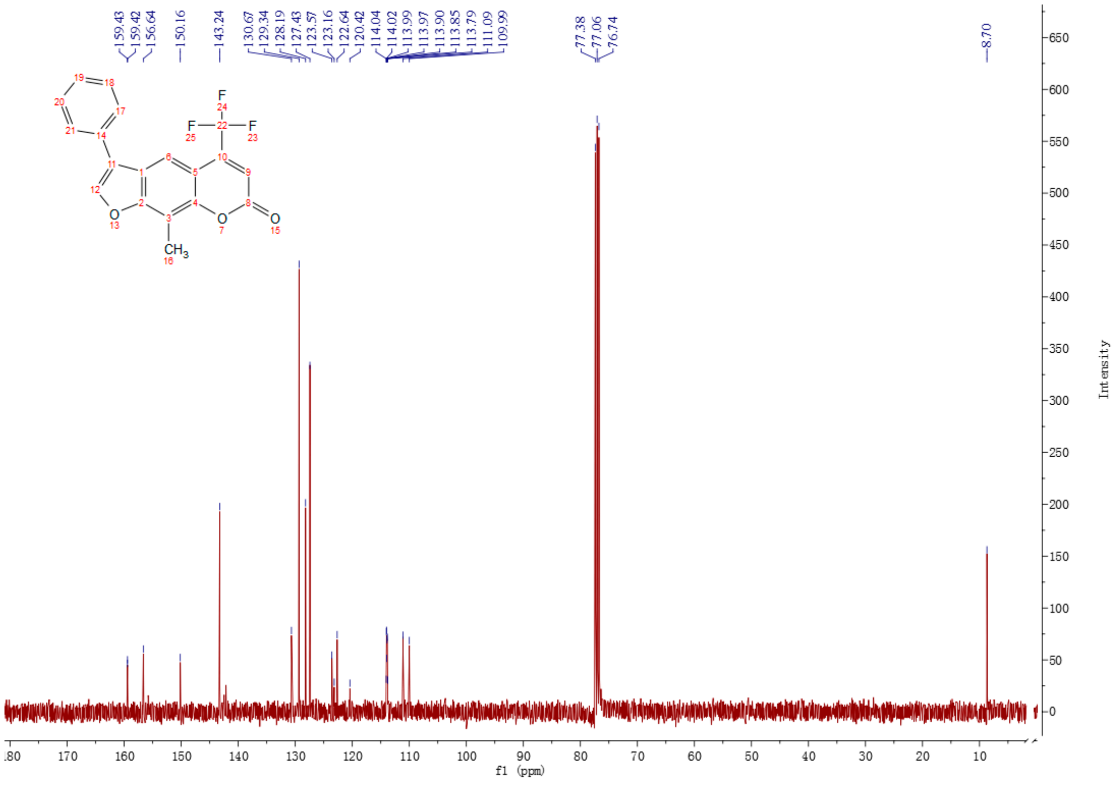

6a

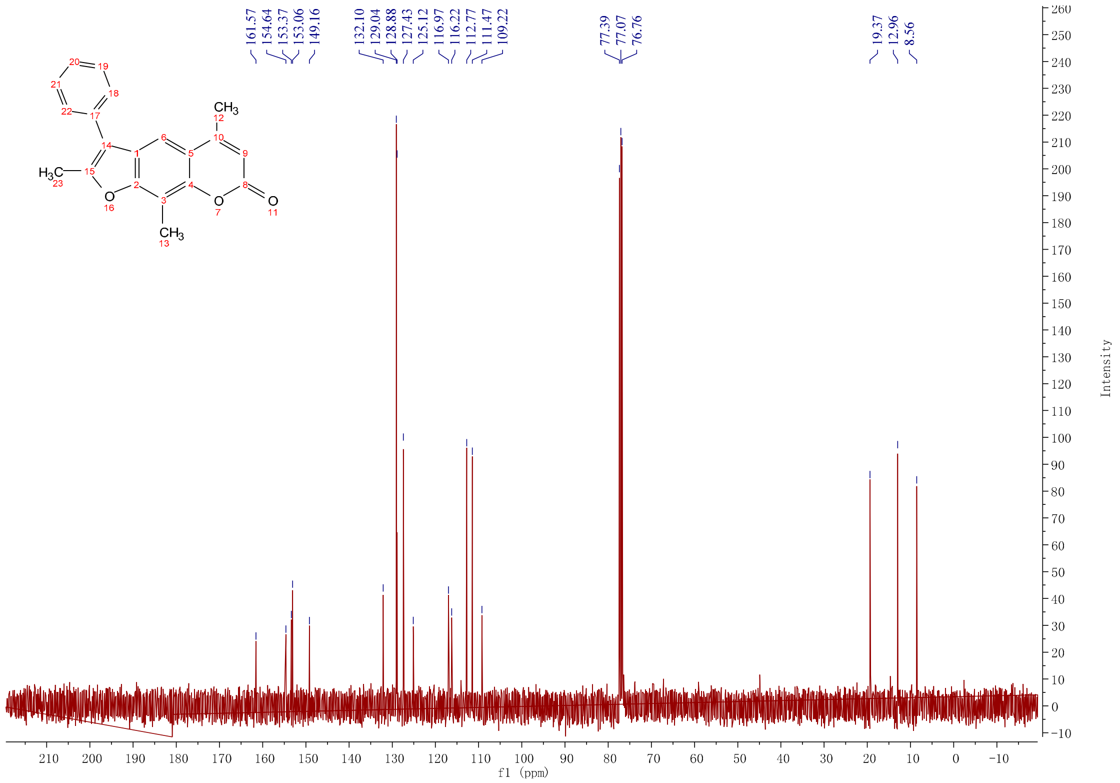
161.57
154.64
153.37
153.06
149.16
132.10
129.04
128.88
127.43
125.12
116.97
116.22
112.77
111.47
109.22
77.39
77.07
76.76
19.37
12.96
8.56
f1 (ppm)
Intensity

6c

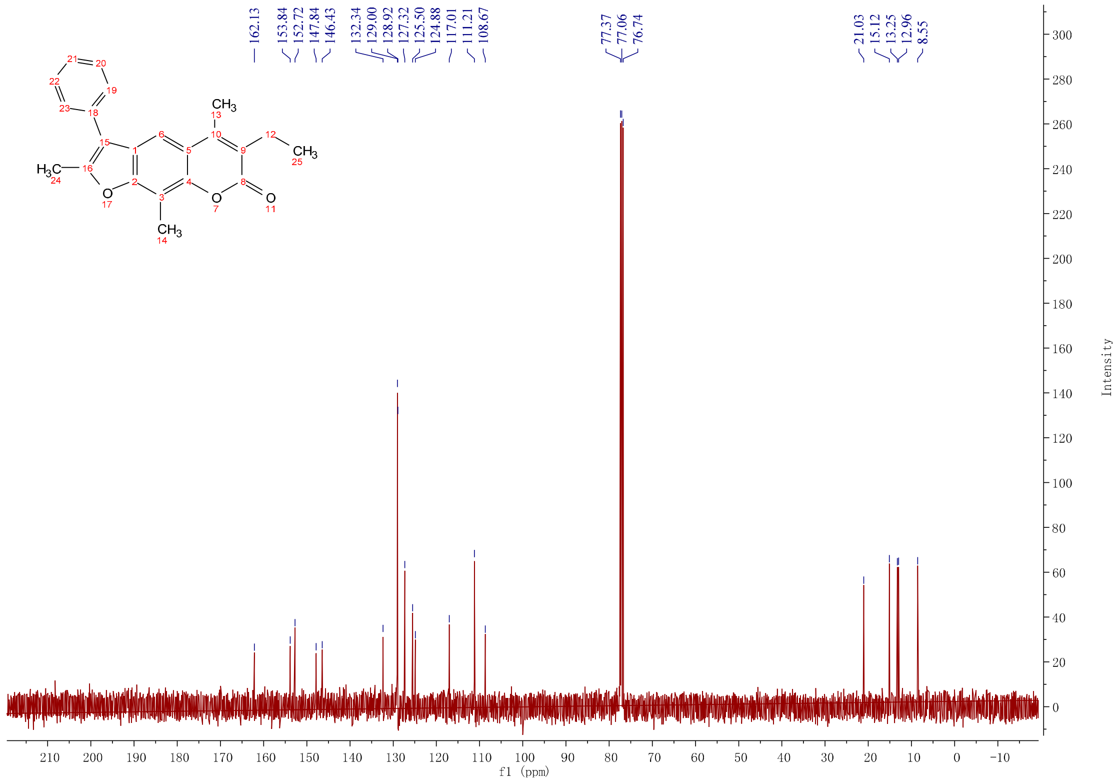
162.13
153.84
152.72
147.84
146.43
132.34
129.00
128.92
127.32
125.50
124.88
117.01
111.21
108.67
77.37
77.06
76.74
21.03
15.12
13.25
12.96
8.55
f1 (ppm)
Intensity

6d

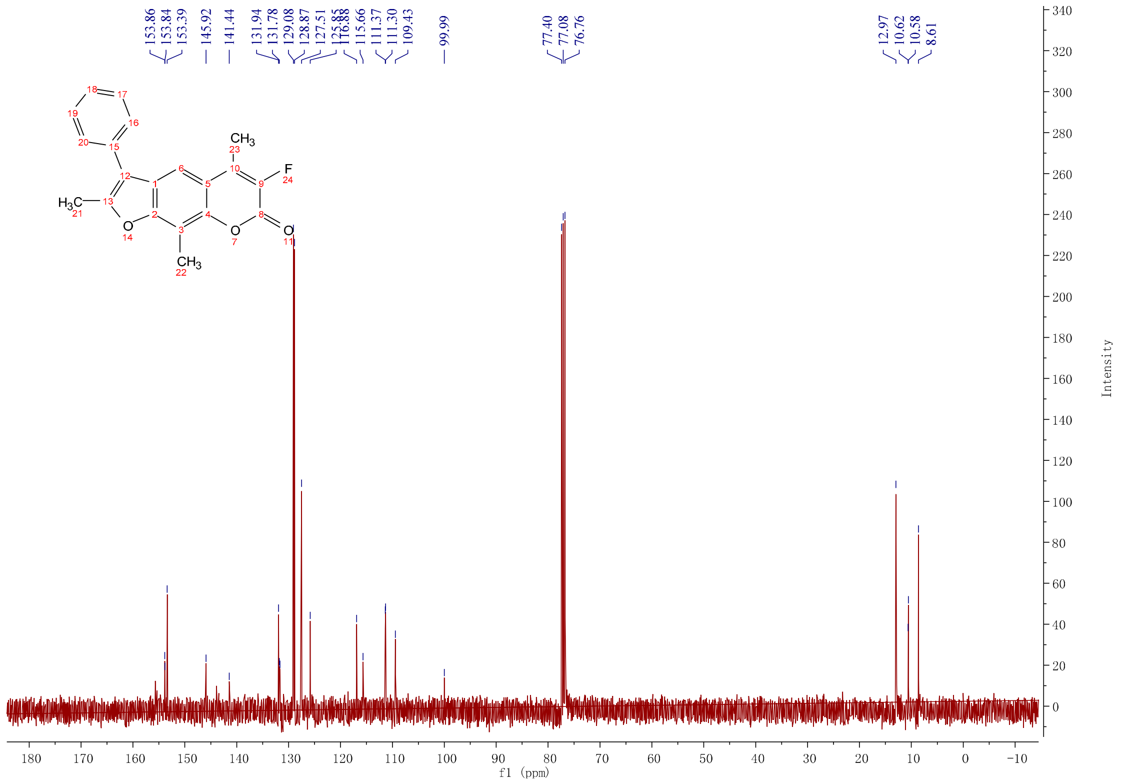
153.86
153.84
153.39
145.92
141.44
131.94
131.78
129.08
128.87
127.51
125.85
116.88
115.66
111.37
111.30
109.43
99.99
77.40
77.08
76.76
12.97
10.62
10.58
8.61
CH3
F
H3C
O
O
O
CH3
Intensity
f1 (ppm)

6e

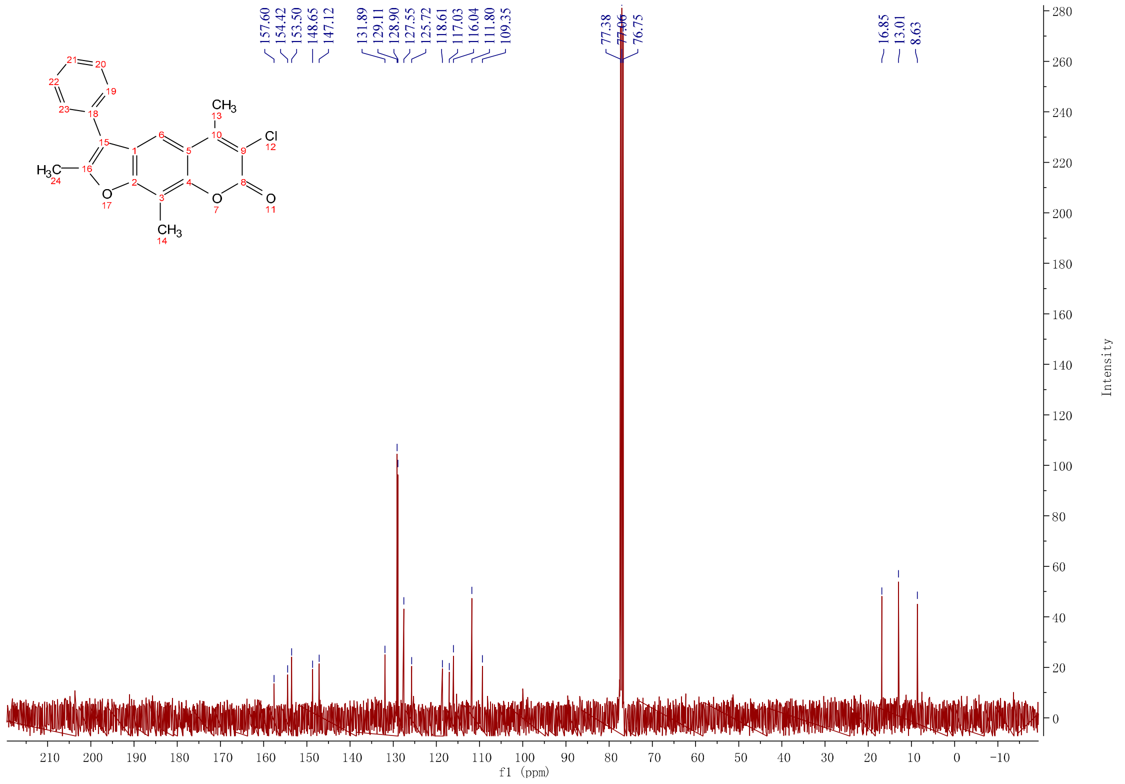
157.60
154.42
153.50
148.65
147.12
131.89
129.11
128.90
127.55
125.72
118.61
117.03
116.04
111.80
109.35
77.38
77.06
76.75
16.85
13.01
8.63
CH3
Cl
H3C
O
O
O
CH3
Intensity
f1 (ppm)

6f

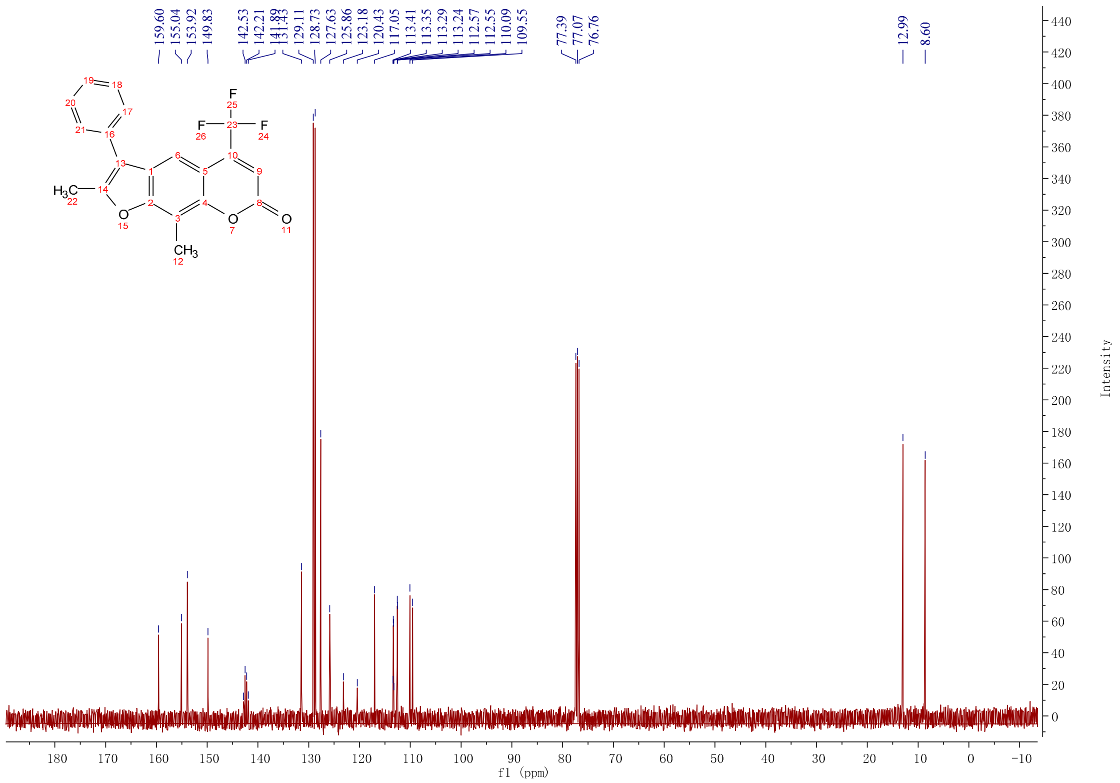

159.60
155.04
153.92
149.83
142.53
142.21
141.89
131.43
129.11
128.73
127.63
125.86
123.18
120.43
117.05
113.41
113.35
113.29
113.24
112.57
112.55
110.09
109.55
77.39
77.07
76.76
12.99
8.60
f1 (ppm)
Intensity

2. HRMS Spectra of New Compounds

3a

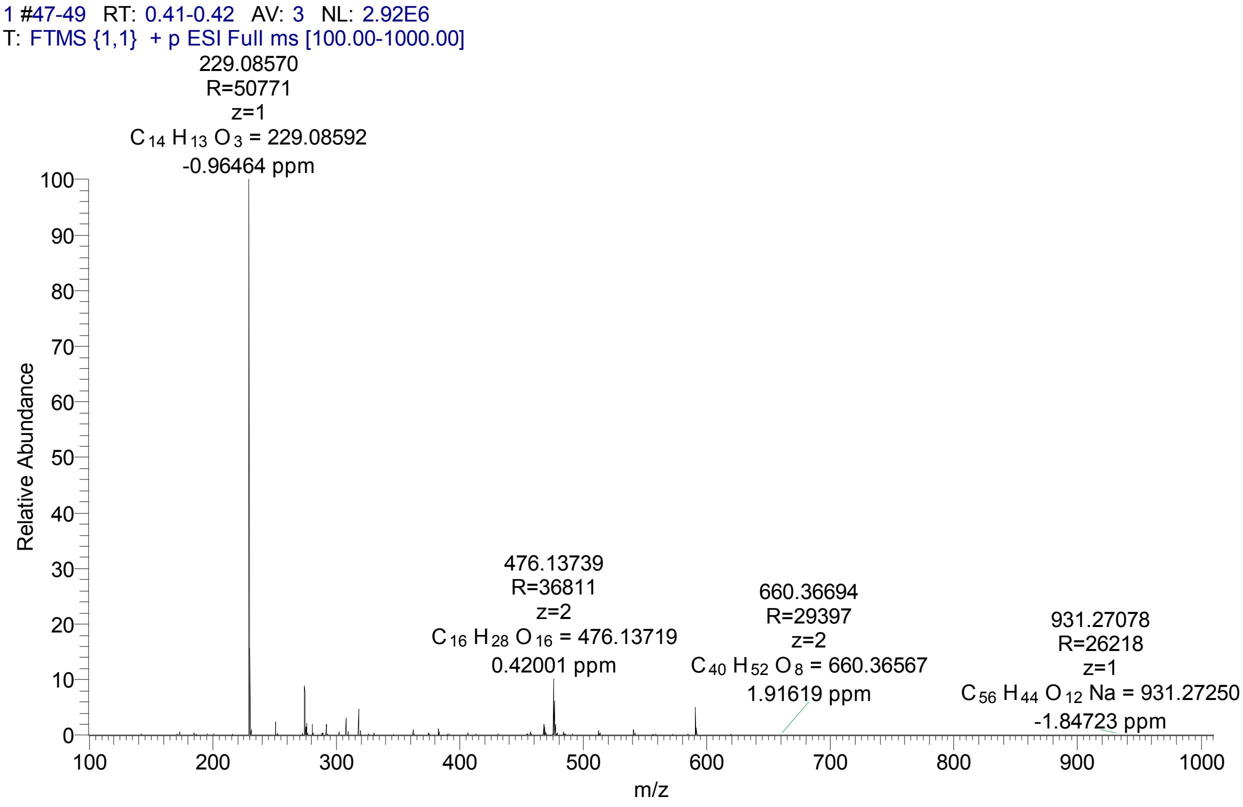

3b

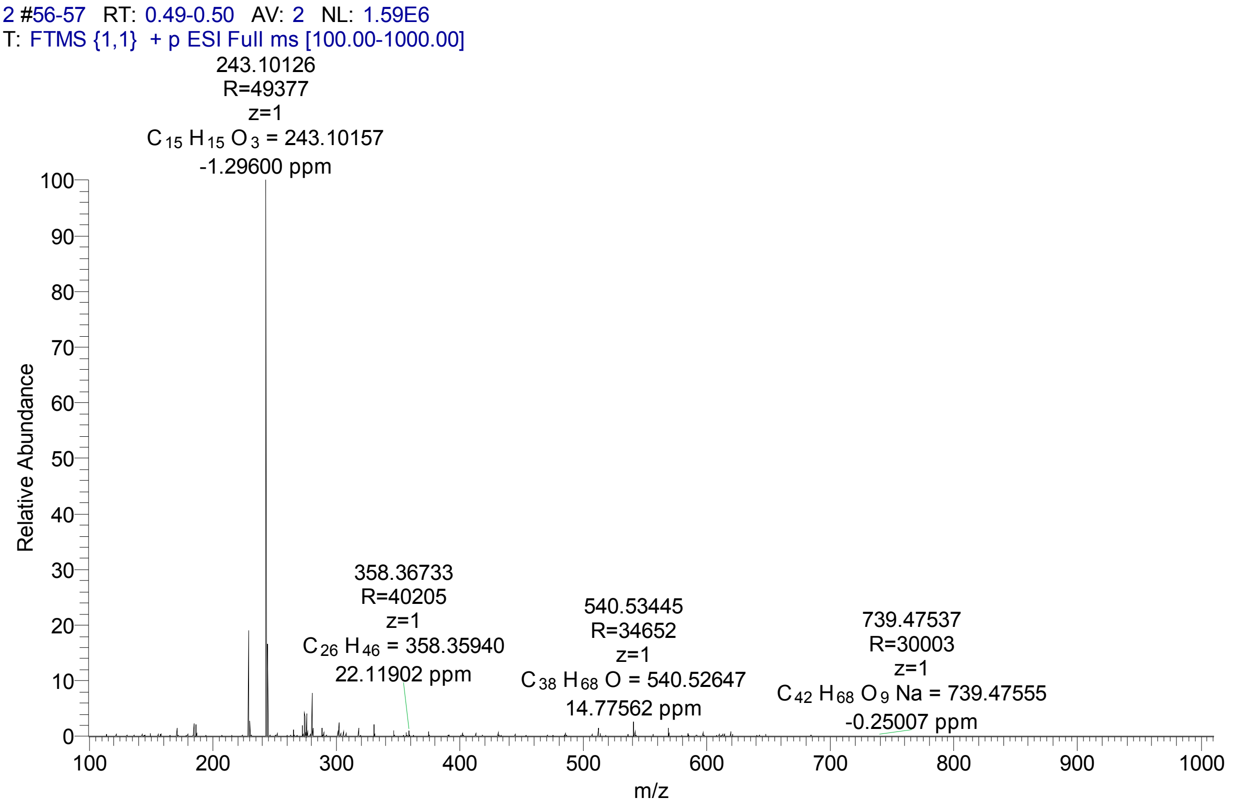

3c

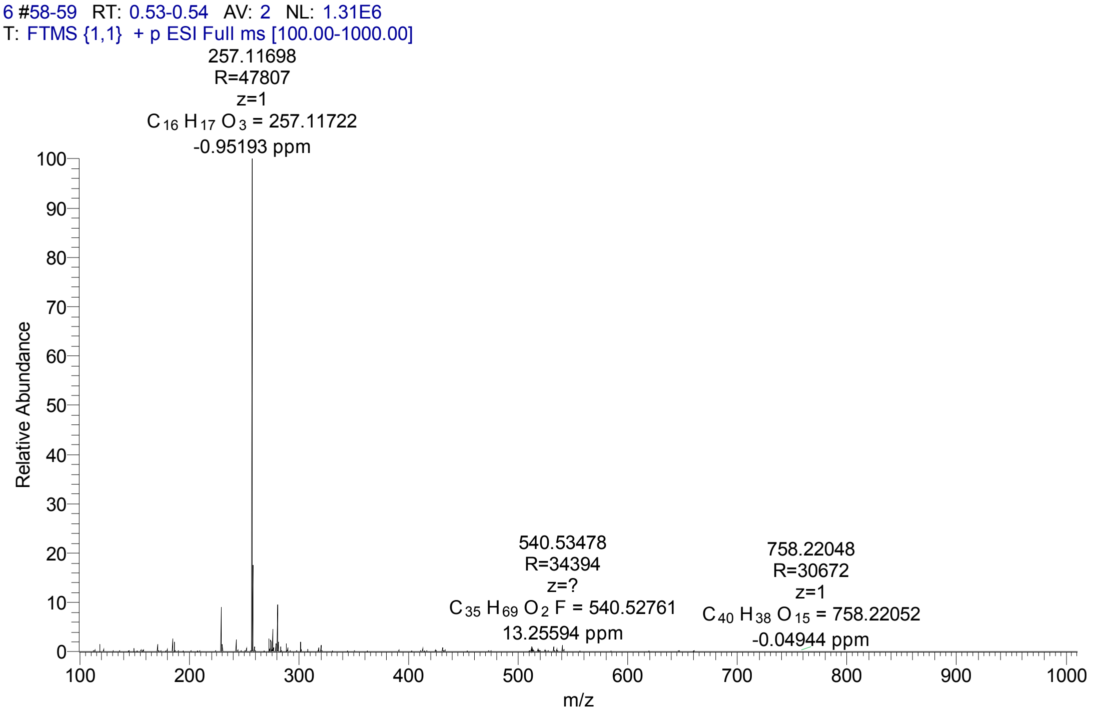

6 #58-59 RT: 0.53-0.54 AV: 2 NL: 1.31E6
T: FTMS {1,1} + p ESI Full ms [100.00-1000.00]
257.11698
R=47807
z=1
C 16 H 17 O 3 = 257.11722
-0.95193 ppm
540.53478
R=34394
z=?
C 35 H 69 O 2 F = 540.52761
13.25594 ppm
758.22048
R=30672
z=1
C 40 H 38 O 15 = 758.22052
-0.04944 ppm
Relative Abundance
m/z

3d

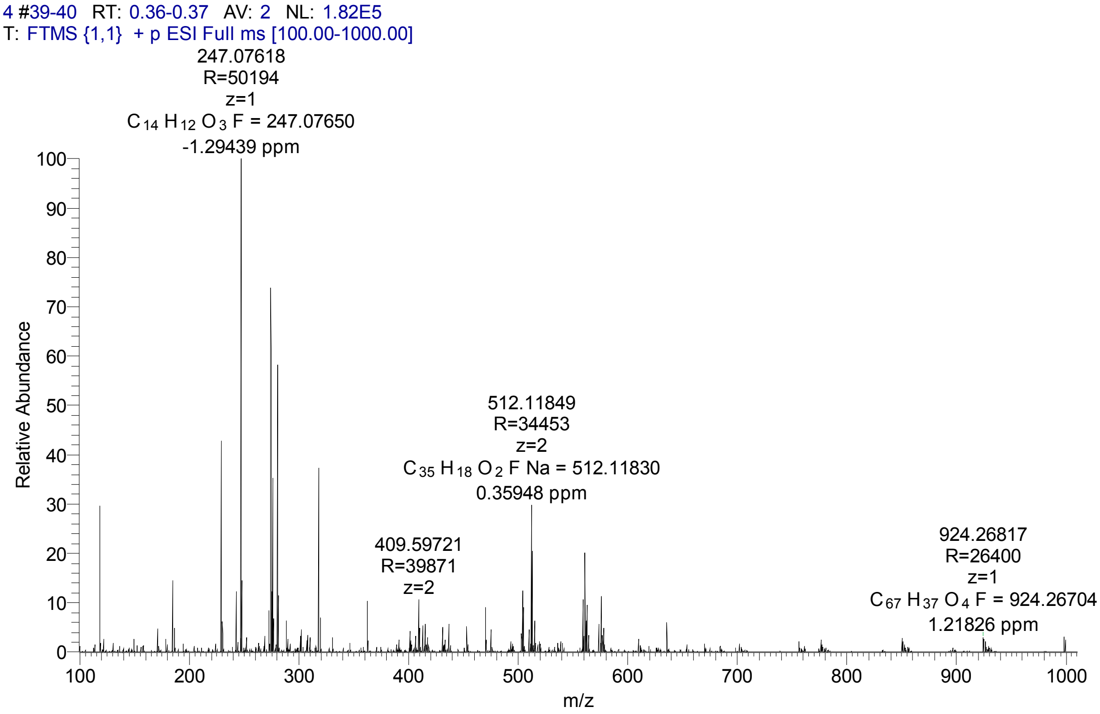

4 #39-40 RT: 0.36-0.37 AV: 2 NL: 1.82E5
T: FTMS {1,1} + p ESI Full ms [100.00-1000.00]
247.07618
R=50194
z=1
C 14 H 12 O 3 F = 247.07650
-1.29439 ppm
512.11849
R=34453
z=2
C 35 H 18 O 2 F Na = 512.11830
0.35948 ppm
409.59721
R=39871
z=2
924.26817
R=26400
z=1
C 67 H 37 O 4 F = 924.26704
1.21826 ppm
Relative Abundance
m/z

3e

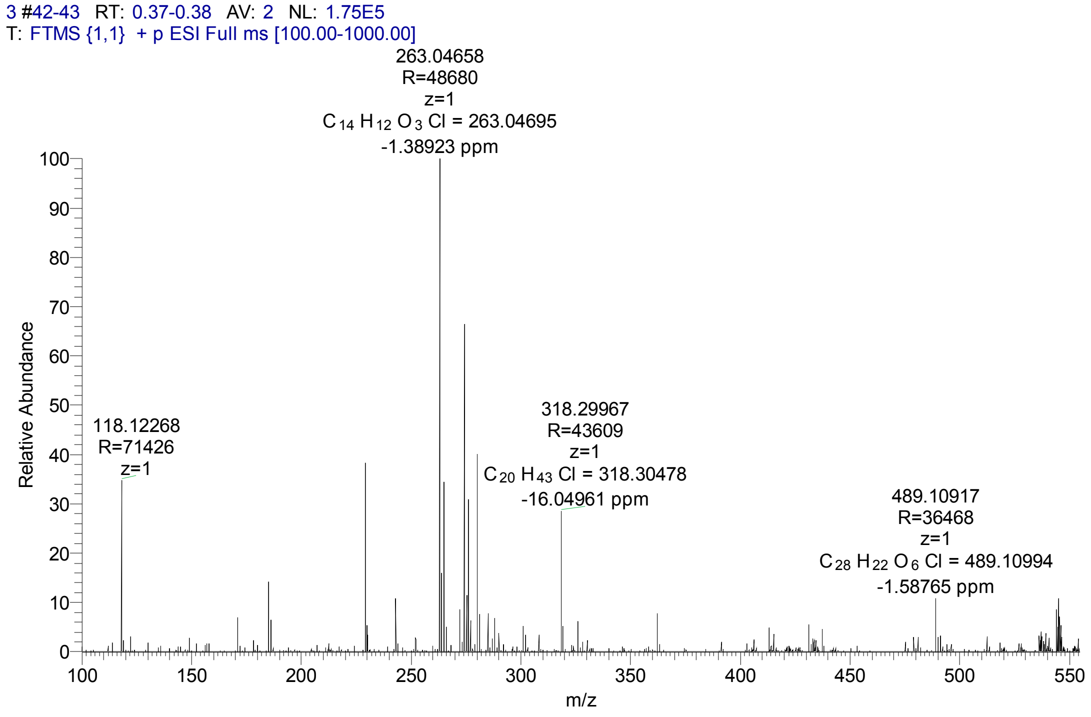
3 #42-43 RT: 0.37-0.38 AV: 2 NL: 1.75E5
T: FTMS {1,1} + p ESI Full ms [100.00-1000.00]
263.04658
R=48680
z=1
C 14 H 12 O 3 Cl = 263.04695
-1.38923 ppm
118.12268
R=71426
z=1
318.29967
R=43609
z=1
C 20 H 43 Cl = 318.30478
-16.04961 ppm
489.10917
R=36468
z=1
C 28 H 22 O 6 Cl = 489.10994
-1.58765 ppm
Relative Abundance
m/z

3f

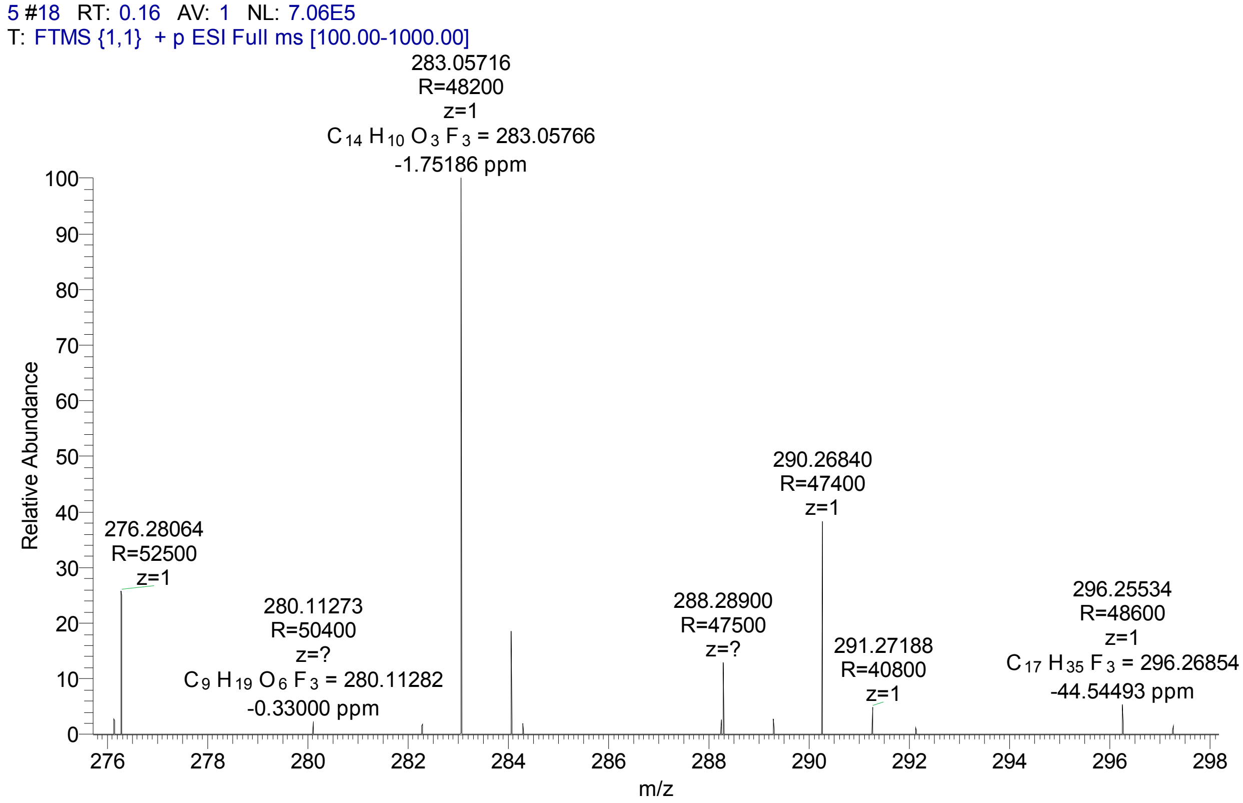
5 #18 RT: 0.16 AV: 1 NL: 7.06E5
T: FTMS {1,1} + p ESI Full ms [100.00-1000.00]
283.05716
R=48200
z=1
C 14 H 10 O 3 F 3 = 283.05766
-1.75186 ppm
276.28064
R=52500
z=1
280.11273
R=50400
z=?
C 9 H 19 O 6 F 3 = 280.11282
-0.33000 ppm
288.28900
R=47500
z=?
290.26840
R=47400
z=1
291.27188
R=40800
z=1
296.25534
R=48600
z=1
C 17 H 35 F 3 = 296.26854
-44.54493 ppm
Relative Abundance
m/z

4a

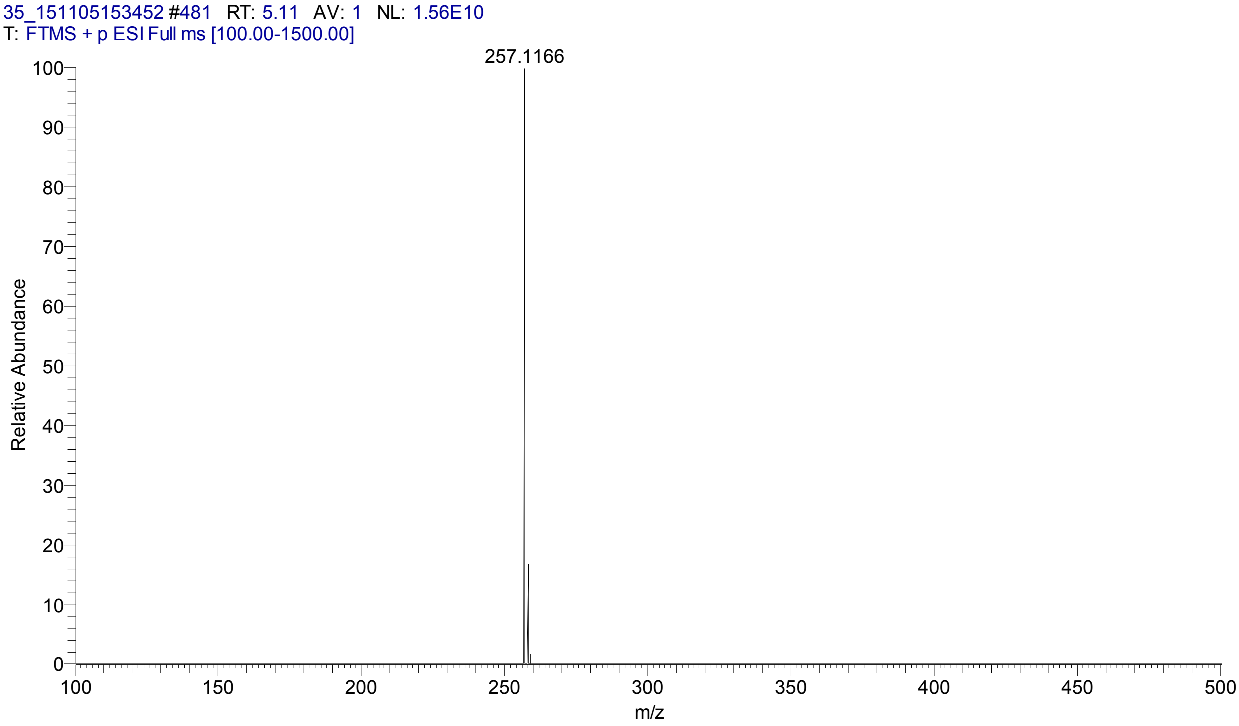
35_151105153452 #481 RT: 5.11 AV: 1 NL: 1.56E10
T: FTMS + p ESI Full ms [100.00-1500.00]
257.1166
Relative Abundance
m/z

4b

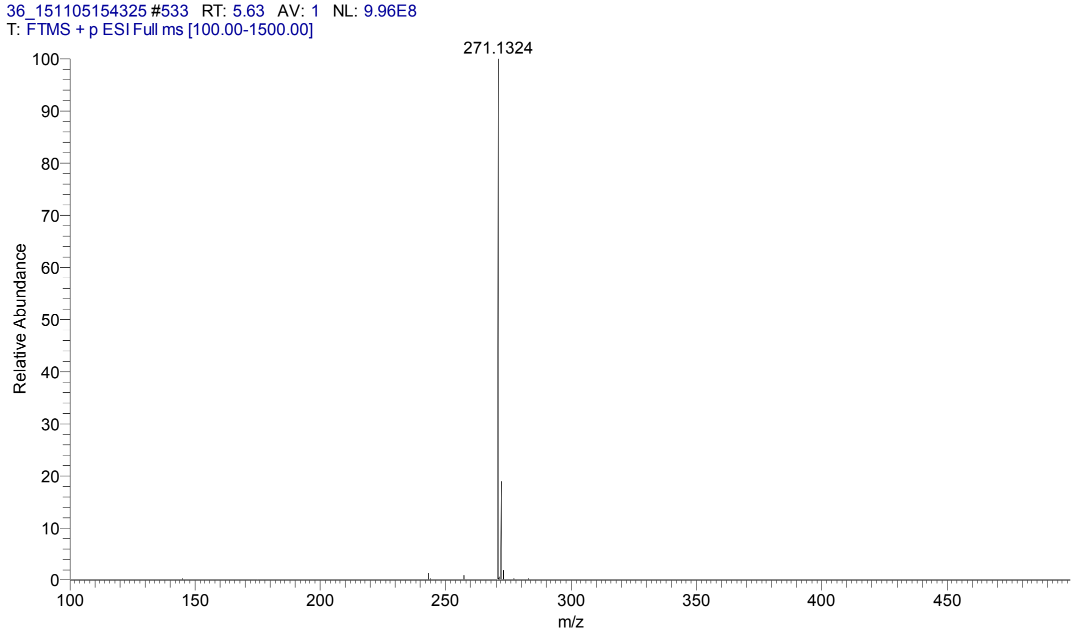
36_151105154325 #533 RT: 5.63 AV: 1 NL: 9.96E8
T: FTMS + p ESI Full ms [100.00-1500.00]
271.1324
Relative Abundance
m/z

4c

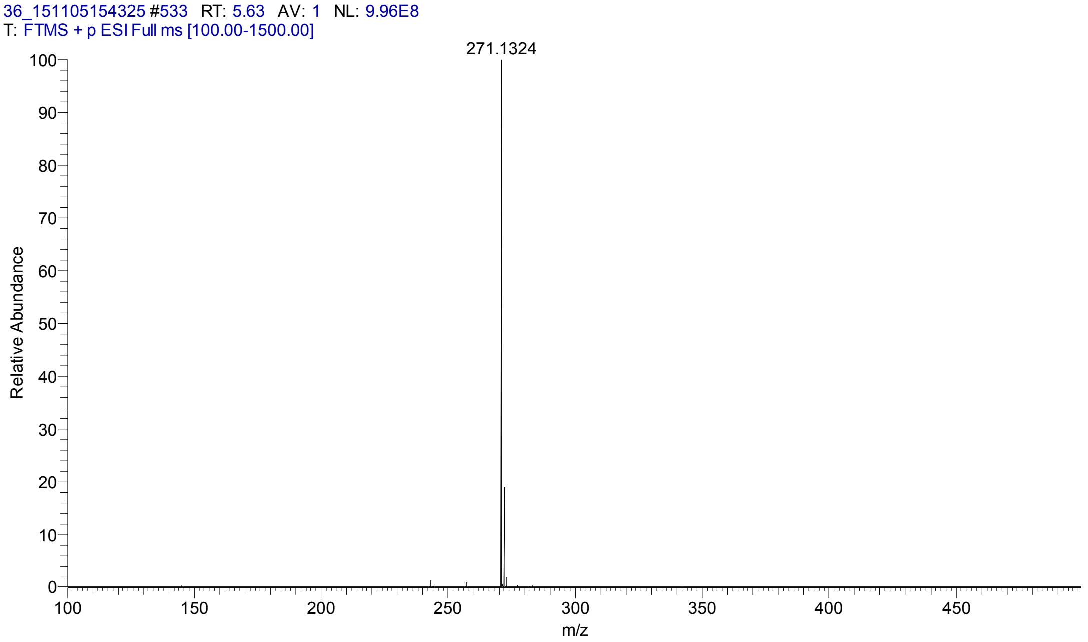
36_151105154325 #533 RT: 5.63 AV: 1 NL: 9.96E8
T: FTMS + p ESI Full ms [100.00-1500.00]
271.1324
Relative Abundance
m/z

4d

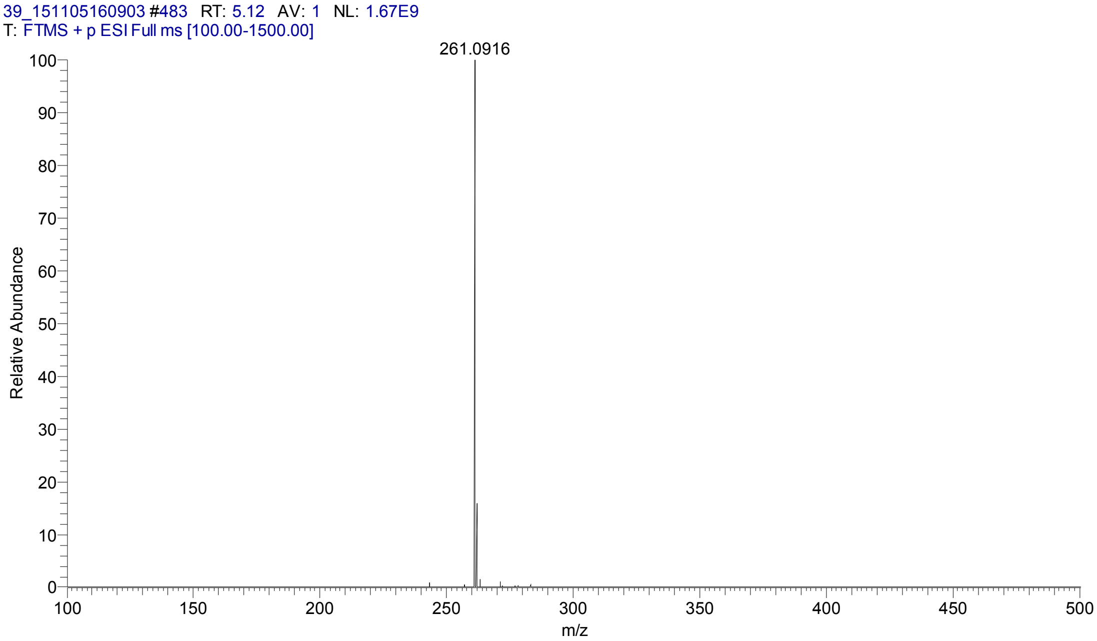
39_151105160903 #483 RT: 5.12 AV: 1 NL: 1.67E9
T: FTMS + p ESI Full ms [100.00-1500.00]
261.0916
Relative Abundance
m/z

4e

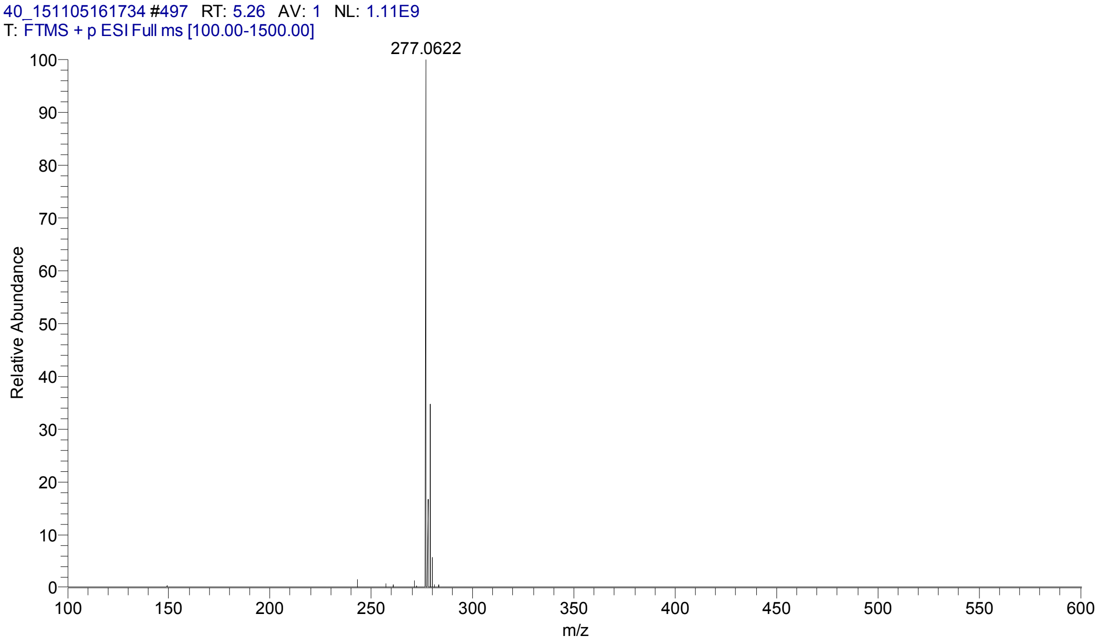
40_151105161734 #497 RT: 5.26 AV: 1 NL: 1.11E9
T: FTMS + p ESI Full ms [100.00-1500.00]
277.0622
Relative Abundance
m/z

4f

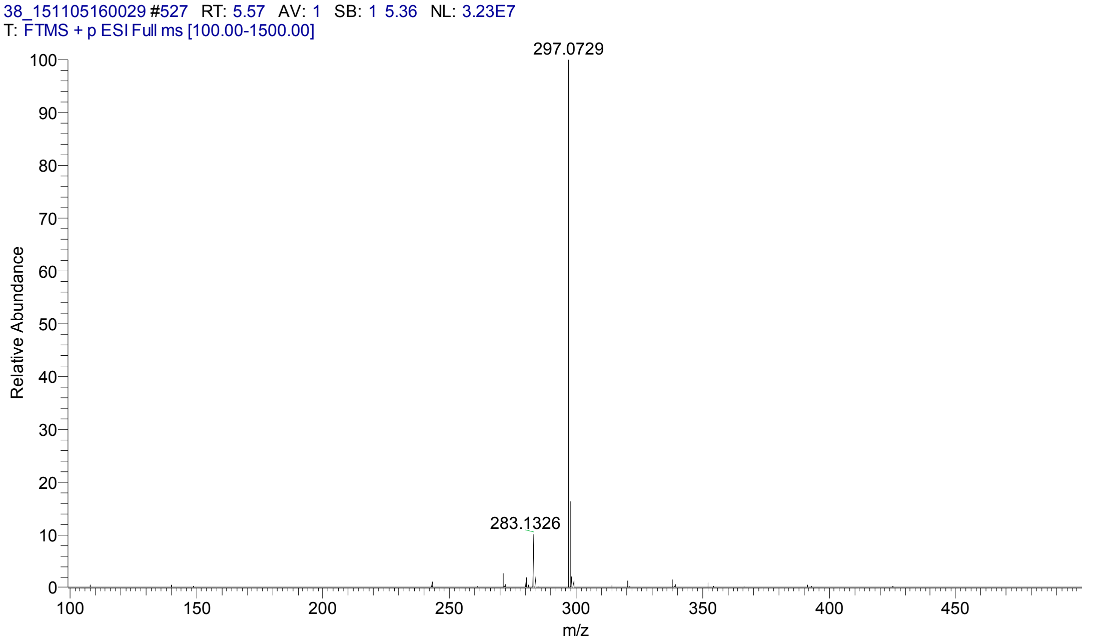
38_151105160029 #527 RT: 5.57 AV: 1 SB: 1 5.36 NL: 3.23E7
T: FTMS + p ESI Full ms [100.00-1500.00]
297.0729
283.1326
Relative Abundance
m/z

5a

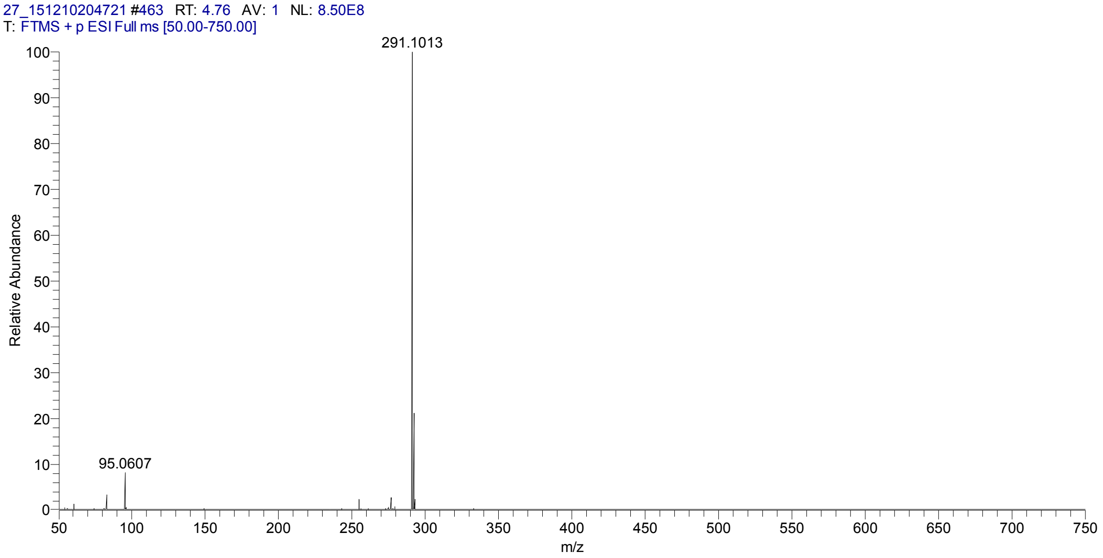
27_151210204721 #463 RT: 4.76 AV: 1 NL: 8.50E8
T: FTMS + p ESI Full ms [50.00-750.00]
291.1013
95.0607
Relative Abundance
m/z

5b

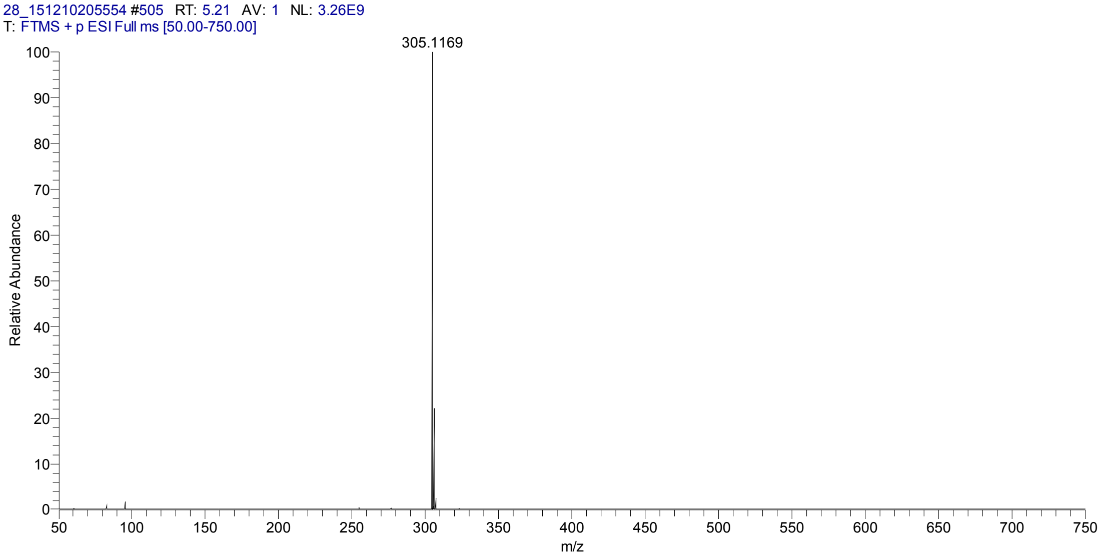
28_151210205554 #505 RT: 5.21 AV: 1 NL: 3.26E9
T: FTMS + p ESI Full ms [50.00-750.00]
305.1169
Relative Abundance
m/z

5c

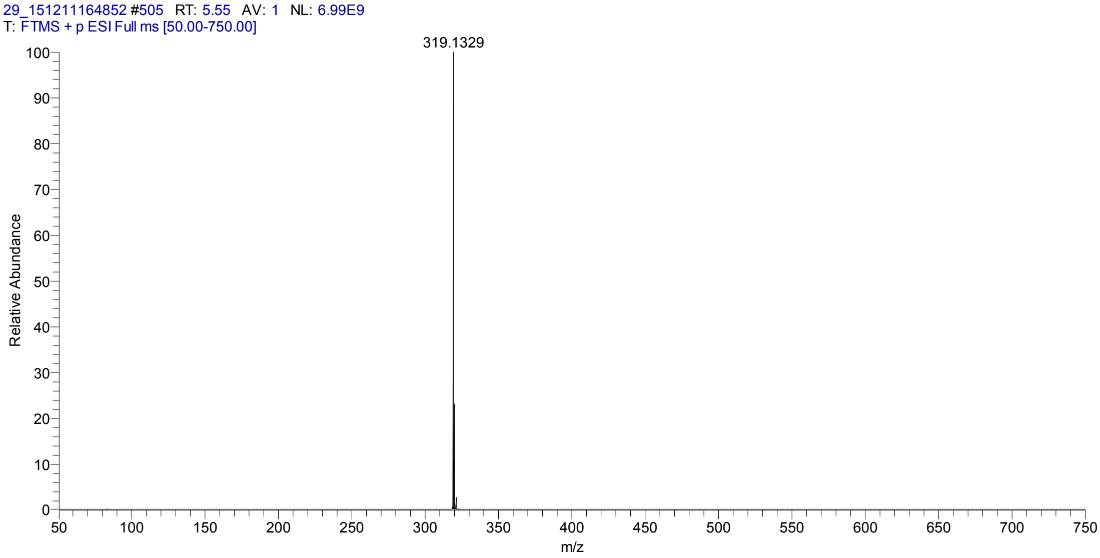

29_151211164852 #505 RT: 5.55 AV: 1 NL: 6.99E9
T: FTMS + p ESI Full ms [50.00-750.00]
319.1329
Relative Abundance
m/z

5d

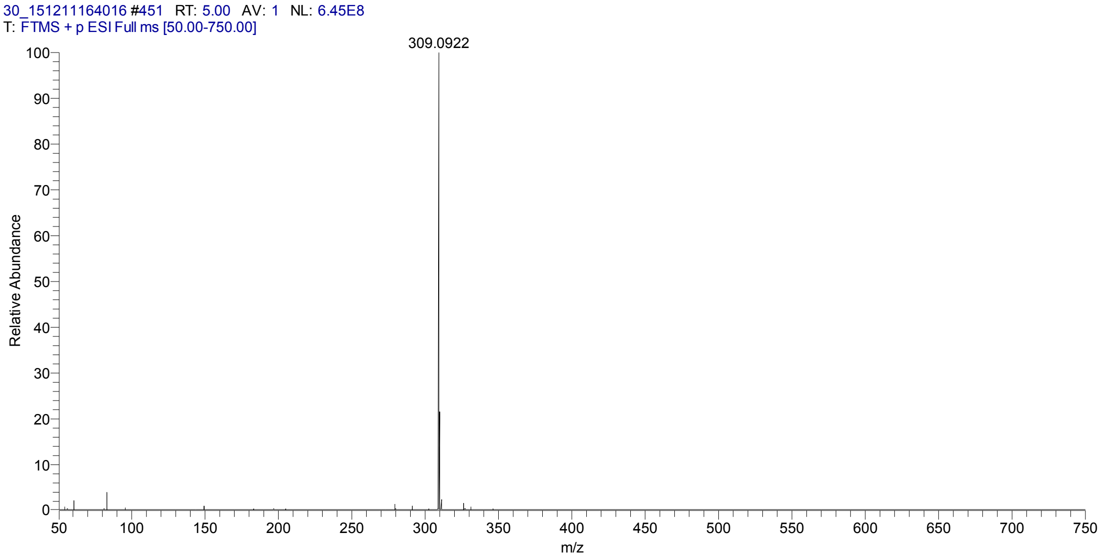

30_151211164016 #451 RT: 5.00 AV: 1 NL: 6.45E8
T: FTMS + p ESI Full ms [50.00-750.00]
309.0922
Relative Abundance
m/z

5e

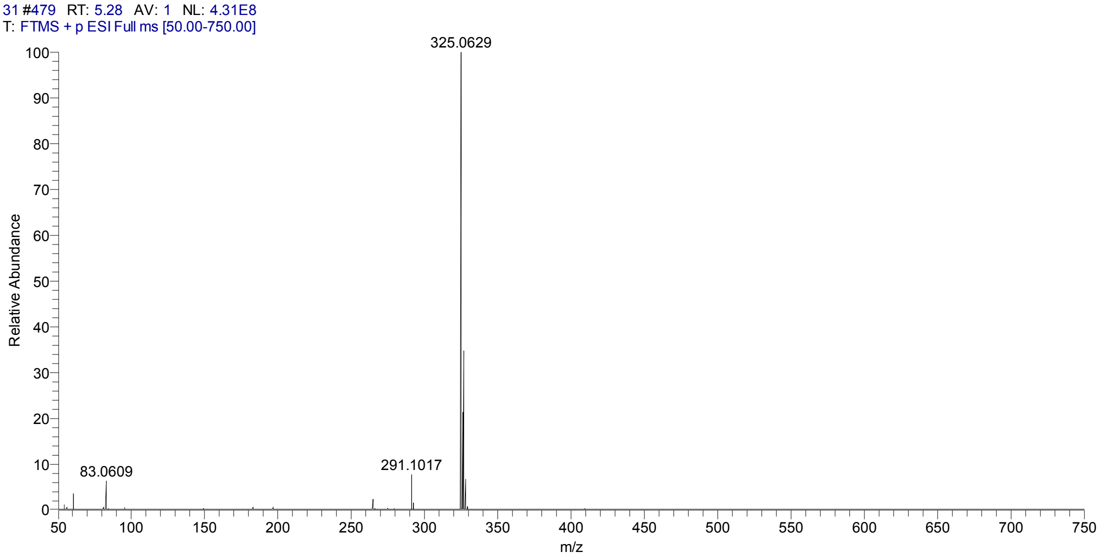

31 #479 RT: 5.28 AV: 1 NL: 4.31E8
T: FTMS + p ESI Full ms [50.00-750.00]
325.0629
291.1017
83.0609
Relative Abundance
m/z

5f

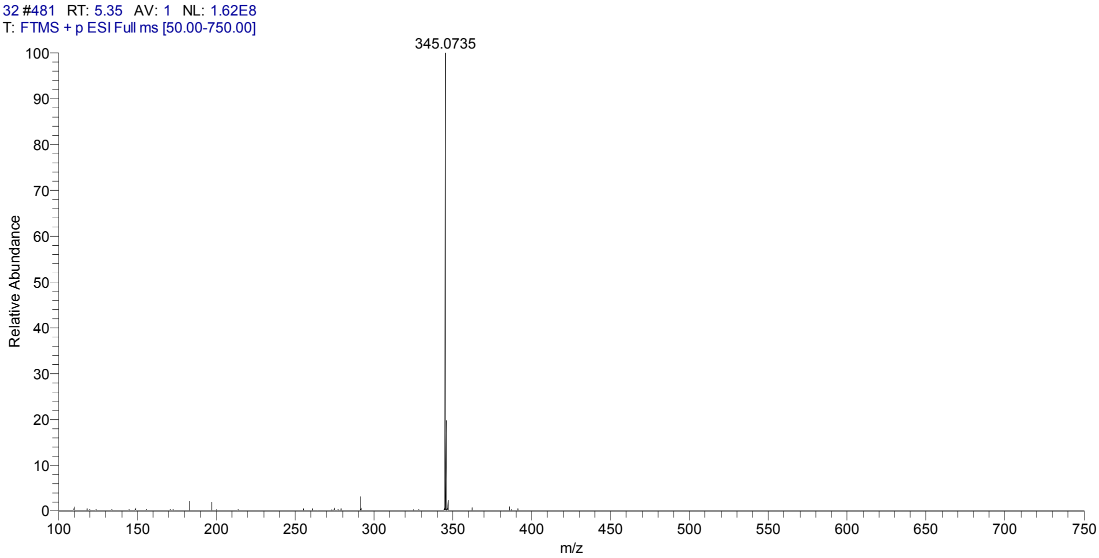

32 #481 RT: 5.35 AV: 1 NL: 1.62E8
T: FTMS + p ESI Full ms [50.00-750.00]
345.0735
Relative Abundance
m/z

6a

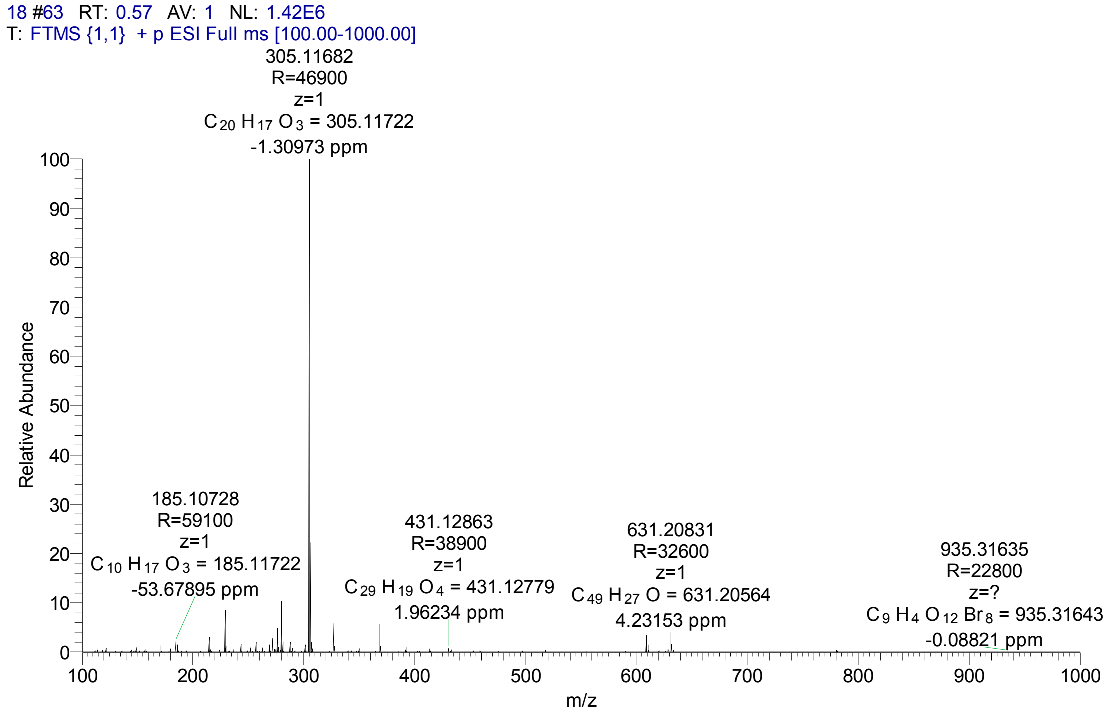
18 #63 RT: 0.57 AV: 1 NL: 1.42E6
T: FTMS {1,1} + p ESI Full ms [100.00-1000.00]
305.11682
R=46900
z=1
C 20 H 17 O 3 = 305.11722
-1.30973 ppm
185.10728
R=59100
z=1
C 10 H 17 O 3 = 185.11722
-53.67895 ppm
431.12863
R=38900
z=1
C 29 H 19 O 4 = 431.12779
1.96234 ppm
631.20831
R=32600
z=1
C 49 H 27 O = 631.20564
4.23153 ppm
935.31635
R=22800
z=?
C 9 H 4 O 12 Br 8 = 935.31643
-0.08821 ppm
Relative Abundance
m/z

6b

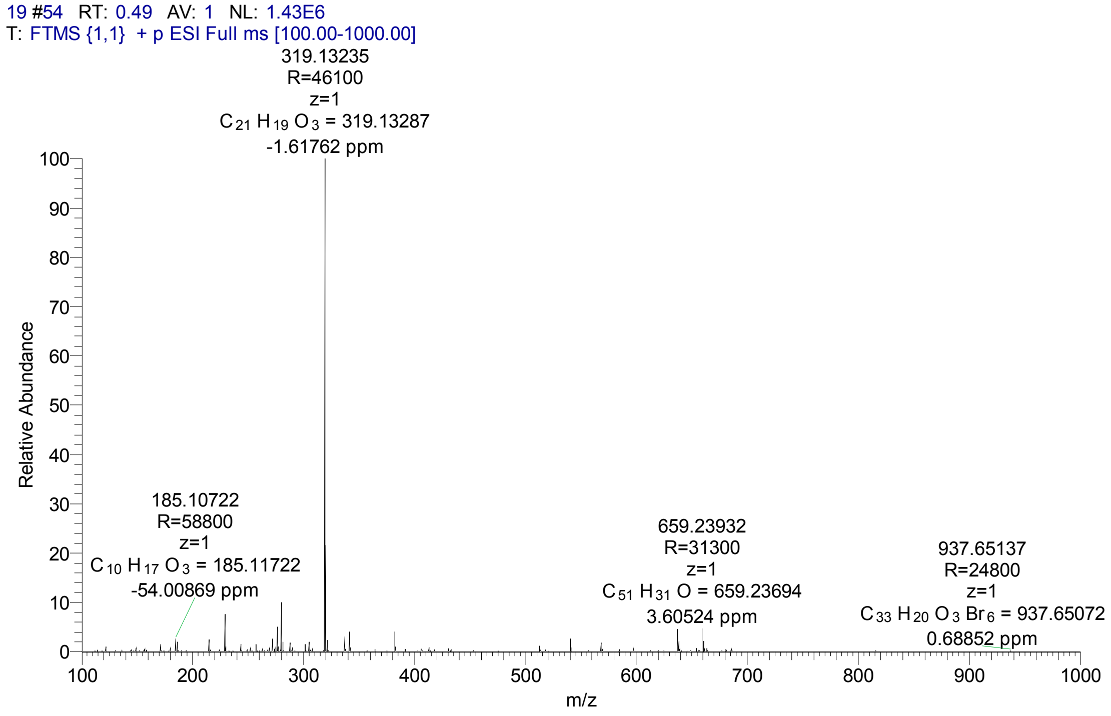
19 #54 RT: 0.49 AV: 1 NL: 1.43E6
T: FTMS {1,1} + p ESI Full ms [100.00-1000.00]
319.13235
R=46100
z=1
C 21 H 19 O 3 = 319.13287
-1.61762 ppm
185.10722
R=58800
z=1
C 10 H 17 O 3 = 185.11722
-54.00869 ppm
659.23932
R=31300
z=1
C 51 H 31 O = 659.23694
3.60524 ppm
937.65137
R=24800
z=1
C 33 H 20 O 3 Br 6 = 937.65072
0.68852 ppm
Relative Abundance
m/z

6c

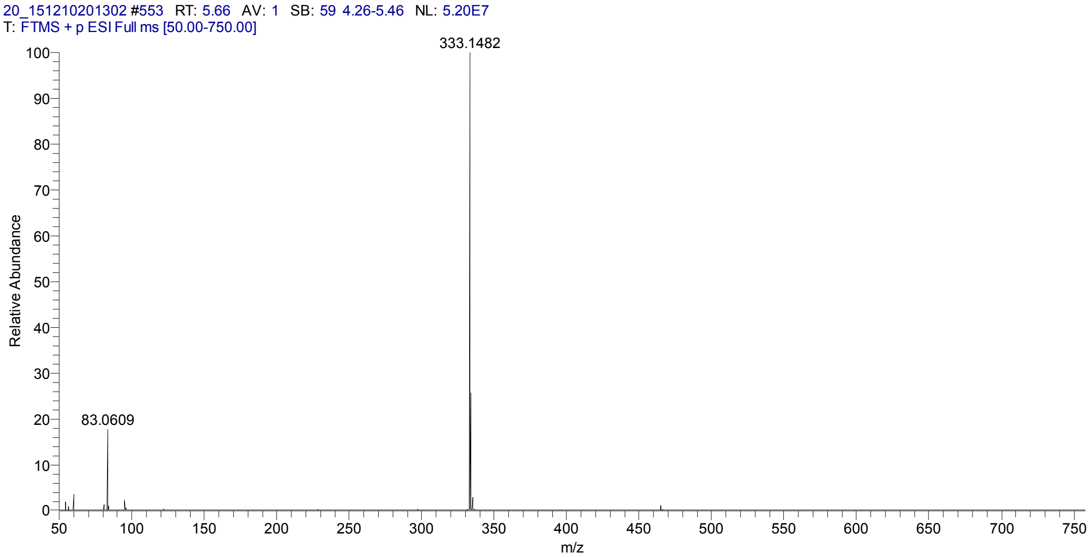
20_151210201302 #553 RT: 5.66 AV: 1 SB: 59 4.26-5.46 NL: 5.20E7
T: FTMS + p ESI Full ms [50.00-750.00]
333.1482
83.0609
Relative Abundance
m/z
0
10
20
30
40
50
60
70
80
90
100
50
100
150
200
250
300
350
400
450
500
550
600
650
700
750

6d

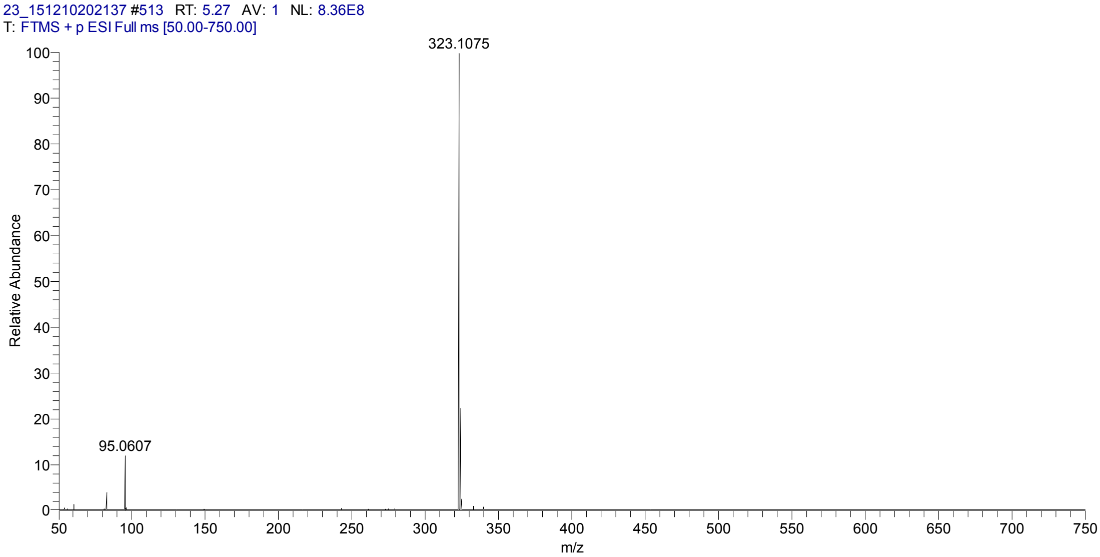
23_151210202137 #513 RT: 5.27 AV: 1 NL: 8.36E8
T: FTMS + p ESI Full ms [50.00-750.00]
323.1075
95.0607
Relative Abundance
m/z
0
10
20
30
40
50
60
70
80
90
100
50
100
150
200
250
300
350
400
450
500
550
600
650
700
750

6e

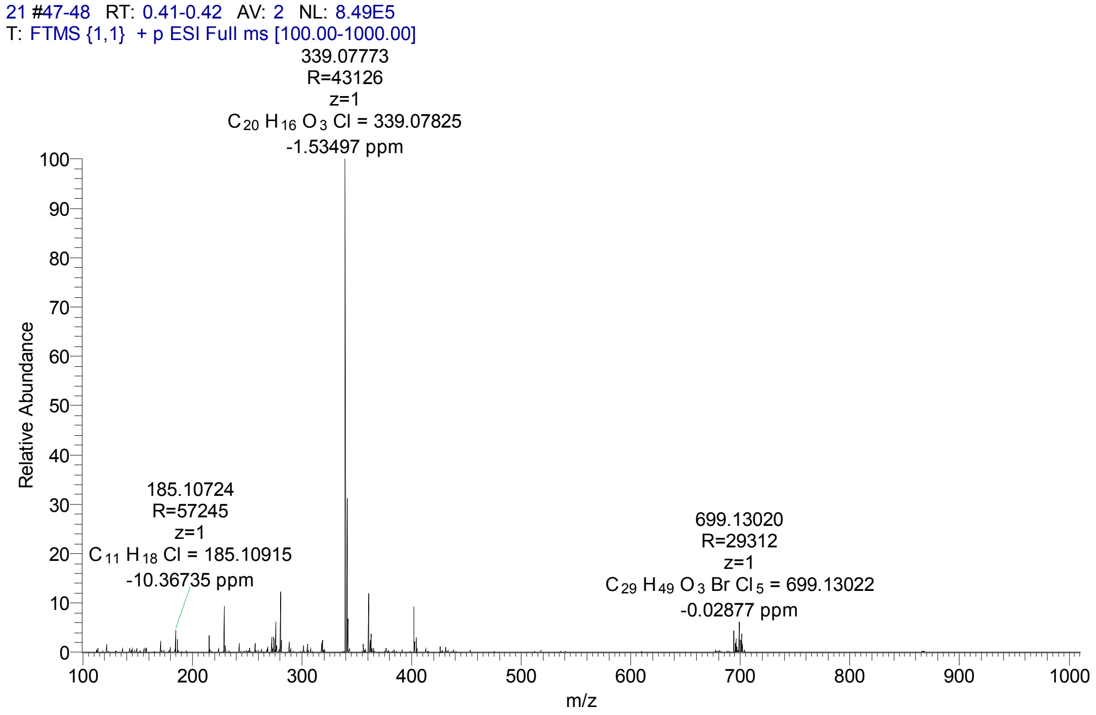
21 #47-48 RT: 0.41-0.42 AV: 2 NL: 8.49E5
T: FTMS {1,1} + p ESI Full ms [100.00-1000.00]
339.07773
R=43126
z=1
$C_{20}H_{16}O_3Cl$ = 339.07825
-1.53497 ppm
185.10724
R=57245
z=1
$C_{11}H_{18}Cl$ = 185.10915
-10.36735 ppm
699.13020
R=29312
z=1
$C_{29}H_{49}O_3BrCl_5$ = 699.13022
-0.02877 ppm
Relative Abundance
m/z

6f

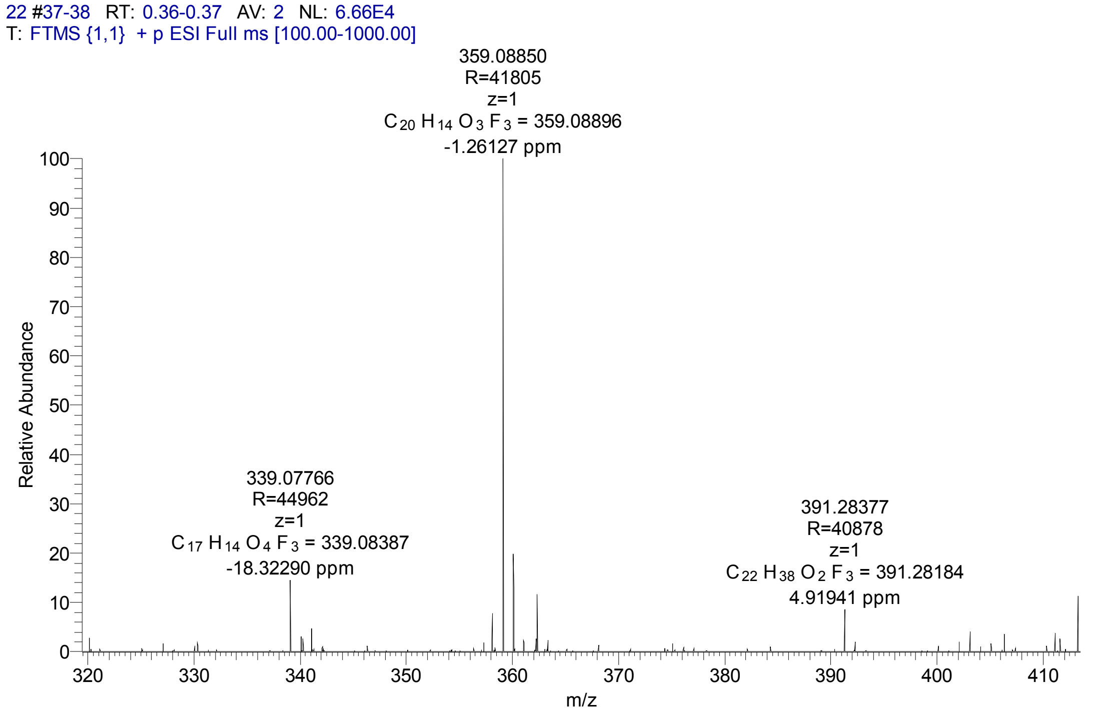
22 #37-38 RT: 0.36-0.37 AV: 2 NL: 6.66E4
T: FTMS {1,1} + p ESI Full ms [100.00-1000.00]
359.08850
R=41805
z=1
$C_{20}H_{14}O_3F_3$ = 359.08896
-1.26127 ppm
339.07766
R=44962
z=1
$C_{17}H_{14}O_4F_3$ = 339.08387
-18.32290 ppm
391.28377
R=40878
z=1
$C_{22}H_{38}O_2F_3$ = 391.28184
4.91941 ppm
Relative Abundance
m/z
